# Metagenome-wide analysis uncovers gut microbial signatures and implicates taxon-specific functions in end-stage renal disease

**DOI:** 10.1186/s13059-023-03056-y

**Published:** 2023-10-12

**Authors:** Pan Zhang, Xifan Wang, Shenghui Li, Xuesen Cao, Jianzhou Zou, Yi Fang, Yiqin Shi, Fangfang Xiang, Bo Shen, Yixuan Li, Bing Fang, Yue Zhang, Ruochun Guo, Qingbo Lv, Liwen Zhang, Yufei Lu, Yaqiong Wang, Jinbo Yu, Yeqing Xie, Ran Wang, Xiaohong Chen, Jiawei Yu, Zhen Zhang, Jingjing He, Jing Zhan, Wenlv Lv, Yuxin Nie, Jieru Cai, Xialian Xu, Jiachang Hu, Qi Zhang, Ting Gao, Xiaotian Jiang, Xiao Tan, Ning Xue, Yimei Wang, Yimei Ren, Li Wang, Han Zhang, Yichun Ning, Jing Chen, Lin Zhang, Shi Jin, Fazheng Ren, Stanislav Dusko Ehrlich, Liang Zhao, Xiaoqiang Ding

**Affiliations:** 1 Department of Nephrology, Zhongshan Hospital, Fudan University; Hemodialysis Quality Control Center of Shanghai; Shanghai Key Laboratory of Kidney and Blood Purification; Shanghai Institute for Kidney and Dialysis; Shanghai Clinical Medical Center for Kidney Disease, Shanghai, 200032 China; 2 Key Laboratory of Functional Dairy, Department of Nutrition and Health, China Agricultural University, Beijing, 100190 China; 3 Puensum Genetech Institute, Wuhan, 430076 China; 4 Key Laboratory of Precision Nutrition and Food Quality, Department of Nutrition and Health, China Agricultural University, Beijing, 100190 China; 5 Department of Clinical and Movement Neurosciences, UCL Queen Square Institute of Neurology, Queen Square, London, WC1N 3RX UK

## Abstract

**Background:**

The gut microbiota plays a crucial role in regulating host metabolism and producing uremic toxins in patients with end-stage renal disease (ESRD). Our objective is to advance toward a holistic understanding of the gut ecosystem and its functional capacity in such patients, which is still lacking.

**Results:**

Herein, we explore the gut microbiome of 378 hemodialytic ESRD patients and 290 healthy volunteers from two independent cohorts via deep metagenomic sequencing and metagenome-assembled-genome-based characterization of their feces. Our findings reveal fundamental alterations in the ESRD microbiome, characterized by a panel of 348 differentially abundant species, including ESRD-elevated representatives of *Blautia* spp., *Dorea* spp., and Eggerthellaceae, and ESRD-depleted *Prevotella* and *Roseburia* species. Through functional annotation of the ESRD-associated species, we uncover various taxon-specific functions linked to the disease, such as antimicrobial resistance, aromatic compound degradation, and biosynthesis of small bioactive molecules. Additionally, we show that the gut microbial composition can be utilized to predict serum uremic toxin concentrations, and based on this, we identify the key toxin-contributing species. Furthermore, our investigation extended to 47 additional non-dialyzed chronic kidney disease (CKD) patients, revealing a significant correlation between the abundance of ESRD-associated microbial signatures and CKD progression.

**Conclusion:**

This study delineates the taxonomic and functional landscapes and biomarkers of the ESRD microbiome. Understanding the role of gut microbiota in ESRD could open new avenues for therapeutic interventions and personalized treatment approaches in patients with this condition.

**Supplementary Information:**

The online version contains supplementary material available at 10.1186/s13059-023-03056-y.

## Background

End-stage renal disease (ESRD) is an advanced state of chronic kidney disease (CKD) defined by severe and irreversible kidney damage. CKD is a prevalent health issue globally, with estimated morbidity rates of 9.1% worldwide and approximately 12–15% in upper-middle-income countries [1, 2]. Approximately 2% of CKD patients progress to ESRD, resulting in a significant decline in quality of life, increased mortality risk, and substantial financial burden [3, 4]. Dialysis serves as the primary therapeutic modality for ESRD; however, patients with ESRD still experience uremia and uremic syndromes due to the accumulation of toxins between dialysis sessions and inadequate clearance of certain protein-bound toxins during dialysis [5]. Many of these toxins, such as indoxyl sulfate (IS), *p*-cresyl sulfate (PCS), phenylacetylglutamine (PAG), and trimethylamine N-oxide (TMAO), are derived from diverse gut bacteria through the fermentation of dietary proteins or cholines [6, 7]. Moreover, the gut microbial community helps maintain individual metabolic and immune homeostasis, which may be beneficial for reducing the risk of complications (e.g., constipation and cardiovascular disease) in ESRD patients [8–10]. Notably, recent reports have highlighted the association between gut dysbiosis and mortality in hemodialysis patients [11], emphasizing the significant contribution of gut microbiota to the etiology and treatment of ESRD.

Various approaches targeting the gut microbiota are currently being explored for the management and treatment of CKD and ESRD. For example, the supplementation of dietary fiber has shown promising results in reducing uremic toxin levels and cardiovascular risk in CKD patients [12]. Also, preliminary clinical trials in CKD and ESRD patients have demonstrated that probiotic, prebiotic, and synbiotic supplementation can lower uremic toxins, improve inflammatory mediators, and potentially slow down disease progression [13, 14]. Notably, a recent study highlighted the significant impact of oral administration of a single probiotic, *Lactobacillus casei Zhang*, which effectively increased the level of short-chain fatty acids (SCFAs) and nicotinamide in the serum and kidney and retarded the decline of kidney function in CKD [15]. While these interventions have resulted in modifications of the gut microbiota or specific gut microbial taxa, the precise impact of these alterations on the disease and the underlying mechanisms involved remain unclear. Nevertheless, unraveling the patterns of gut microbiota in ESRD patients holds great potential for enhancing our understanding of the disease mechanisms and advancing therapeutic approaches.

Several studies have investigated alterations in the gut microbiome of ESRD patients using sequencing of the bacterial 16S rRNA gene; however, these studies were limited by relatively small sample sizes [16–19]. As summarized by Zhao et al. [20], these studies consistently indicated an altered gut microbiota in ESRD patients, characterized by a decrease in microbial diversity, an expansion of certain pathogenic microorganisms (e.g., *Fusobacterium* spp. and members of Enterobacteriaceae), and a depletion of symbiotic taxa (e.g., *Prevotella* spp. and *Faecalibacterium* spp.). In our previous study, we revealed the dependence between the gut microbiota and uremic toxin accumulation in the feces and serum of ESRD patients and confirmed that the patient’s gut microbiota and several ESRD-associated species can promote toxin production in a CKD mouse model [21]. However, the microbial signatures at the low taxonomic levels (e.g., at the species or strain level) and the unique functional capacity of ESRD-associated species have not been fully characterized. Furthermore, the generalizability of these findings has not been tested in an independent ESRD patient cohort or in a cohort of CKD patients who do not require dialysis.

To overcome these limitations, this study aimed to conduct a comprehensive investigation on a larger scale. We performed metagenomic shotgun sequencing to analyze a total of 715 fecal samples, including 378 hemodialytic patients, 47 non-dialyzed CKD patients, and 290 healthy volunteers. These samples were obtained from two distinct Chinese cohorts: the previously described Beijing cohort [21], which was extended to include new ESRD patients and healthy controls (totaling 254 ESRD patients and 179 controls), and a newly established Shanghai cohort (124 ESRD patients, 47 non-dialyzed CKD patients, and 111 controls). We employed both genome-centric and gene-centric strategies to characterize the taxonomic and functional variations in the ESRD microbiome. Furthermore, we linked species/taxon-specific functions to the ESRD status by performing functional annotation of the ESRD-associated species at the genome level. To validate the relationship between the gut microbiota and uremic toxins, we determined the serum concentrations of four specific uremic toxins (i.e., IS, PCS, PAG, and TMAO) in the Shanghai cohort using a targeted approach. These data were then combined with the metabolome data from the Beijing cohort to enable cross-cohort validation of the microbiota-toxin relationship.

## Results

### Participants and dataset

Two cohorts were studied, from Shanghai and Beijing. The Shanghai cohort included 124 hemodialysis patients, 47 non-dialyzed CKD patients, and 111 healthy volunteers. The Beijing cohort consisted of participants from our previous study [21] (223 hemodialysis patients and 69 healthy volunteers) and an additional 31 hemodialysis patients and 110 healthy volunteers. For both cohorts, no significant difference was observed in sex, age, body mass index (BMI), or dietary habits between the patients and controls (see Additional file 1: Table S1 for the phenotypic information of all participants). The ESRD patients in both cohorts underwent hemodialysis 1–3 times per week, with a duration ranging from 6 to 264 (median 52) for the Shanghai cohort and from 6 to 312 (median 36) months for the Beijing cohort. Importantly, the recruitment, sample collection and storage were conducted independently for the Shanghai and Beijing cohorts to ensure mutual verifiability.

Whole-metagenome shotgun sequencing of 715 fecal samples was carried out to generate a total of 8.8 trillion bases (Tb) of high-quality data (including 3.3 Tb from our previous study [21]), with an average of 12.3 ± 2.0 gigabases (Gb) of data per sample (Additional file 1: Table S2-3). Reads assembly yielded 19,391 medium- and high-quality metagenome-assembled genomes (MAGs) with completeness of > 70%, contamination of < 5%, and quality score of > 60. These MAGs were further grouped into 1303 clusters (“species” hereafter) at the nucleotide similarity threshold of 95% [22]. On average, these species had a genome completeness of 93.0% (median, 96.0%) and a contamination rate of 0.8% (Additional file 1: Table S4). Notably, 164 of these species were not present in the comprehensive Unified Human Gastrointestinal Genome (UHGG) collection [23] and were considered novel. We annotated 1303 species using the Genome Taxonomy Database (GTDB) [24], with manual revision, and they represented the major phylogenetic taxa of the human gut microbiota (Supplementary Fig. 1; Additional file 1: Table S4; Additional file 2: Fig. S1). A majority (73.1%) of these species were derived from uncultured microbes, similar to the entire UHGG collection. Mapping the metagenomic reads against the collection of species revealed clear species boundaries, with a high mapping rate on average (81.6% per sample, Additional file 2: Fig. S2), indicating that the assembled species effectively represented the microbiome of our cohort.

### The gut microbiome stratifies ESRD patients from healthy controls

**Fig. 1:**
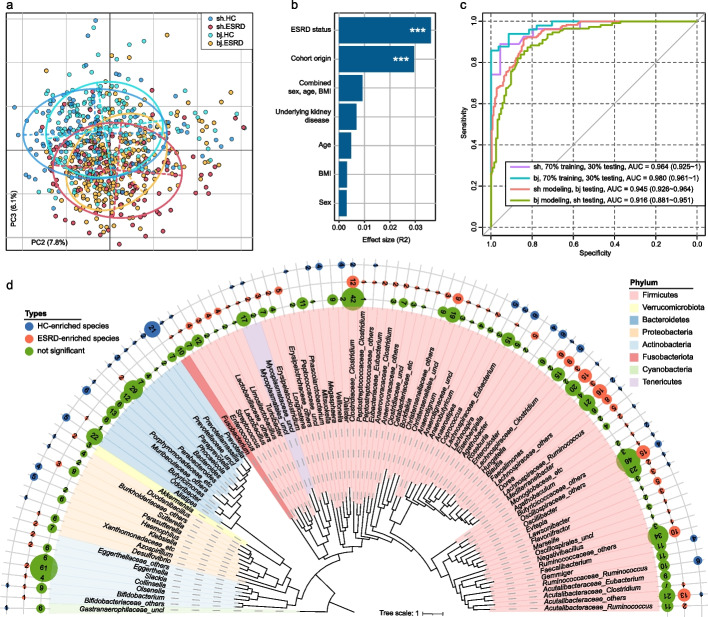
Distinct gut microbiome characteristics are associated with ESRD. **a** Principal coordinates analysis (PCoA) of the Bray–Curtis distances based on gut microbial species. Samples are depicted in the plot at the second and third principal coordinates (PC2 and PC3), along with the ratio of variance contributed by these two PCs. Ellipsoids represent a 95% confidence interval surrounding each group. **b** Permutational multivariate analysis of variance (PERMANOVA) results showing the effect size of phenotype indexes contributing to the variance of the overall gut microbiome. The combined effect size of sex, age, and body mass index (BMI) is also shown. Bar plots indicate the explained variation (effect size *R*^2^) of each phenotype factor. ***, permutated *p* < 0.001. **c** Receiver operating characteristic (ROC) analysis of the classification of ESRD status using the random forest model. For each cohort, 70% of the samples were randomly selected as the training set, and the remaining 30% of the samples were used as the testing set. The performances of models trained on one cohort and tested on the other are also shown. The classification performance of the model was assessed using the area under the ROC curve (AUC). The AUC values and 95% confidence intervals (CIs) are shown. **d** Overall representation of the 353 ESRD-associated species with a consistent trend observed in both the Shanghai and Beijing cohorts. The numbers of ESRD-enriched and HC-enriched species for each genus are shown

The species richness and evenness of the ESRD patients were approximately equal to those of the healthy controls in both cohorts (Additional file 2: Fig. S3). However, principal coordinates analysis (PCoA) of Bray–Curtis distance revealed a clear distinction between patients and controls (Fig. 1a). Further analysis of the top five principal coordinates (PCs) (with a contribution > 3% and *p* < 0.05 in the Tracy–Widom test) demonstrated that PC1, PC2, PC3, and PC5 were significantly correlated with ESRD status, while PC1, PC4, and PC5 showed differences between the cohorts (Additional file 2: Fig. S4). These findings suggested that both the disease status and the cohort origin were major factors driving the variations in gut microbial composition in our samples. Consistently, permutational multivariate analysis of variance (PERMANOVA) indicated that ESRD status and cohort origin independently accounted for 3.61% (*p* < 0.001) and 2.95% (*p* < 0.001) of the overall microbial variability, respectively. The combined effects of individuals’ sex, age, and BMI explained an additional 0.92% of the variation (effect size < 0.5% and *p* > 0.05 for each parameter; Fig. 1b). Interestingly, the underlying kidney disease (e.g., glomerulonephritis or diabetic kidney disease) only contributed to 0.68% (*p* = 0.23) of the gut microbial variations among ESRD patients (Fig. 1b).

To assess the discriminatory power of the gut microbiome in ESRD patients and healthy controls, we employed the random forest regression model, which exhibited high accuracy in identifying ESRD status in both the Shanghai cohort (area under the receiver operating characteristic curve [AUC], 0.96) and the Beijing cohort (AUC, 0.98) (Fig. 1c; Additional file 1: Table S5). Notably, the models trained on one cohort showed robust discriminatory power in classifying patients and controls in the other cohort (AUC > 0.91; Additional file 2: Fig. S5). Taken together, our results indicate consistent alterations of the gut microbiome of ESRD patients, notwithstanding the significant difference of the overall microbiome composition of the two cohorts, and suggest associations of microbiome alterations and kidney disease.

### Microbial species related to ESRD

Some 353 differentially abundant species between the ESRD patients and healthy controls were identified using the combined significance level in the two cohorts (combined Wilcoxon rank-sum test *q* < 0.05; Additional file 2: Fig. S6a-b). Among these, 224 were more abundant in patients and 129 in controls. Importantly, all of these species remained significant upon adjustment for sex, age, and BMI, with a majority (73.5%) having a significant (*q* < 0.05) and coherent enrichment in both cohorts (Additional file 1: Table S6). The enrichment of species was mainly consistent at the phylum level, as 41 out of 44 significantly different Bacteroidetes species and 12 out of 18 Proteobacteria species enriched in healthy controls. Conversely, a large majority of Firmicutes species (200 out of 271, 73.8%) and 9 out of 10 Actinobacteria species were enriched in ESRD patients (Fig. 1d; Additional file 2: Fig. S6c). Of particular interest among the health control (HC)-enriched Firmicutes species were *Roseburia spp.* (*n* = 6) and *Faecalibacterium spp.* (*n* = 4), known for their butyrate production [25], as well as *Lachnospira spp.* (*n* = 5), and all Negativicutes species (*n* = 10). Among the Bacteroidetes enriched in controls, the most common clade was Prevotellaceae, represented by 25 species, with all except *Prevotella copri* remaining uncharacterized at a species level. On the other hand, the ESRD-enriched Firmicutes species often belonged to closely related clades, including *Blautia* (*n* = 15), *Dorea* (*n* = 8), *Enterocloster* (*n* = 6), *Lawsonibacter* (*n* = 4), and *Eisenbergiella* (*n* = 5). Among the ESRD-enriched Actinobacteria members were four Eggerthellaceae species, including a previously described ESRD-related species *Eggerthella lenta* [21].

### Microbial functions related to ESRD

**Fig. 2:**
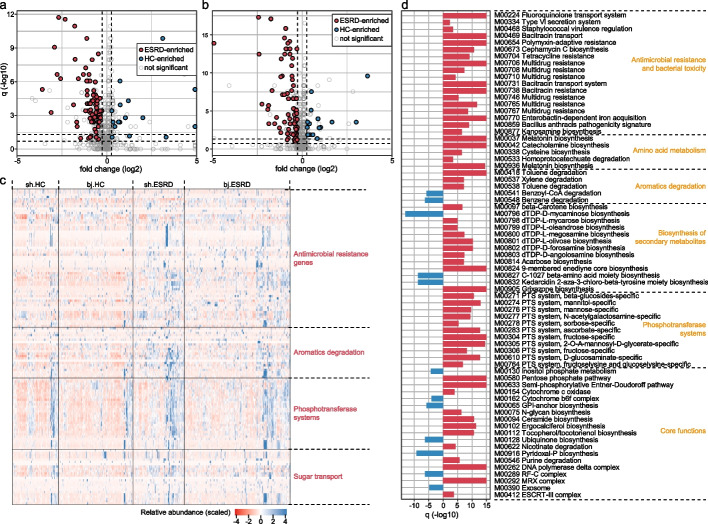
Functional profiles differ between ESRD patients and healthy controls. **a**, **b** Volcano plots displaying the fold change vs. *q*-values for all KEGG modules in the Shanghai (**a**) and Beijing (**b**) cohorts. The *X*-axis shows the ratio (log2 transformed) of module abundance in ESRD patients compared with that in healthy controls. The *Y*-axis shows the *q*-value (-log10 transformed) of a module, and the two dotted lines indicate *q*-value < 0.05 (upper) and < 0.2 (lower). The ESRD-associated modules with a consistent trend in the two cohorts are shown in red (ESRD enriched) and blue (control enriched) circles. **c** Heatmap showing the abundance of several representative functions in ESRD patients and healthy controls. Each row represents a KEGG ortholog with the scaled relative abundance among all individuals and each column represents an individual, ordered by hierarchical clustering. The results of four ESRD-enriched representative categories of enzymes are shown. **d** Bar plot showing the *q*-values (log10 transformed) of ESRD-associated modules. Red, ESRD-enriched modules; blue, HC-enriched modules

To characterize the functional features of ESRD microbiota, we annotated the microbial functions of each metagenome via the KEGG (The Kyoto Encyclopedia of Genes and Genomes) database using a gene-centric approach [26]. A total of 10,254 KEGG orthologs (KOs) and 806 modules were identified and used for further analysis. Like the taxonomic composition, the overall functional capacity of ESRD patients’ microbiota exhibited a considerable shift compared to that of healthy controls (Additional file 2: Fig. S7). A total of 1279 KOs and 103 modules differed significantly in abundance between the patients and controls when combining the Shanghai and Beijing cohorts (Fig. 2a, b; Additional file 2: Fig. S8). A majority (75.8% of KOs and 81.6% of modules) of the differential functions were enriched in the gut microbiome of ESRD patients. Modules that were most highly ESRD-biased involved antimicrobial resistance and bacterial toxicity, phosphotransferase systems for monosaccharide (e.g., fructose and mannose) uptake, secondary metabolite biosynthesis, and transport systems (Fig. 2c, d; Additional file 1: Table S7-8). Conversely, the ESRD-depleted functions included inositol phosphate metabolism, ubiquinone and pyridoxal biosynthesis, and others. It is worth mentioning that the biodegradation of aromatic compounds by gut bacteria is a major source of uremic toxins [27, 28]. In ESRD patients, several modules related to the degradation of aromatic amino acids (AAAs) (i.e., M00037 and M00936 for tryptophan and M00042 for tyrosine) or other aromatics (i.e., M00418 and M00538 for toluene and M00537 for xylene) were enriched, whereas modules involved in the degradation of benzene (M00548) and benzoyl-CoA (M00541) were depleted. Consistently, the enzymes associated with aromatic degradation were predominantly enriched (25/27 KOs) in the ESRD microbiome (Additional file 1: Table S7).

Considering the incomplete representation of antibiotic resistance genes (ARGs) in the KEGG database, we annotated and profiled a total of 3009 ARGs using a comprehensive approach (see Methods) to investigate the alteration of antibiotic resistome in ESRD (Additional file 1: Table S9). The ESRD patients exhibited an average 39.4% increase in the overall relative abundance of ARGs compared with the healthy controls (*p* < 0.001; Additional file 2: Fig. S9a-b). This increase was particularly notable for several antibiotic resistance mechanisms, including multi-drug resistance, beta-lactamase, aminoglycoside, and quinolone resistance (Additional file 2: Fig. S9c). A similar phenomenon was observed in non-dialyzed CKD patients (Additional file 2: Fig. S9b-c). These findings suggest that patients may have experienced more frequent exposure to antibiotics in the past than the controls [29].

### Functional configuration of ESRD-enriched and HC-enriched species

**Fig. 3:**
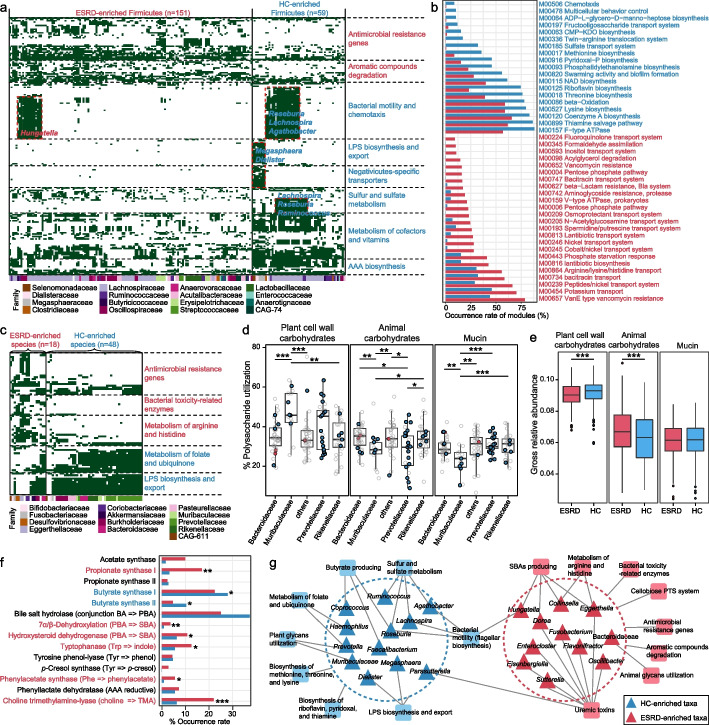
Overview of the functional configuration of the ESRD-associated species. **a** and **c** Heatmap showing the occurrence of several types of enzymes in ESRD-associated Firmicutes (**a**) and non-Firmicutes (**c**) species. Each row represents a KEGG ortholog, while each column represents a species (colored based on their family-level taxonomic assignment). Green and white panels indicate the presence and absence of the enzymes in each species, respectively. Several representative functions and the corresponding species are labeled in the heatmap. **b** Bar plots showing the occurrence rate of KEGG modules in the ESRD-associated Firmicutes species. Forty KEGG modules that differed in integrity between ESRD-enriched and HC-enriched Firmicutes species (see Additional file 1: Table S10 for details) are shown. The colored bars show the percentage of species that contained complete KEGG modules: red, module present in ESRD-enriched species; blue, module present in HC-enriched species. For **a**–**c**, text colored in red and blue denotes enrichment in ESRD patients and healthy controls, respectively. **d** Boxplot showing the polysaccharide utilization capacity, including animal carbohydrates, plant cell wall carbohydrates, and mucin utilization, of Bacteroidetes species. Nodes represent the species and its colors represent the enrichment of the species: red, enriched in ESRD patients; blue, enriched in healthy controls; gray, not significant. **e** Boxplot showing the comparison of enzymes involving to polysaccharide utilization in the gut microbiome of ESRD patients and healthy controls. **f** Occurrence rate of enzymes involved in the biosynthesis of SCFAs, bile acids, and uremic toxins in the ESRD-enriched and HC-enriched species. Red, ESRD-enriched species; blue, HC-enriched species. Fisher’s exact test: *q < 0.05; **q < 0.01; ***q < 0.001. **g** Schematic diagram depicting the interconnection of ESRD-associated taxa and their functions. Colored triangles denote the taxa that were mostly enriched in ESRD patients (red) or healthy controls (blue), and colored squares denote the functions that were mainly encoded by ESRD-enriched (red) and HC-enriched (blue) species. Connections between the taxa and functions suggest that the functions are likely encoded by the corresponding species

In contrast to conventional metagenomic strategies used to study microbial functions in the context of a mixed microbial community, our MAG-based approach offers the primary advantage of having microbial genomes, enabling the assignment of functions to species and thus linking the species-specific functions to ESRD. Using this approach, we functionally annotated 276 out of 353 ESRD-associated species with completeness > 90% (“near-complete” genomes [30]) and computed the functional differences between the ESRD- and HC-enriched species. To minimize the impact of phylogeny, which can influence functional contents (Additional file 2: Fig. S10), we first performed the comparison for Firmicutes. The occurrence frequency of 730 KOs differed significantly between ESRD- and HC-enriched firmicutes (Fisher’s exact test, *q* < 0.05; Additional file 1: Table S10). These KOs mainly fell into several categories, with enzymes involved in antimicrobial resistance and degradation of aromatic compounds being more widespread in ESRD-enriched species, while those associated with cofactor and vitamin metabolism (e.g., porphyrin, thiamine, and folate) and AAA biosynthesis were mostly present in HC-enriched species (Fig. 3a). Notably, sulfur and sulfate metabolism (encoded by *Lachnospira* and *Roseburia spp.*) and lipopolysaccharide (LPS) biosynthesis (encoded by Negativicutes members) occurred uniquely in HC-enriched species (Additional file 2: Fig. S11a-b). Proteins associated with bacterial motility (flagellar biosynthesis) were predominantly encoded by HC-enriched *Lachnospira*, *Roseburia*, and *Agathobacter* species and ESRD-enriched *Hungatella* species (Additional file 2: Fig. S11c-d). At the KEGG module level, 40 modules differed in completeness between ESRD-enriched and HC-enriched Firmicutes (Fisher’s exact test, *q* < 0.05; Additional file 1: Table S11). Consistently with the findings from the gene-centric KO profiling, 8 modules of antimicrobial resistance were more frequently encoded in ESRD-enriched species, while 6 modules associated with the biosynthesis of some amino acids (methionine, threonine, and lysine) and vitamins (riboflavin, pyridoxal, and thiamine) were more often encoded in HC-enriched species (Fig. 3b). These findings suggest potential connections between these taxon-specific functions and ESRD.

Beyond Firmicutes, we observed significant functional differences between ESRD-enriched and HC-enriched species belonging to other phyla. The non-Firmicutes ESRD-enriched species exhibited a higher prevalence of antibiotic resistance genes, bacterial toxicity-related enzymes, and enzymes involved in arginine and histidine metabolism, while the HC-enriched species encoded a higher presence of enzymes involved in the metabolism of folate, ubiquinone, and LPS (Fig. 3c; Additional file 2: Fig. S12); these functional differences may potentially be associated with ESRD.

We then focused on the polysaccharide utilization capacity of the Bacteroidetes species since they are primary polysaccharide degraders in the human gut [31]. Genomic annotation of carbohydrate-active enzymes (CAZymes) revealed that, consistent with previous studies [32, 33], Bacteroidaceae species were more likely to utilize animal glycans, whereas Prevotellaceae and Muribaculaceae members (most of which were HC-enriched) were more likely to use plant glycans such as xylan, starch, and pectin (Fig. 3d; see Additional file 2: Fig. S13 for polysaccharide utilization locus analysis). Expanding the analysis to include all CAZyme genes found in fecal metagenomes, in addition to those carried by the assembled genomes, revealed that the microbiome of healthy controls had a higher abundance of plant polysaccharide-degrading genes (Fig. 3e). Interestingly, there was no strong correlation between polysaccharide utilization capacity and the relative abundances of Prevotellaceae/Muribaculaceae with meat or vegetable consumption (Additional file 2: Fig. S14), suggesting that the correlation between ESRD status and polysaccharide utilization is not highly dependent on diet.

Lastly, we focused on the ability of the ESRD-associated species to produce SCFAs, secondary bile acids (SBAs), and uremic toxins, as these functions/metabolites had been strongly linked to CKD and ESRD [17, 21, 34]. We observed that enzymes involved in butyrate synthesis were more prevalent in HC-enriched species, particularly in the typical butyrate producers like *Roseburia*, *Faecalibacterium*, and *Coprococcus spp.* [25], while acetate and propionate synthases were more common in ESRD-enriched species (Fig. 3f; Additional file 2: Fig. S15a). The production of SBAs was more widely distributed among ESRD-enriched species, including members of Lachnospiraceae (e.g., *Dorea* and *Hungatella spp.*) and Actinobacteria (*Collinsella intestinalis* and *E. lenta*) (Additional file 2: Fig. S15b). Regarding uremic toxins, the key enzymes involved in synthesizing precursors of IS, PAG, and TMAO were significantly more prevalent in ESRD-enriched species compared with HC-enriched species (*p* < 0.05 for all), while the *p*-Cresol (precursor of PCS) synthase was only encoded by ESRD-enriched species, although not significant (*p* = 0.08) (Fig. 3f). Additionally, we examined the abundance of these toxin-producing enzymes in fecal metagenomes and found that almost all enzymes were highly abundant in the microbiome of ESRD patients compared with that of controls (Additional file 2: Fig. 16a). At the species level, certain ESRD-enriched species belonging to Oscillospiraceae (e.g., *Oscillibacter* and *Flavonifractor spp.*), Lachnospiraceae (e.g., *Blautia spp.*, *Faecalimonas nexilis*, and *Hungatella effluvii*), and *Eggerthella* were among the major contributors to toxin synthesis (Additional file 2: Fig. 16b).

Collectively, these results uncovered the taxon-specific functional signals of ESRD-associated species and indicated potential mechanistic connections (Fig. 3g), which could guide future intervention studies.

### Gut bacteria affect serum uremic toxin levels

**Fig. 4:**
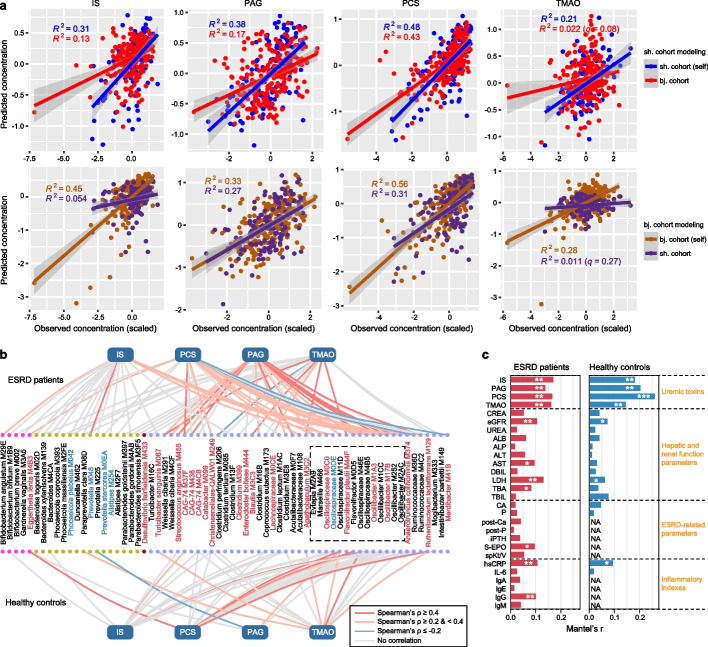
Gut microbiota-based prediction of the serum concentrations of uremic toxins. **a** Prediction of the serum concentrations of four toxins using gut microbiota-based model training with Shanghai individuals (upper panels) and Beijing individuals (bottom panels). For each model, 70% of the samples in one cohort were randomly selected as the training set, and the models were tested on the remaining 30% of the samples from the same cohort (self) or all samples from the other cohort. The *X*-axis and *Y*-axis show the predicted concentration and scaled measured concentration for each toxin, respectively. Smooth curves are formed using the *geom_smooth* function with the default parameters in the R *ggplot2* package. **b** The 67 gut species with the highest contribution to serum toxin levels. Lines connect the species used in the prediction models for each toxin, with the line color representing the Spearman correlation coefficient between the measured toxin concentration and species abundance. The color of the species denotes their enrichment in ESRD patients (red) or healthy controls (blue), and a dotted box labels the species that belong to Oscillospiraceae. Colored dots denote the species’ phylum (from left to right: Actinobacteria, Bacteroides, Proteobacteria, and Firmicutes). **c** Correlation between the 67 toxin-contributing species and the clinical parameters. The bar lengths indicate Mantel’s *r* between the species abundance and each parameter, and the *q*-values are calculated based on the Mantel test with 9999 permutations: *q < 0.05; **q < 0.01; ***q < 0.001

Our previous study [21] has suggested that the gut microbiota may drive the serum concentrations of multiple uremic toxins. To test this finding in an independent cohort, we quantified the serum concentrations of four major toxins (IS, PCS, PAG, and TMAO) in the Shanghai cohort, as described in the “Methods” section. We developed gut microbiota-based regression models to predict the serum concentrations of these toxins in ESRD patients (see the “Methods” section). These models demonstrated a substantial ability to explain the variance in toxin concentrations within each cohort (21.3–47.8% for the Shanghai cohort and 27.9–56.4% for the Beijing cohort; Fig. 4a; Additional file 2: Fig. S17). Importantly, the models trained on one cohort exhibited an average of 22.7% (ranging from 5.4 to 43.1%, *q* < 0.01 for all) of the concentration variances of three AAA-derived toxins (i.e., IS, PCS, and PAG) in the other cohort. This indicates that the gut microbiota affects the serum concentrations of these toxins to a considerable degree across different populations and quantification methods. The exception was TMAO, for which only 1.1–2.2% of the concentration variances were explained by models trained on the opposite cohort (Fig. 4a), probably due, in part, to batch effects or its high dialytic clearance rate [35] or dietary contributions to its levels [36].

Furthermore, after combining the data from ESRD patients in both cohorts, we identified 67 species that contributed the most to toxin concentrations (Fig. 4b; Additional file 1: Table S12). Among these “toxin-contributing species,” 21 were enriched in ESRD patients, while 5 were depleted. Fourteen of the 67 species belonged to the Oscillospiraceae family, coherently with the functional analysis. These 67 species also demonstrated moderate performances in predicting the serum concentrations of toxins in healthy subjects across both cohorts, explaining 10.7–39.0% of the variance for three AAA-derived toxins and 3.1% for TMAO (Fig. 4b; Additional file 2: Fig. S18). In addition, the Mantel test analysis revealed significant correlations between the composition of these toxin-contributing species and clinical parameters such as high-sensitivity C-reactive protein (hsCRP) and estimated glomerular filtration rate (eGFR) (Fig. 4c), suggesting their potential roles in human physiological health.

### Gut microbiota in non-dialyzed CKD patients

**Fig. 5:**
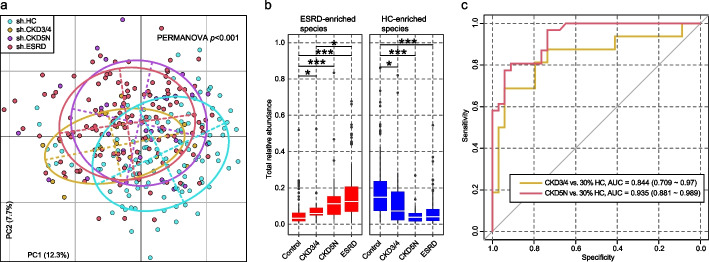
The gut microbial signatures of CKD patients are similar to those of ESRD patients. **a** Principal coordinates analysis (PCoA) of Bray–Curtis distances of gut microbial profiles of the Shanghai individuals. Samples are plotted based on the first and second principal coordinates (PC1 and PC2), with the ratio of variance contributed by these two PCs displayed. Ellipsoids represent a 95% confidence interval surrounding each group. **b** Total relative abundances of ESRD-enriched and HC-enriched species in the gut microbiota of all groups. Boxes represent the interquartile range between the first and third quartiles and the median (internal line). Whiskers denote the lowest and highest values within 1.5 times the range of the first and third quartiles, respectively; dots represent outlier samples beyond the whiskers. Student’s *t*-test: *q < 0.05; **q < 0.01; ***q < 0.001. **c** Random forest models for distinguishing CKD3/4 and CKD5N patients from healthy controls based on the gut species profile of ESRD patients. This analysis was performed on the Shanghai cohort, where 70% of control (randomly selected) and all ESRD samples were used as the training set, and the remaining 30% of control and all CKD3/4 and CKD5N samples were used as the testing set. The classification performance of the model was assessed by the AUC. The AUC values and 95% confidence intervals (CIs) are shown. AUC, the area under the ROC curve

To test whether the gut microbial characteristics of ESRD can be observed already in CKD patients, we analyzed the fecal metagenomes of 16 patients with CKD stages 3–4 (CKD3/4) and 31 patients with CKD stage 5 (CKD5N) from the Shanghai cohort. PCoA and PERMANOVA analyses revealed that the gut microbiome composition of CKD3/4 patients fell between that of the ESRD patients and healthy controls (CKD3/4 vs. ESRD, effect size = 3.2%, *adonis p* = 0.013; CKD3/4 vs. HC, effect size = 5.7%, *adonis p* = 0.002), while the microbiome of CKD5N patients was not statistically different from that of ESRD patients (*adonis p* = 0.19) (Fig. 5a). A majority of the 353 ESRD-associated species displayed similar abundance trends in CKD3/4 and CKD5N patients compared with healthy controls, among these, 82 species (53 ESRD-enriched and 29 HC-enriched species) showed a continuous positive or negative trend in relation to disease severity (Additional file 1: Table S13; Additional file 2: Fig. S19). Also, the total relative abundances of the ESRD-enriched and -depleted species showed significant correlations with the severity of CKD (Fig. 5b). These findings support the hypothesis that ESRD-associated bacteria might be related to CKD progression and the development of ESRD.

Consistently, a random forest classifier based on the microbial profiles of ESRD patients and controls in the Shanghai cohort achieved AUCs of 0.84 and 0.94 in discriminating CKD3/4 and with CKD5N patients, respectively, from the healthy controls (Fig. 5c). The classifier trained on the Beijing cohort achieved similar performance (Additional file 2: Fig. S20). These findings illustrate the diagnostic potential of ESRD microbial signatures in CKD patients.

## Discussion

Here, we studied the gut microbiome composition of two Chinese cohorts, consisting of a total of 378 ESRD patients and 290 healthy controls, using shotgun metagenomics and identified 353 species differentially abundant between patients and controls. Our findings confirmed the majority of previous observations (as summarized by Zhang et al. [20]) at the genus level, such as the enrichment of *Streptococcus*, *Fusobacterium*, and *Desulfovibrionaceae*, as well as the depletion of *Prevotella*, *Faecalibacterium*, and *Coprococcus* in ESRD patients. Notably, our study extended these observations to the MAG-based species level, providing more detailed insights. For instance, within the *Prevotella* genus, which encompasses over 30 species in the human gut [37], we identified 21 species that showed significant depletion in ESRD patients. Among them were two clades of *P. copri* (*P. copri* is a complex with 4 species-level clades [38]), *P. stercorea*, *P. lascolaii*, and 17 uncultivated species; further functional analyses are needed to elucidate their possible role in ESRD. Beyond previously observed ESRD-associated microbiome alterations, we also report numerous novel alterations, leveraging the large sample size and cross-regional verification. These newly identified taxa include ESRD-enriched members of Oscillospiraceae (including the flavonoid degrader *Flavonifractor* [39]) and *Dorea* (potential producer of SBAs and toxins), as well as ESRD-depleted members of Muribaculaceae species (potential plant polysaccharide degraders) and *Lachnospira* (potential SCFA producers). Besides, we found the consistency of these ESRD-associated signatures in patients with CKD, indicating that these signatures may influence the progression of CKD.

In addition, we have elucidated the functional profiles of 353 ESRD-associated species using a genome-centric approach, which provides a significant advancement compared to previous studies that typically employed the gene-centric approach [26] or clade-specific markers. The availability of functional profiles allowed us to connect numerous species/taxon-specific functions to ESRD, as summarized in Fig. 3g, thus laying grounds for a better understanding of bacteria-host crosstalk mechanisms in disease. For instance, we found that the proteins responsible for flagellar biosynthesis were more likely to be encoded by HC-enriched members of *Lachnospira*, *Roseburia*, and *Agathobacter*, as well as ESRD-enriched *Hungatella* spp. These species produce flagellin, which has previously been demonstrated to possess immunomodulatory properties associated with multiple inflammatory diseases [40, 41]. In particular, *Roseburia*-derived flagellin has been shown to act as a negative regulator of gut inflammation [42] and exhibits protection effects on barrier functions [43]. These findings suggest that the flagellar/flagellin biosynthesis capacity may hold potential significance in the context of ESRD, warranting further investigation in this research direction. Another example pertains to the members of Prevotellaceae (comprising 25 HC-enriched species) and Muribaculaceae (6 HC-enriched species). Functional analysis indicated that these species contribute to a higher capacity for plant polysaccharide utilization within the gut microbiome of healthy controls compared to ESRD patients. The microbial conversion of dietary plant polysaccharides (e.g., glycans, fiber) into SCFAs serves as an important energy and signaling process that impacts the maintenance and functionality of the gut microbiota [44] and exhibits beneficial effects on kidney diseases [45, 46]. Consequently, our findings support the potential benefit of dietary fiber supplementation or other prebiotic interventions in ESRD patients to optimize their gut microbiota. Furthermore, these findings suggest that the gut Prevotellaceae and Muribaculaceae members could be potential targets of such interventions.

Overall, comparisons of the functional profiles of the ESRD-enriched and HC-enriched species led to two noteworthy findings, overabundance of enzymes/modules involved in antimicrobial resistance, aromatic compound degradation, and SBA and uremic toxin production in ESRD-enriched species and enrichment of functions involving bacterial motility (anti-inflammatory flagellin synthesis), sulfur metabolism, plant polysaccharide degradation, and biosynthesis of small bioactive molecules (e.g., certain amino acids, vitamins, and SCFAs) in HC-enriched species. These findings suggest a dysbiotic microbiome and inflammation-associated characteristics in ESRD. While the precise identification of the most influential species or functions in ESRD remains elusive, the results of our study provide guidance for subsequent cultivation of the identified species and their use in model animal studies, which will likely enhance our understanding and interpretation of ESRD microbiome as well as CKD and related diseases.

In hemodialytic ESRD patients, the concentrations of uremic toxins in the bloodstream undergo dynamic changes influenced by factors such as diet, dialysis cycle, health status, and residual kidney function [47]. The gut microbiota appears to be an important originator of such toxins, thereby contributing to their serum toxin levels. In our previous study, we demonstrated that gut bacteria accounted for 12–44% of the variability in uremic toxin serum concentrations in ESRD patients from the Beijing cohort [21]. Here, we extend this finding to the Shanghai cohort and observe even higher percentages, with values ranging from 21.3 to 47.8% and 27.9 to 56.4% in the patients from the Shanghai and Beijing cohorts, respectively. The predictability of serum concentrations of three AAA-derived toxins by gut microbiota was validated across patient cohorts and, to some extent, even in the healthy controls. We propose that such gut microbiota-based models could potentially support clinical trials aimed at evaluating microbiome monitoring as a predictor of treatment outcomes for dialysis patients. Moreover, these models might have applications in individuals at high risk of kidney disease or CKD, serving as noninvasive indicators for assessing renal function. Furthermore, we present 67 toxin-contributing species and demonstrate that they can be used to predict toxin concentrations across cohorts. These included 14 members of Oscillospiraceae, of which 4 were *Oscillibacter* spp. and 1 was *Flavonifractor plautii*, in agreement with a recent study showing that the *Oscillibacter* was correlated significantly with serum uremic metabolites and kidney function in CKD patients [48]. Identification of these species provides a direction for further exploration of new therapeutic targets aimed at reducing uremic toxins in ESRD patients.

Metagenomic analysis of the gut microbiota of 47 additional CKD patients revealed the presence of ESRD signatures even in the CKD stage and a correlation between the abundance of ESRD-associated species and the progression of CKD. The effectiveness of ESRD microbial signatures in distinguishing CKD patients from healthy controls was also established. Previously, the alteration of gut microbiota in patients with CKD and the dependence of gut microbiota and CKD severity remained unclear due to fewer studies and their controversial results [20, 49, 50]. The microbial signatures we report provide new insights into the microbiome architecture of CKD and possibly other related kidney disorders.

## Conclusions

In conclusion, we accurately identify the gut microbiome ESRD-associated species-level taxonomic signatures and establish the taxon-specific functional connections to the disease. Importantly, these signatures are already present in CKD patients, and their abundance increases with disease progression, indicating their potential as biomarkers for CKD. We also demonstrate the effectiveness of gut species abundances in predicting serum uremic toxin concentrations across different cohorts. Our study thus depicts the gut microbiome landscape for ESRD and establishes numerous species/function-disease linkages. Although many of the hypotheses raised by this study have not been proven or disproven, we suggest that our results and resources will promote future mechanistic and therapeutic works in kidney diseases.

## Methods

### Cohorts and fecal specimen collection

#### Shanghai cohort

The Ethics Committee of Zhongshan Hospital, Fudan University, approved this study (no. B2016-112 and B2020-257), and each participant signed an informed consent agreement. Two hundred eighty-two adults (18–70 years old), including 124 ESRD patients, 47 CKD patients (12 of CKD stage 3, 4 of CKD stage 4, and 31 CKD5N), and 111 healthy controls, were recruited from Zhongshan Hospital, Fudan University (see Additional file 2: Fig. S21 for the flow diagram of recruiting). The ESRD patients were diagnosed with kidney failure under the criteria [51] of having eGFR < 15 mL/min/1.73m^2^ (calculated based on the CKD-EPI equation) or were in ongoing renal replacement therapy. All ESRD patients were receiving maintenance hemodialysis (2–3 times per week) for at least 6 months (ranging from 6 to 264 months, median 52 months). The CKD patients were diagnosed under the criteria: stage 3, eGFR < 60 mL/min/1.73m^2^; stage 4, eGFR < 30 mL/min/1.73m^2^; and stage 5, eGFR < 15 mL/min/1.73m^2^. All participants in the healthy group (screened from the inpatient physical examination population of Zhongshan Hospital) exhibited normal blood pressure, normal laboratory parameters from routine tests (blood, urine, stool, liver function, renal function, electrolytes, erythrocyte sedimentation rate, fasting blood glucose, and lipids), and normal results from chest radiography, electrocardiogram, and liver and kidney ultrasound. Exclusion criteria for all three groups were as follows: presence of chronic infection or an infection within 3 months; use of antibiotics, probiotics, prebiotics, or synbiotics within 3 months; obesity (BMI ≥ 28 kg/m^2^) or significantly underweight (BMI < 18.5 kg/m^2^); co-occurrence of significant atherosclerosis, severe liver disease, a history of gastrointestinal surgery, inflammatory bowel disease, irritable bowel syndrome, or malignant tumor; and pregnancy or lactating status.

#### Beijing cohort

The cohort characteristics, sampling, and other experimental methods were described in the previous study [21].

The fecal specimens were collected from participants, temporarily stored on dry ice, transported to the laboratory within 24 h, and stored at − 80 °C for further analysis. The peripheral blood specimens were collected at the hospital within 24 h of defecation (prior to dialysis initiation for patients). The blood was centrifuged at 3000 g for 10 min, and the serum was collected and stored at − 80 °C.

### Measurements and targeted quantification of uremic toxins

The demographic and clinical data were collected from the medical records of the hospital. Biochemical evaluations were measured using standard methods in the clinical laboratory. Biochemical evaluations were conducted using standard methods in the clinical laboratory. The serum concentrations of four uremic toxins (IS, PCS, TMAO, and PAG) were quantified using a liquid chromatography/mass spectrometry/mass spectrometry (LC–MS/MS) technology, following previously reported methods: IS [52], PCS [53], TMAO [54], and PAG [55].

### Fecal DNA extraction and whole-metagenome shotgun sequencing

For fecal samples of the Shanghai cohort and the newly-sequenced Beijing samples, total bacterial DNA was extracted from approximately 200 mg of fecal samples using the HiPure Stool DNA Kit (Magen, Guangzhou, China) and operations followed the specification. Briefly, STL buffer (1 ml) was added to 50 mg of sample in a 2-ml screw cap tubes prior to incubation at 65 °C for 10 min. After samples were put under vortex movement (15 s) and centrifugation (13,000 × g, 10 min), 600 μl of supernatants was transferred to fresh 2.0 ml tubes. Samples were then added 150 μl of PS buffer and 150 μl of absorber solution. Following a second centrifugation (13,000 × g, 5 min), the supernatants were placed in fresh 2.0 ml tubes where 700 μl of GDP buffer was added. Lastly, HiPure DNA Mini Column (Magen, Guangzhou, China) was used to absorb products and washed with sterile water. Libraries were prepared by using the NEB Next® Ultra™ DNA Library Prep Kit for Illumina (NEB, USA). Briefly, the fresh genomics DNA samples were mechanically fragmented by sonication to a size of approximately 350 bp. The DNA fragments were then subjected to end polishing, A-tailing, and ligation with the full-length adapter, followed by PCR amplification. The PCR products were purified by AMPure XP system (Beckman Coulter, Beverly, USA). Subsequently, the DNA libraries were metagenomic shotgun sequenced based on the Illumina NovaSeq platform, which generated 2 × 150 bp paired-end reads for further analysis. Initial base calling of metagenomic data was performed based on the system default parameters under the sequencing platform. The raw sequencing reads were processed for quality controls using fastp [56]. Reads with low quality (> 45 bases with quality score < 20, or > 5 ‘N’ bases), low complexity, and or adapter sequences were removed. The remaining reads were trimmed at the tails for low quality (< Q20) or ‘N’ bases. Human genomic reads were eliminated by mapping against the reference human genome (GRCh38) using Bowtie2 [57].

### Metagenomic-assembled genomes

High-quality clean reads were utilized for de novo assembly using MEGAHIT [58] with a broad range of k-mer sizes (–k-list 21,41,61,81,101,121,141). Assembled contigs (minimum length threshold 2000 bp) were binned using MetaBAT2 [59] with default parameters. Only raw bins with a total size > 200 kbp were retained for further analyses. The sequencing depth of bins was calculated by mapping the high-quality reads back to the bins with Bowtie2 [57]. Taxonomic classification of the bins was realized based on the GTDB-Tk toolkit [60], which assigns the sequences of each bin to the Genome Taxonomy Database [61]. The taxonomic name of the bins was manually adjusted to accord with traditional nomenclatures. To enhance the genomic completeness of bins, raw bins were merged within each sample if they had approximately equal sequencing depth (± 10%) and G + C content (± 2%) and shared identical taxonomic assignment at the species level.

The quality of MAGs was evaluated using CheckM [62] with the lineage_wf workflow. The definition of high- and medium-quality MAGs was based on the minimum information about metagenome-assembled genome (MIMAG) standards [30] (high: > 90% completeness and < 5% contamination, presence of 5S, 16S and 23S rRNA genes, and at least 18 tRNAs; medium: ≥ 70% completeness and < 10% contamination). And the quality score was defined as “QS = completeness – 5 × contamination,” following Parks et al. [63] All high- and medium-quality MAGs with quality score > 60 were clustered at the nucleotide level by dRep [64], for which the MAGs sharing > 95% nucleotide identity were considered redundant. Within each cluster, the MAG with the highest QS was selected as the representative MAG, referred to as a “species.” Finally, the high-quality sequencing reads of each sample were mapped to the nonredundant MAG catalog (consisting of 1303 MAGs) using Bowtie2 [57] to generate the relative abundance of these MAGs. For the taxonomic profiles at the phylum, class, order, family, and genera levels, we summed the relative abundance of MAGs from the same taxon to yield the abundance of that taxon.

The phylogenetic tree of MAGs was built using PhyloPhlAn [65] and visualized in iTOL [66].

### Gene-centric functional analysis of metagenomes

We conducted an overall functional analysis of the fecal metagenomes using a gene-centric strategy developed in our previous studies [26, 67]. A microbial gene catalog was constructed based on the metagenomic assemblies from the samples of all individuals. Briefly, ab initio microbial genes were identified from assembled contigs (minimum length threshold 500 bp) using Prodigal [68] at the metagenome mode. The predicted genes were clustered by CD-HIT [69] at the nucleotide level similarity > 95% and sequence overlap > 90%, resulting in a nonredundant gene catalog comprising 11,971,559 genes. The relative abundance of genes in each sample was determined by mapping the sequencing reads into the gene catalog. The KEGG database was used for the functional annotation of genes using Blast KOALA [70]. Each protein was assigned a KEGG orthologue (KO) on the basis of the best-hit gene in the database, with an *e*-value < 1e − 10 and covering > 50% of the protein length. KOs were assigned into pathways or modules based on the KEGG website (https://www.kegg.jp). The abundance of a functional category (KO, pathway, or module) was calculated from the summation of the relative abundance of its corresponding genes.

Antibiotic resistance genes were identified using ABRicate (https://github.com/tseemann/abricate) and Mustard [71]. ABRicate searched the databases, including the NCBI Bacterial Antimicrobial Resistance Reference Gene Database, CARD [72], ARG-ANNOT [73], and ResFinder [74], for predicting ARGs. Amino acid sequences of the gene catalog were aligned against the databases using DIAMOND [75] (*e*-value < 1e − 10) and assigned to ARGs by the highest-scoring annotated hit with > 80% similarity that covered > 80% of the length of the query protein.

### Functional annotation and analyses of MAGs

Prediction of protein-coding genes, rRNAs, and tRNAs of MAGs was carried out using Prodigal [68] (single genome mode), RNAmmer [76], and tRNAscan-SE [77], respectively. KEGG and ARG annotations of the protein-coding genes were realized following the aforementioned method. Annotation of CAZymes was performed by aligning the protein sequence of each MAG against the CAZy database using DIAMOND [75] with an *e*-value < 1e − 10 and covering > 50% of the protein length. Polysaccharide utilization capacity analysis of MAGs was performed using PULpy [78], and the candidate substrates of polysaccharide utilization loci were determined following Fechner-Peach et al. [33] Analysis of biosynthesis capacity of SCFAs, SBAs, and uremic toxins of MAGs was realized following our previously developed method [21]. Briefly, we used the presence of key synthetases to denote the biosynthesis capacity of such molecules for each MAG: acetate synthase (acetyl-CoA decarbonylase/synthase), propionate synthase I (lactoyl-CoA dehydratase), propionate synthase II (propionaldehyde dehydrogenase), butyrate synthase I (butyryl-CoA:acetate CoA-transferase), butyrate synthase II (butyrate kinase), bile salt hydrolase, 7α/β-dehydroxylation enzymes, hydroxysteroid dehydrogenase, tryptophanase, tyrosine phenol-lyase, 4-hydroxyphenylacetate decarboxylase (p-Cresol synthase), phenylacetaldehyde dehydrogenase (phenylacetate synthase), phenyllactate dehydratase (*fldBC*), and choline trimethylamine-lyase.

### Gut microbiota-based regression models

The gut microbiota-based regression model was utilized for estimating the predictability of the serum concentrations of uremic toxins based on the relative abundance profiles of the gut species. A detailed description of the methodology for this regression model has been detailed in our previous study [21]. Briefly, for each uremic toxin (i.e., IS, PAG, PCS, and TMAO), we initially trained a random forest model based on the relative abundances of all gut species and used a leave-one-out cross-validation to predict the serum concentration of this toxin based on the model. The coefficient of determination (*R*^2^) between the predicted values and the actual values represented the explainability of the gut species to this toxin. Then, to achieve higher explainability, we ranked all the species based on their importance (measured by the increase mean square error, IncMSE) and sequentially selected a certain number of species for training a new model and evaluating its performance. This iterative process allowed us to obtain the model with the highest performance (estimated by *R*^2^) and a corresponding set of training species. The highest performance reflected the extent to which the gut microbiota could explain the variability of the specific toxin.

### Statistical analyses

Statistical analyses were conducted using the R 4.0.1 platform. For comparison analyses, the *P*-values were calculated using the Wilcoxon rank-sum test, Kruskal–Wallis rank-sum test, or Fisher’s exact test, depending on the specific scenario. The combined *P*-value of two independent cohorts was calculated based on Fisher’s method [79]. The *q* value was used to evaluate the false discovery rate (FDR) for the correction of multiple comparisons and was calculated based on the R *fdrtool* package [80]. The following criteria were employed to identify ESRD-associated taxonomic and functional signatures from two independent cohorts: (1) *q* < 0.2 and fold change > 1.2 in each cohort with coherent enrichment in patients or controls and (2) combined *q* < 0.05.

Principal coordinates analysis (PCoA) and distance-based redundancy analysis (dbRDA) were performed with the R *vegan* package, utilizing Bray–Curtis dissimilarly as the metric, and the results were visualized via the R *ade4* package. Permutational multivariate analysis of variance (PERMANOVA, *adonis* analysis) was realized with the R *vegan* package, and the *adonis P*-value was generated based on 1000 permutations. Mantel test was performed using the R *ade4* package.

### Supplementary Information

**Additional file 1: Table S1.** Summary on statistics of the host properties and clinical parameters from the Shanghai and Beijing cohorts. **Table S2.** Summary of deep whole-metagenomic shotgun sequencing data production of this study. **Table S3.** Detailed information of data production, assembly, and MAG reconstruction of 715 samples. **Table S4.** Detailed information of 1303 non-redundant microbial species reconstructed from the fecal metagenomes of this study. **Table S5.** Detailed information of the 100 most discriminant species in the random forest regression models of the Shanghai and Beijing cohorts. **Table S6.** Detailed information of 353 ESRD-associated species. **Table S7.** Detailed information of 1279 ESRD-associated KOs. **Table S8.** Detailed information of 103 ESRD-associated KEGG functional modules. **Table S9.** Detailed information of 3,009 ARGs identified in this study. **Table S10.** Detailed information of 730 KOs that significantly differed in occurrence frequency between ESRD-enriched and HC-enriched Firmicutes species. **Table S11.** Detailed information of 40 modules that significantly differed in integrity between ESRD-enriched and HC-enriched Firmicutes species. **Table S12.** Detailed information of 67 gut species with the highest contribution of uremic toxin levels in serum. **Table S13.** Abundances and statistical test of 353 ESRD-associated species among the CKD patients vs. healthy controls.

**Additional file 2: Figure S1.** Genome-wide phylogeny of 1,303 gut microbial species. **Figure S2.** Species boundary and reads mappability of the 1,303 gut species in this study. **Figure S3.** Comparison of microbial diversity between ESRD patients and healthy controls. **Figure S4.** Distribution of samples in the five primary PCs. **Figure S5.** Random forest models for discriminating ESRD patients and healthy controls based on gut species profile. **Figure S6.** Identification of ESRD-associated species from two independent cohorts. **Figure S7.** Comparison of functional profiles between ESRD patients and healthy controls. **Figure S8.** Identification of ESRD-associated functional signatures from two independent cohorts. **Figure S9.** Comparison of antibiotic resistance genes between ESRD patients and healthy controls. **Figure S10.** Effect of the bacterial phylogeny on the functional composition. **Figure S11.** Occurrence of several types of enzymes in Firmicutes species. **Figure S12.** Modules differing in completeness between ESRD-enriched and HC-enriched non-Firmicutes species. **Figure S13.** Analysis of polysaccharide utilization for ESRD-associated Bacteroidetes species. **Figure S14.** Relationship between individuals’ dietary pattern and their metagenomic polysaccharide utilization and Prevotellaceae/Muribaculaceae level. **Figure S15.** Presence of several important enzymes in the ESRD-associated species. **Figure S16.** Distribution of the key synthetases involved in the biosynthesis of uremic toxins in fecal metagenomes. **Figure S17.** Predicting the concentrations of toxins in ESRD patients, based on the gut microbial species. **Figure S18.** Predicting the concentrations of toxins in healthy subjects based on the gut microbial species. **Figure S19.** Heatmap presented below illustrates the relative abundance variations of ESRD-associated species in healthy controls, as well as patients with CKD stage 3-4, CKD5N, and ESRD. **Figure S20.** Random forest models for discriminating CKD patients from the healthy controls based on gut species profile of ESRD patients. **Figure S21.** Flow diagram of recruitment of individuals in the Shanghai cohort.

**Additional file 3.** Review history.

## Data Availability

The statistical scripts are available at https://github.com/lish2/esrd_microbiome [81]. The raw metagenomic sequencing dataset acquired in this study have been deposited to the European Nucleotide Archive under the accession code PRJEB65297 (https://www.ebi.ac.uk/ena/browser/view/PRJEB65297) [82]. The MAG sequences acquired in this study are available at https://github.com/yexianingyue/gut-microbiome-of-ESRD [83]. The authors declare that all other data and materials supporting the findings of the study are available in the paper and supplementary materials.
