# Supplementary material for: Metagenome-wide analysis uncovers gut microbial signatures and implicates taxon-specific functions in end-stage renal disease
Source: Genome Biol. 2023 Oct 12;24:226. doi: 10.1186/s13059-023-03056-y (PMC10571392; doi:10.1186/s13059-023-03056-y)
Supplement: Supplementary file 2 — Additional file 2: Figure S1. Genome-wide phylogeny of 1,303 gut microbial species. Figure S2. Species boundary and reads mappability of the 1,303 gut species in this study. Figure S3. Comparison of microbial diversity between ESRD patients and healthy controls. Figure S4. Distribution of samples in the five primary PCs. Figure S5. Random forest models for discriminating ESRD patients and healthy controls based on gut species profile. Figure S6. Identification of ESRD-associated species from two independent cohorts. Figure S7. Comparison of functional profiles between ESRD patients and healthy controls. Figure S8. Identification of ESRD-associated functional signatures from two independent cohorts. Figure S9. Comparison of antibiotic resistance genes between ESRD patients and healthy controls. Figure S10. Effect of the bacterial phylogeny on the functional composition. Figure S11. Occurrence of several types of enzymes in Firmicutes species. Figure S12. Modules differing in completeness between ESRD-enriched and HC-enriched non-Firmicutes species. Figure S13. Analysis of polysaccharide utilization for ESRD-associated Bacteroidetes species. Figure S14. Relationship between individuals’ dietary pattern and their metagenomic polysaccharide utilization and Prevotellaceae/Muribaculaceae level. Figure S15. Presence of several important enzymes in the ESRD-associated species. Figure S16. Distribution of the key synthetases involved in the biosynthesis of uremic toxins in fecal metagenomes. Figure S17. Predicting the concentrations of toxins in ESRD patients, based on the gut microbial species. Figure S18. Predicting the concentrations of toxins in healthy subjects based on the gut microbial species. Figure S19. Heatmap presented below illustrates the relative abundance variations of ESRD-associated species in healthy controls, as well as patients with CKD stage 3-4, CKD5N, and ESRD. Figure S20. Random forest models for discriminating CKD patients from the healthy controls [file 13059_2023_3056_MOESM2_ESM.docx]

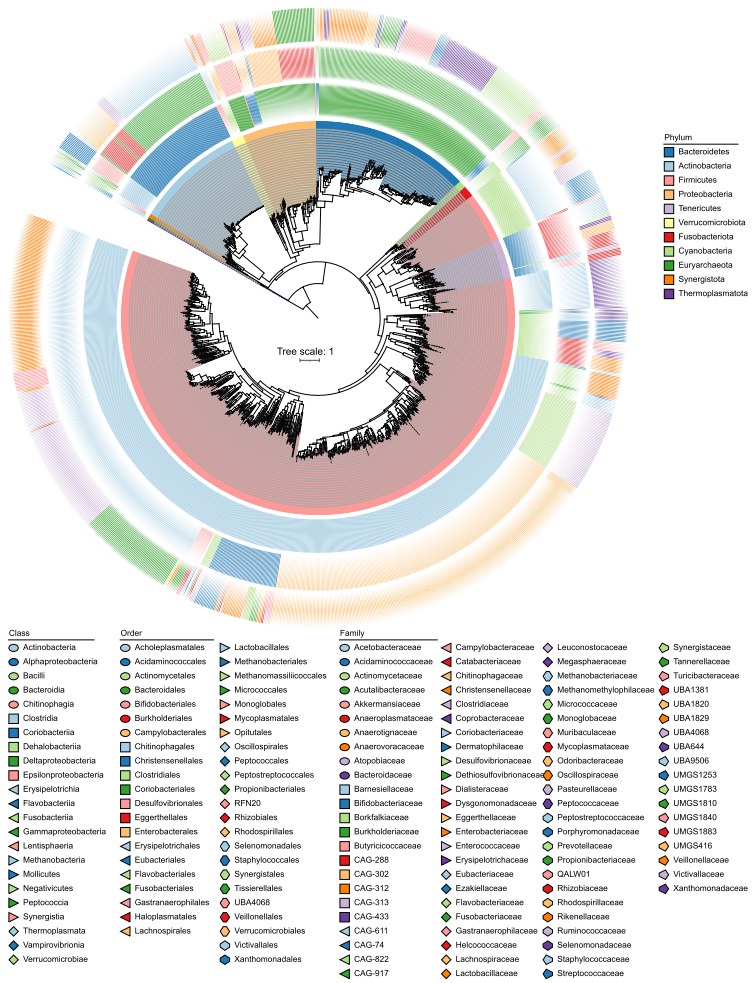


**Figure S1.** Genome-wide phylogeny of 1,303 gut microbial species. Innermost circle shows the phylogenetic tree of 1,303 species reconstructing from fecal metagenomes in this study. Colors of the phylogenetic tree represent the phylum level assignment of each species. The outer three circles (from inside to outside) represent the class, order, and family levels assignment of each species. Detailed information of 1,303 species is shown in Additional file 1: Table S4.


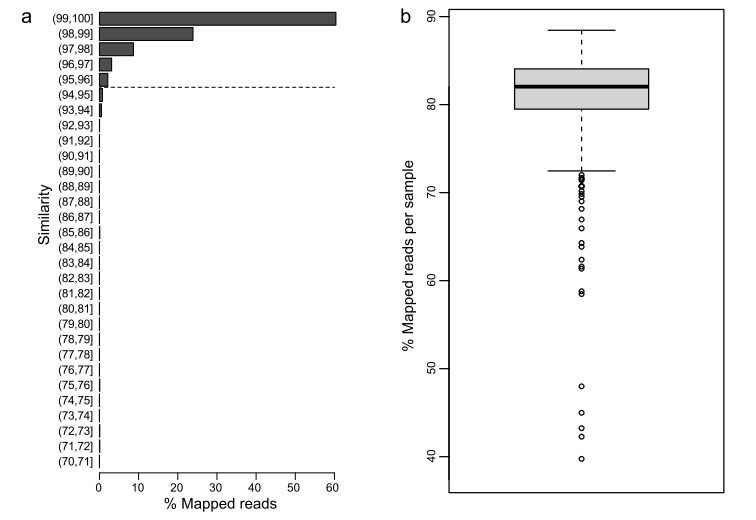


**Figure S2.** Species boundary and reads mappability of the 1,303 gut species in this study. a, Barplot showing the distribution of nucleotide identity of metagenomic reads that had mapped at a unique site of the species from the catalog. Only a minor fraction of the reads had identity below 95%, indicating that this threshold separates different species well. b, Boxplot showing the proportion of reads for each sample mapped onto the species catalog. Boxes represent the interquartile range between the first and third quartiles and the median (internal line). Whiskers denote the lowest and highest values within 1.5 times the range of the first and third quartiles, respectively; dots represent outlier samples beyond the whiskers.


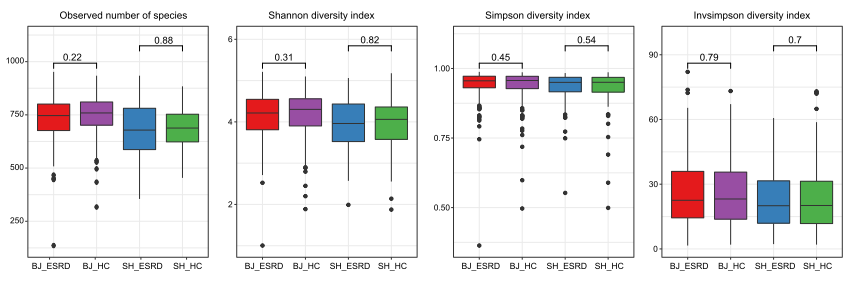


**Figure S3.** Comparison of microbial diversity between ESRD patients and healthy controls. Boxplot showing the distribution of four alpha diversity indexes, including the observed number of species, Shannon diversity index, and Simpson and Invsimpson diversity indexes, among groups. Transverse lines above the boxes show the p-values of Student’s t test.


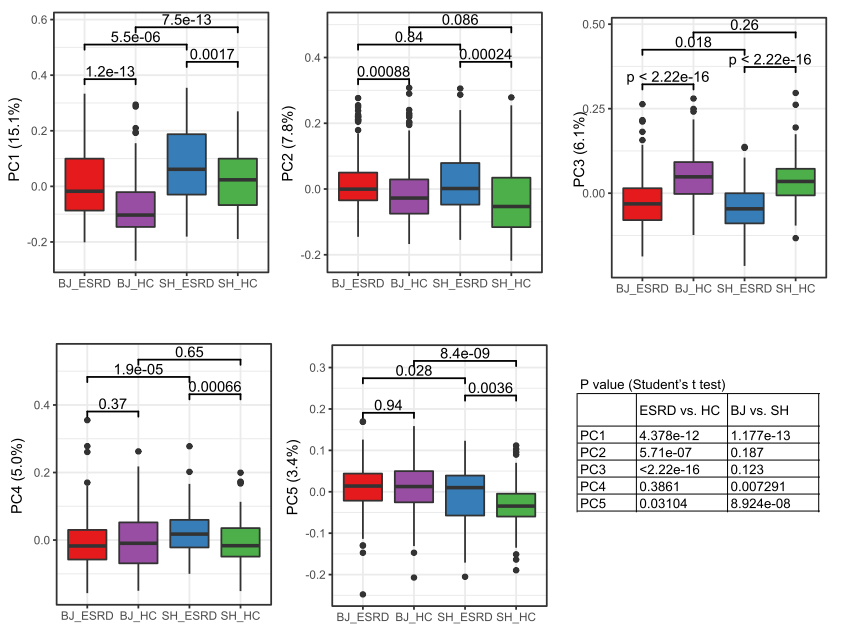


**Figure S4.** Distribution of samples in the five primary PCs. The top five PCs are tested for correlations with ESRD vs. HC and Beijing vs. Shanghai cohort, and the p-values are shown.


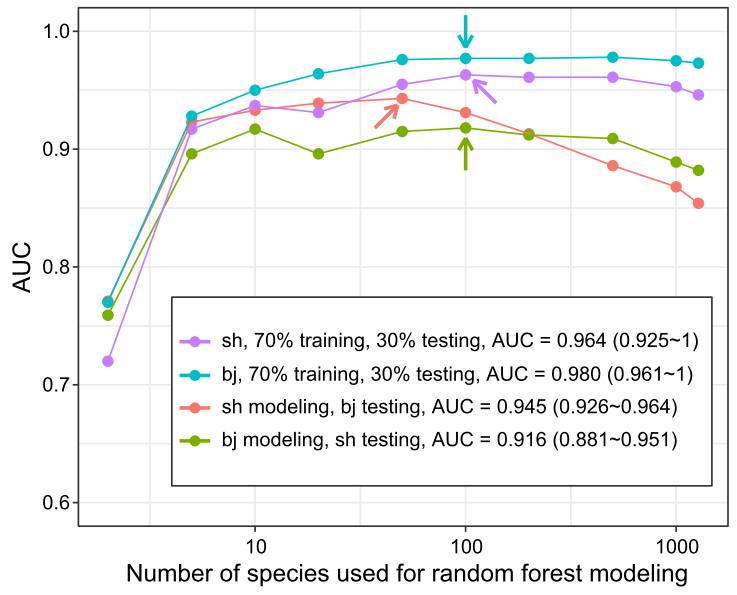


**Figure S5.** Random forest models for discriminating ESRD patients and healthy controls based on gut species profile. Classification performance of a random forest model assessed by area under the receiver-operating characteristic curve (AUC). The performance was explored for different numbers of species, ordered in importance. The arrows indicated the number of species at the best performance.


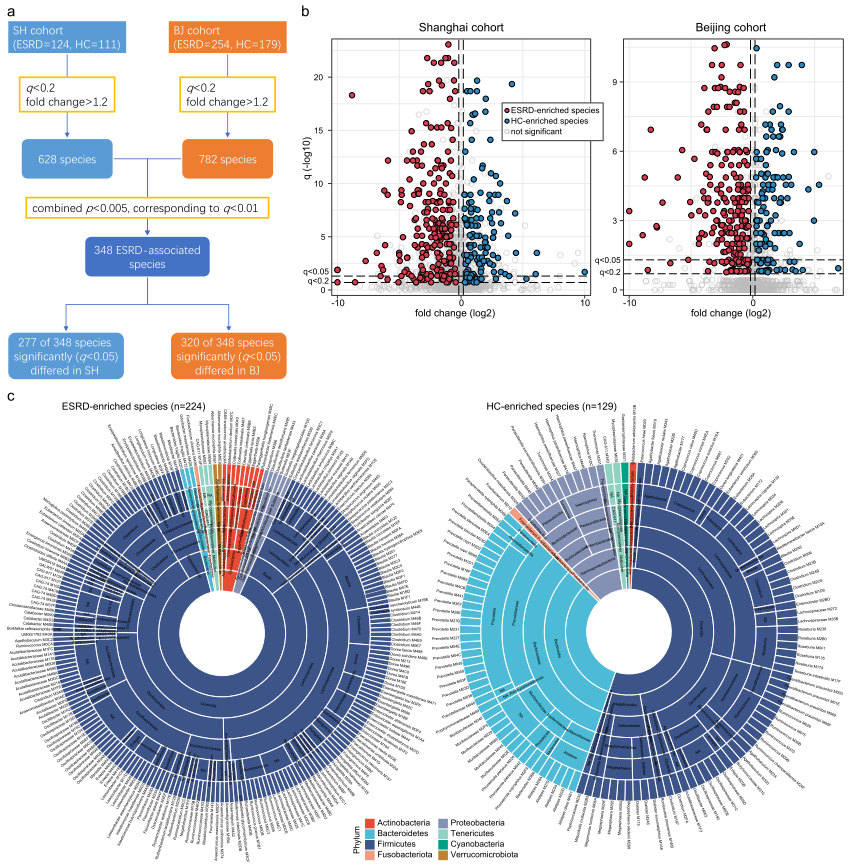


**Figure S6.** Identification of ESRD-associated species from two independent cohorts. a, Overall schematic to identify the ESRD-associated species from two cohorts. b, Volcano plot showing the fold change vs. q-values for all species in the Shanghai (left panel) and Beijing (right panel) cohorts. The X-axis shows the fold change ratio (log2 transformed) of a species abundance in ESRD patients compared with healthy controls (fold change <0, ESRD-enriched; fold change >0, HC-enriched), and the two dotted lines indicate the fold change ratio -1.2 and 1.2. The Y-axis shows the q-value (-log10 transformed) of a species, and the two dotted lines indicate q-value <0.05 (upper) and <0.02 (lower). The ESRD-associated species with a consistent trend in two cohorts are shown in red (ESRD enriched) and blue (control enriched) circles. c, Taxonomic distribution of the ESRD-enriched (left panel) and HC-enriched species (right panel).


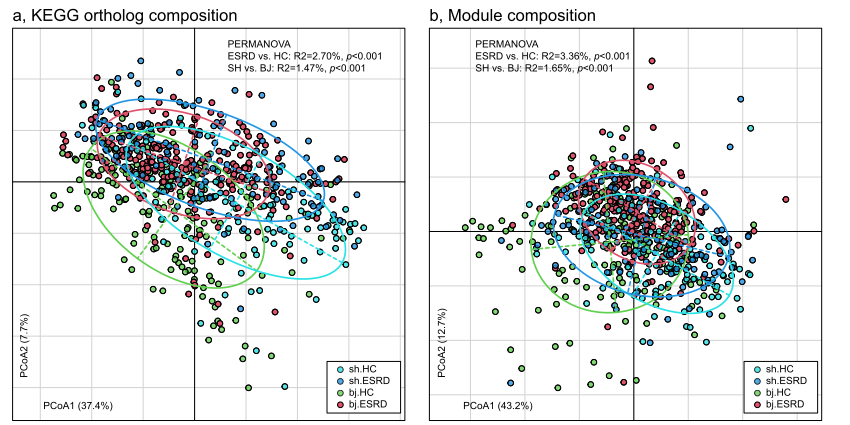


**Figure S7.** Comparison of functional profiles between ESRD patients and healthy controls. PCoA analysis showing the comparison of patients and controls at the KEGG ortholog composition (a) and module composition (b). Samples are shown at the first two principal coordinates (PCoA1 and PCoA2). Ellipsoids represent a 95% confidence interval surrounding each group. The PERMANOVA results are shown at the top of the PCoA plots.


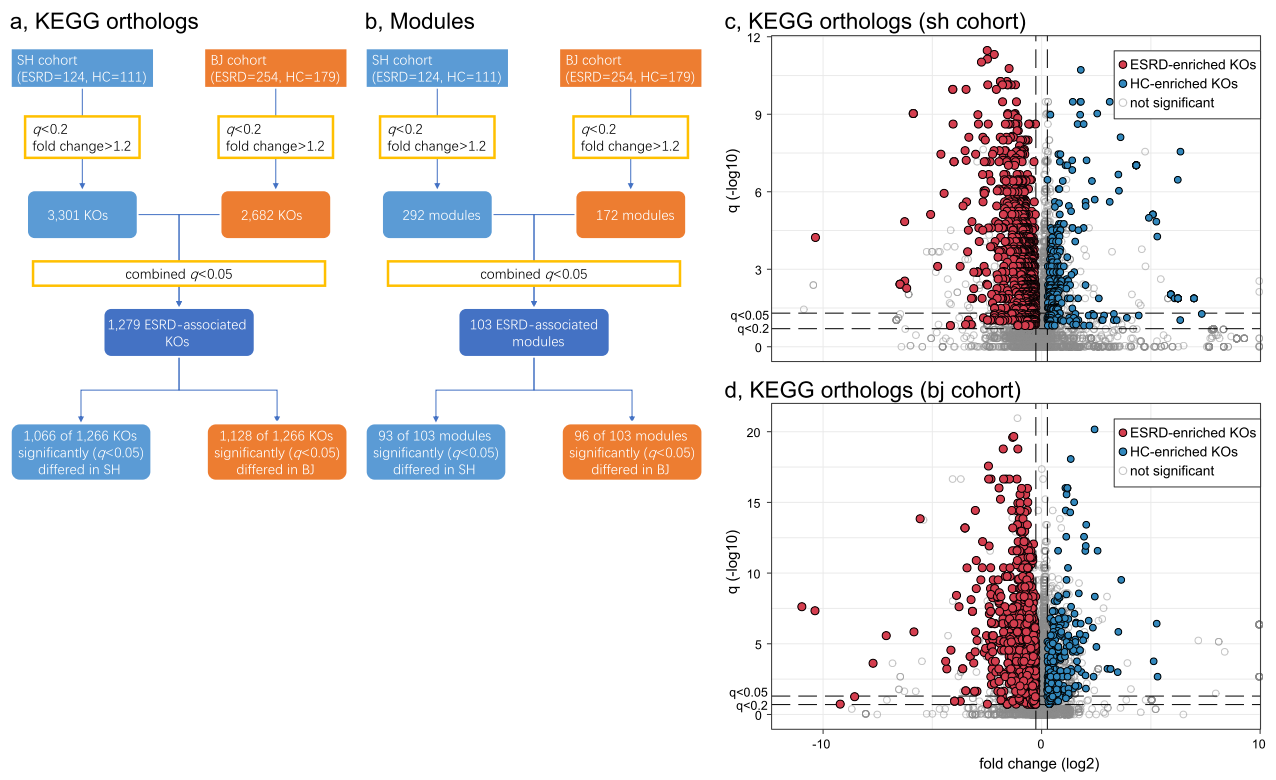


**Figure S8.** Identification of ESRD-associated functional signatures from two independent cohorts. a-b, Overall schematic to identify the ESRD-associated KEGG orthologs (a) and modules (b) from two cohorts. c-d, Volcano plot showing the fold change vs. q-values for all KEGG orthologs in the Shanghai (c) and Beijing (d) cohorts. The X-axis shows the fold change ratio (log2 transformed) of a species abundance in ESRD patients compared with healthy controls (fold change <0, ESRD-enriched; fold change >0, HC-enriched), and the two dotted lines indicate the fold change ratio -1.2 and 1.2. The Y-axis shows the q-value (-log10 transformed) of a species, and the two dotted lines indicate q-value <0.05 (upper) and <0.2 (lower). The ESRD-associated species with a consistent trend in two cohorts are shown in red (ESRD enriched) and blue (control enriched) circles.


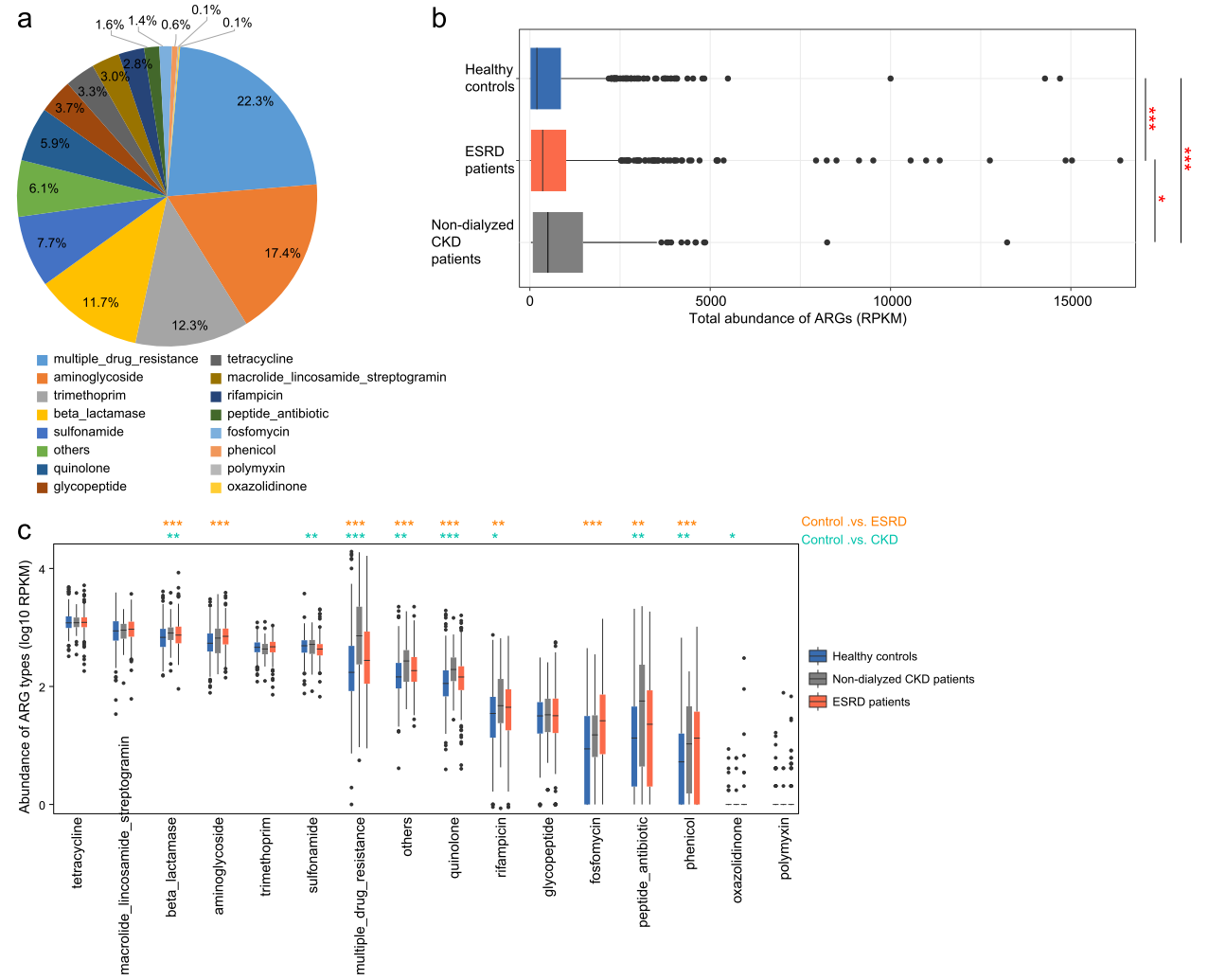


**Figure S9.** Comparison of antibiotic resistance genes between ESRD patients and healthy controls. a, Pie plot showing the categories of 1,614 ARGs identified by this study. b-c, Boxplot showing the comparison of total abundance (b) and abundance of each ARG category (c) between two cohorts. Student’s t test: *, *q*<0.05; **, *q*<0.01; ***, *q*<0.001. RPKM, reads per kilobase per million mapped reads.


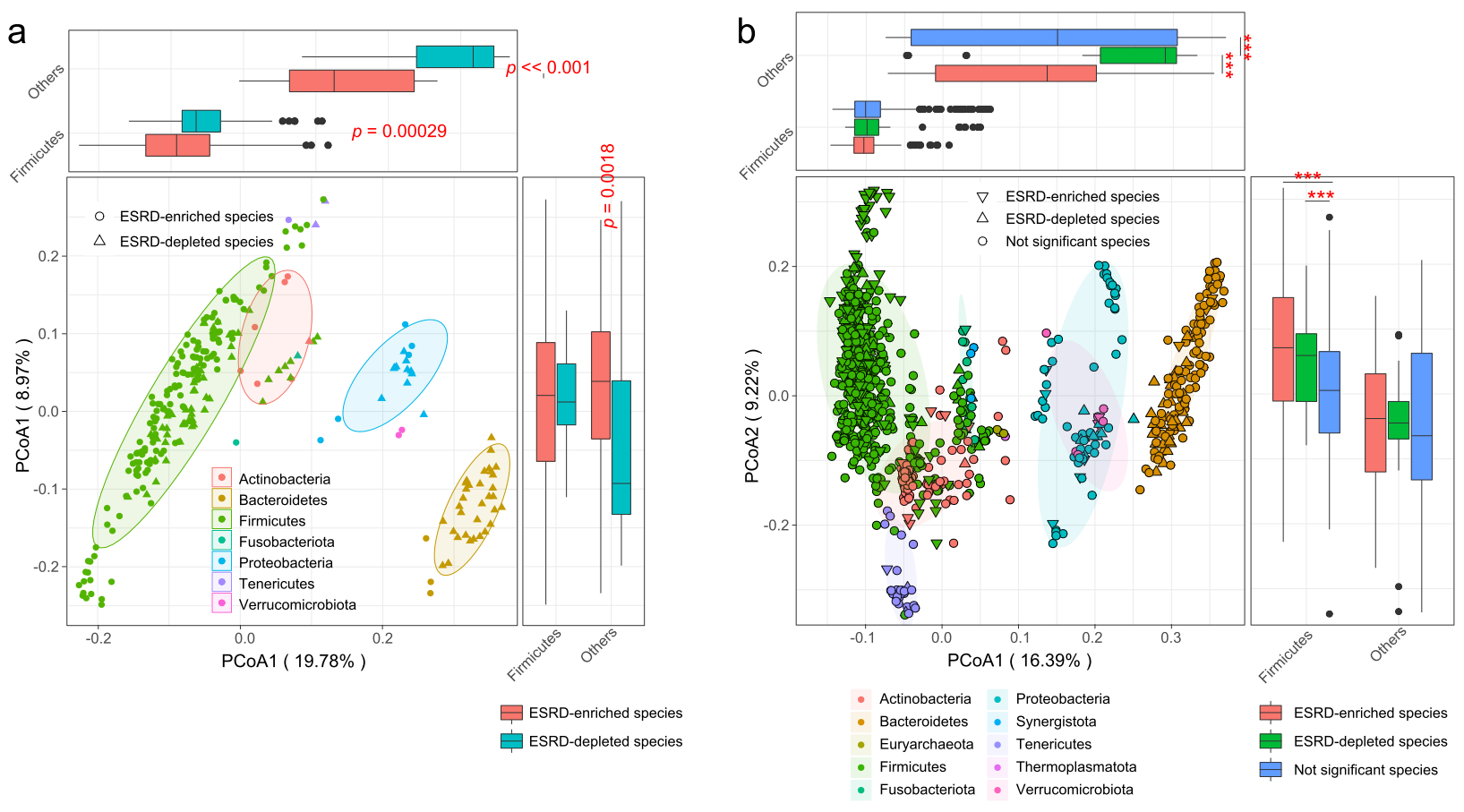


**Figure S10.** Effect of the bacterial phylogeny on the functional composition. PCoA analyses of KEGG ortholog profile of ESRD-associated species (a) and all bacterial species (b) are shown. The first two principal coordinates and the ratio of variance contributed are shown. Box-and-whisker plots display first and third quartiles, and whiskers are from each quartile to the minimum or maximum. For ESRD-associated species (a), the result reveals that the overall functional profiles of species are primarily drivered by their phylum-level classification (effect size =22.1%, p<0.001) but rarely determined by their enrichment in ESRD patients (effect size =3.3%, p<0.001). This finding suggested that the ESRD status didn’t significantly connect to the fundamental functions of microbes (e.g., functions that relate to bacterial lifestyle) and it only potentially correlated with some specific functions of some taxa. Therefore, in further analysis, we performed the functional comparisons within the members of Firmicutes and non-Firmicutes respectively to minimize the impact of the phylogeny. Student’s t test: *, *q*<0.05; **, *q*<0.01; ***, *q*<0.001.


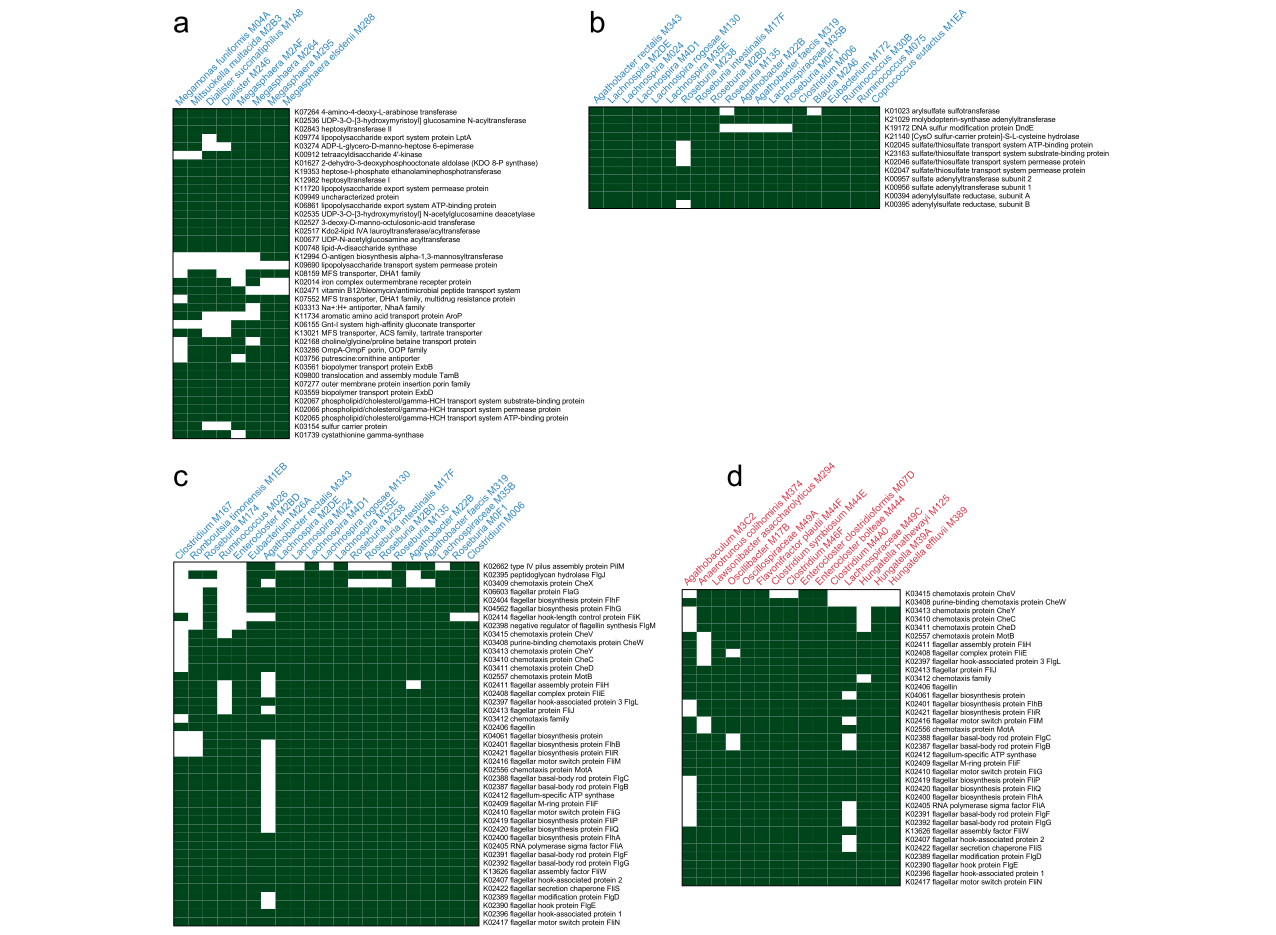


**Figure S11.** Occurrence of several types of enzymes in Firmicutes species. Heatmap showing the occurrence of enzymes involved in Negativicutes-specific transporters and LPS biosynthesis and export (a), sulfur and sulfate metabolism (b), and bacterial motility and chemotaxis (mostly flagellar biosynthesis) (c-d, for HC-enriched and ESRD-enriched species, respectively). Colors of the species name indicate its enrichment: blue, HC-enriched; red, ESRD-enriched.


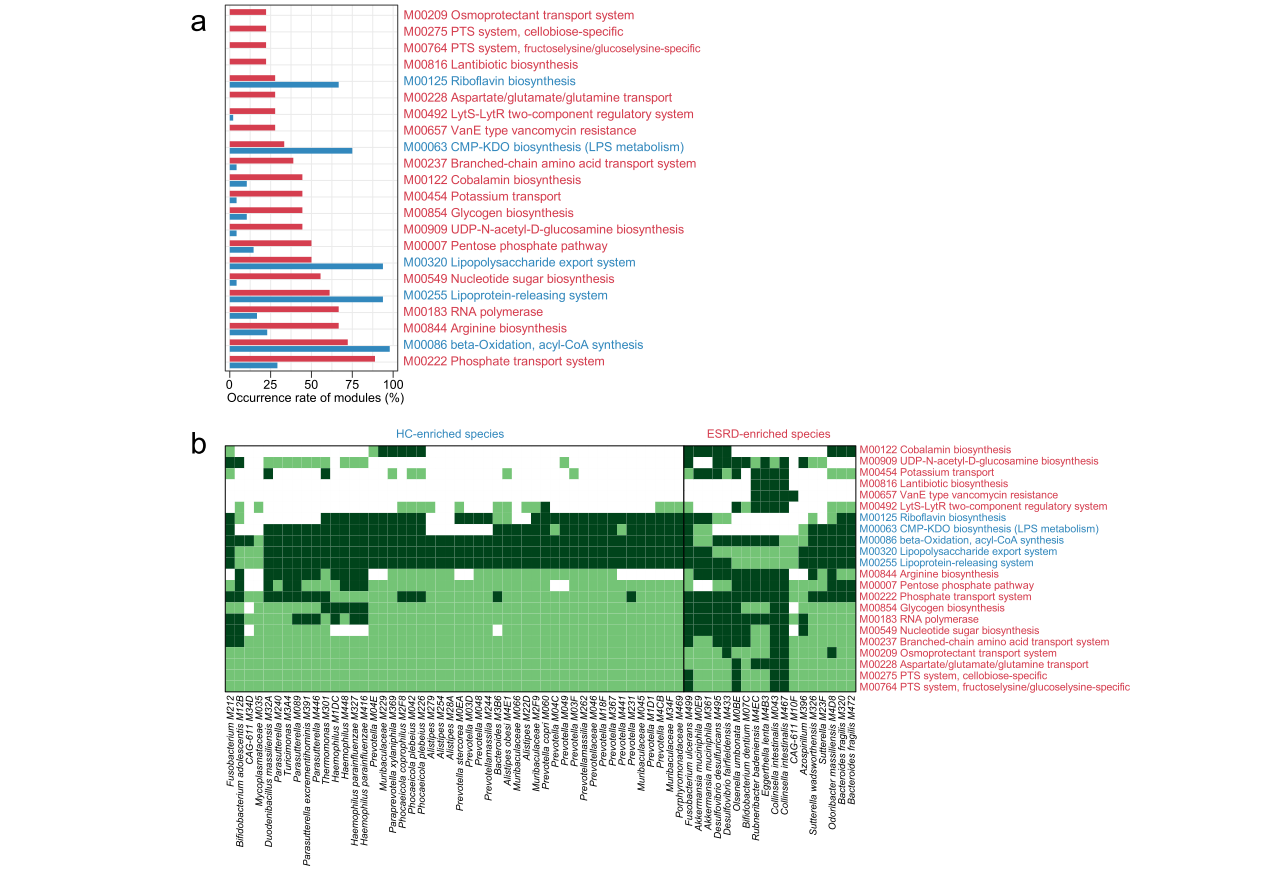


**Figure S12.** Modules differing in completeness between ESRD-enriched and HC-enriched non-Firmicutes species. a, Bar plots showing the occurrence rate of KEGG modules in the ESRD-associated non-Firmicutes species. 20 KEGG modules that differed in integrity between ESRD-enriched and HC-enriched non-Firmicutes species are shown. The colored bars show the percentage of species that contained complete KEGG modules: red, module present in ESRD-enriched species; blue, module present in HC-enriched species. b, Heatmap showing the completeness ratio of KEGG metabolic modules for ESRD-associated non-Firmicutes species. The color shows the completeness ratio of each species: dark green, complete; light green, largely complete (only one enzyme not found). The modules that are more frequent in ESRD-enriched and HC-enriched species are colored in red and blue, respectively.


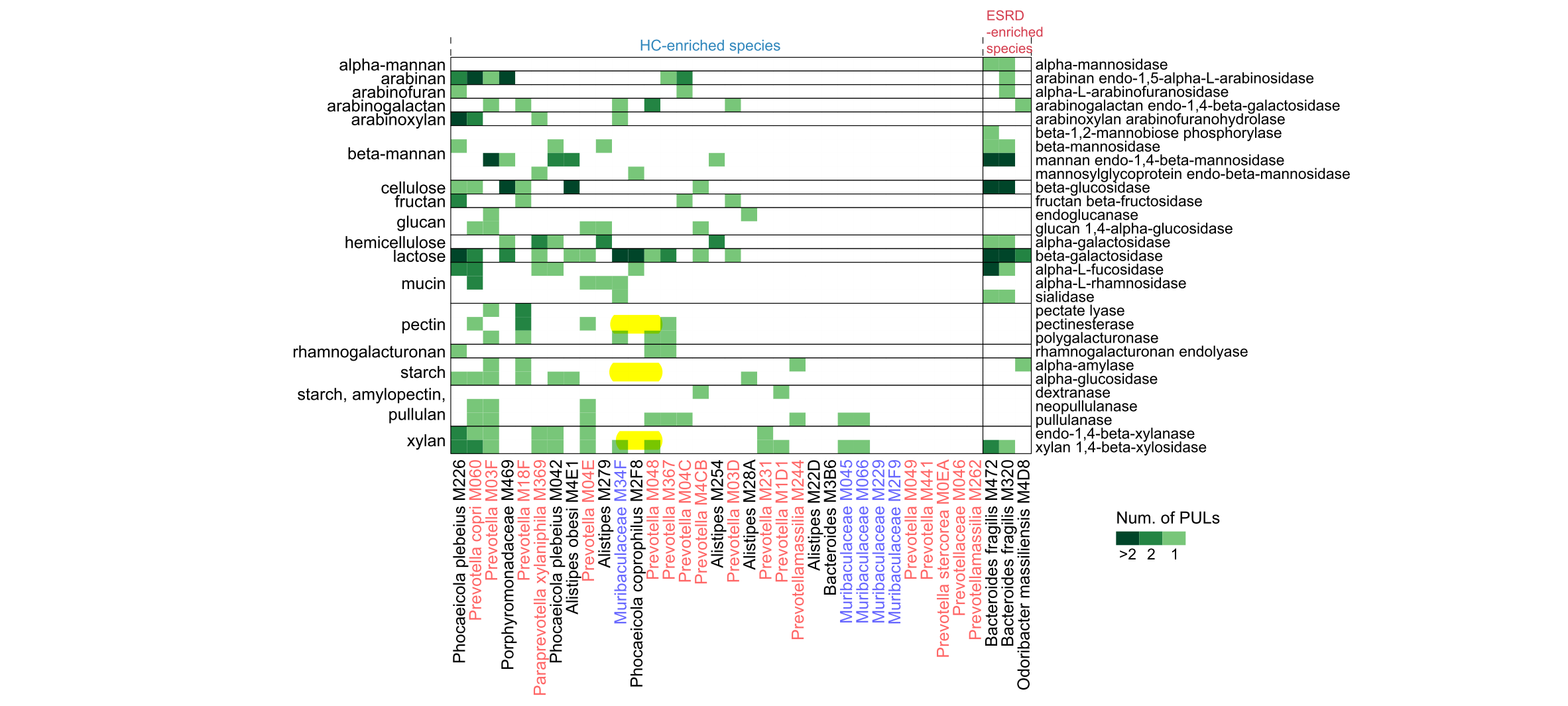


**Figure S13.** Analysis of polysaccharide utilization for ESRD-associated Bacteroidetes species. Polysaccharide utilization loci (PULs) analysis of the ESRD-associated Bacteroidetes species. PULs were categorized by the presence of annotated genes with polysaccharide catabolism activity.


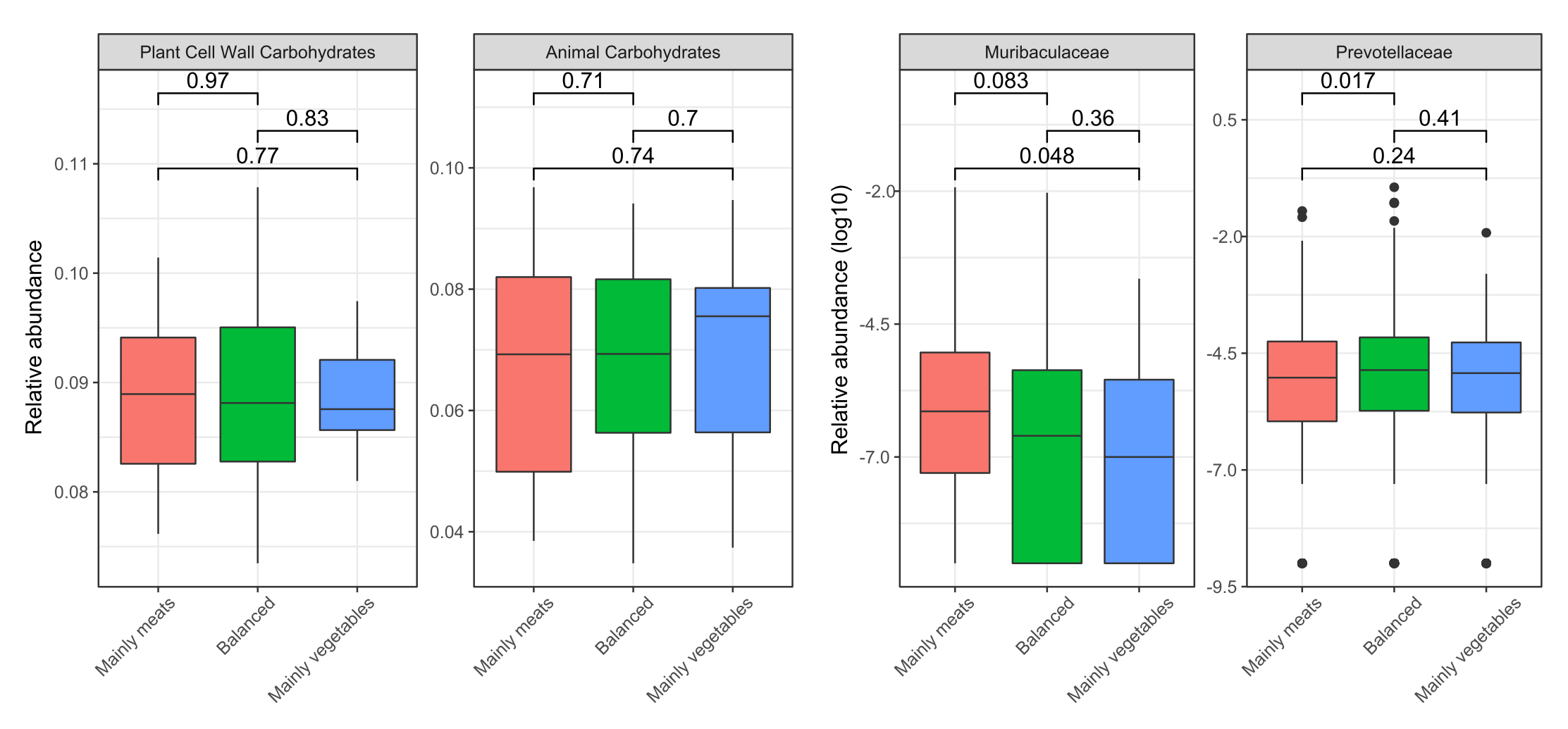


**Figure S14.** Relationship between individuals’ dietary pattern and their metagenomic polysaccharide utilization and Prevotellaceae/Muribaculaceae level. Boxplots show the distribution of relative abundance of polysaccharide utilization, Prevotellaceae and Muribaculaceae in individuals with different dietary patterns, including mainly meats, mainly vegetables, and balanced individuals.


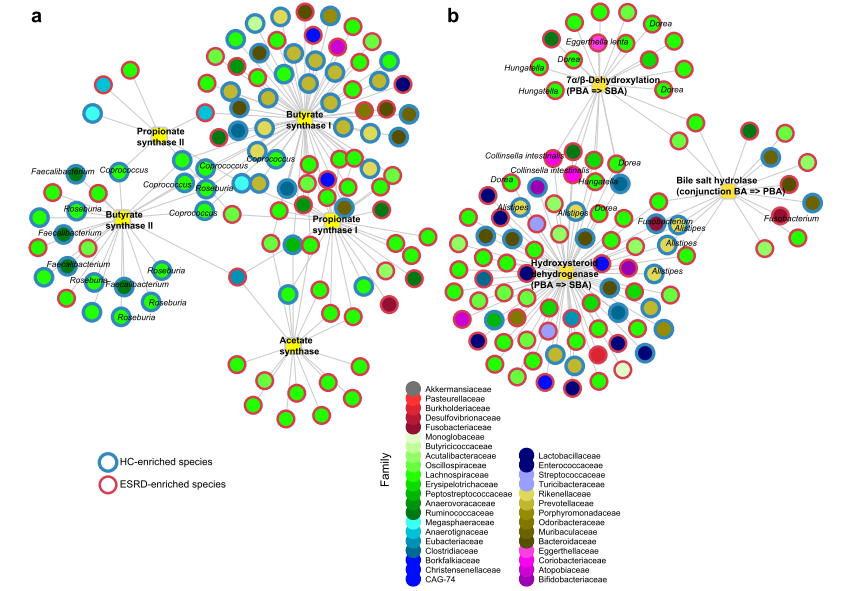


**Figure S15.** Presence of several important enzymes in the ESRD-associated species. Network view of the occurrence of enzymes involved in the synthesis of SCFAs (left panel) and bile acids (right panel) in the ESRD-associated species. Squares represent the enzymes, and the surrounding connected circles represent the species. The filled color of species denotes their family-level taxonomic assignment, and the boundary color denotes their enrichment in ESRD patients and healthy controls.


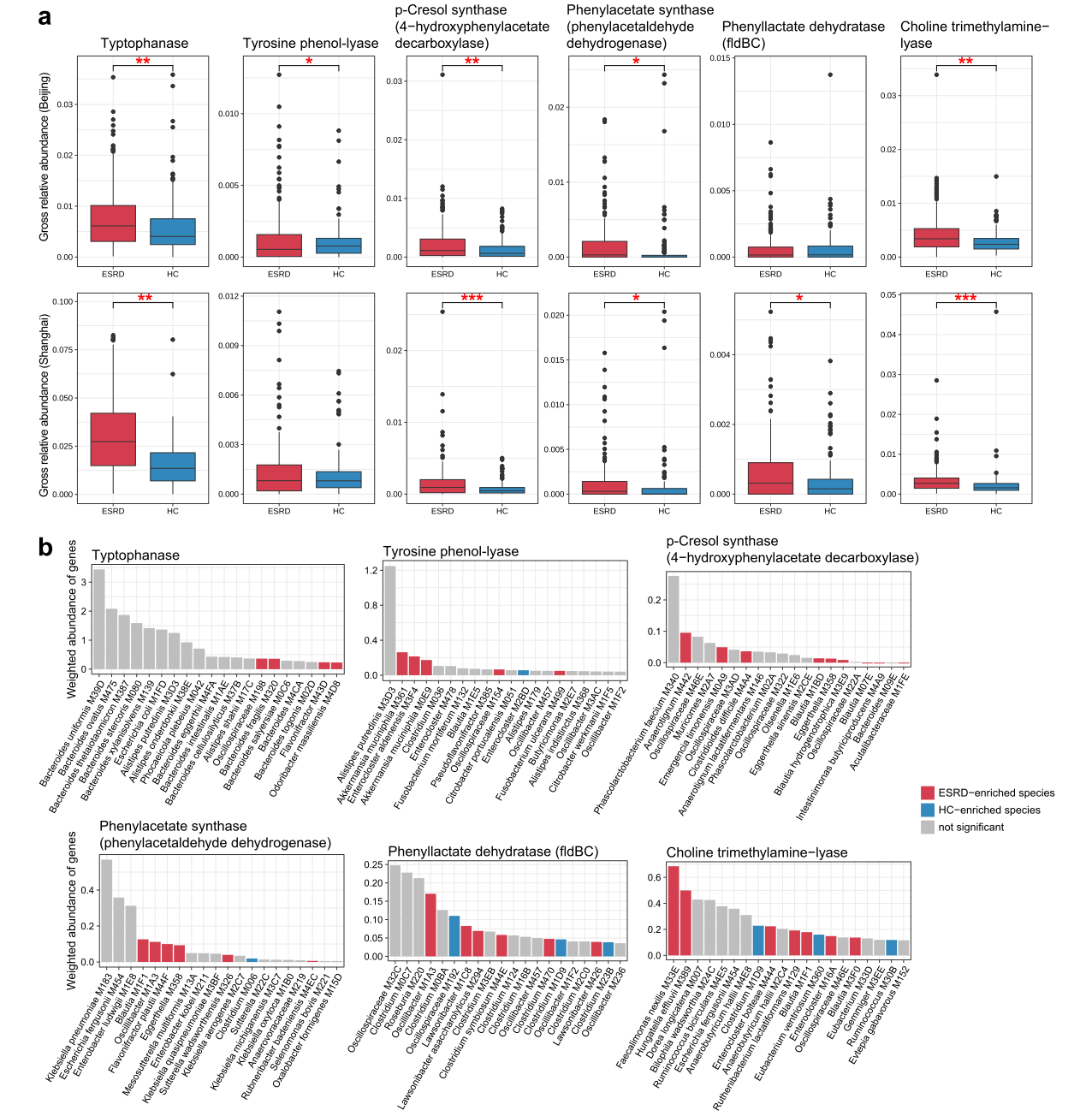


**Figure S16.** Distribution of the key synthetases involved in the biosynthesis of uremic toxins in fecal metagenomes. a, Relative abundance of key synthetase-encoding genes in ESRD patients and healthy controls. Statistical significance, as determined by the Wilcoxon rank-sum test between the patients and controls, is denoted by asterisks: *, *q*<0.05; **, *q*<0.01; ***, *q*<0.001. b, The mean weighted abundance of toxin-encoding genes in different gut species across fecal metagenomes. Each bar plot presents the top 20 species with the highest weighted abundance. The species are colored according to their enrichment in ESRD patients and healthy controls.


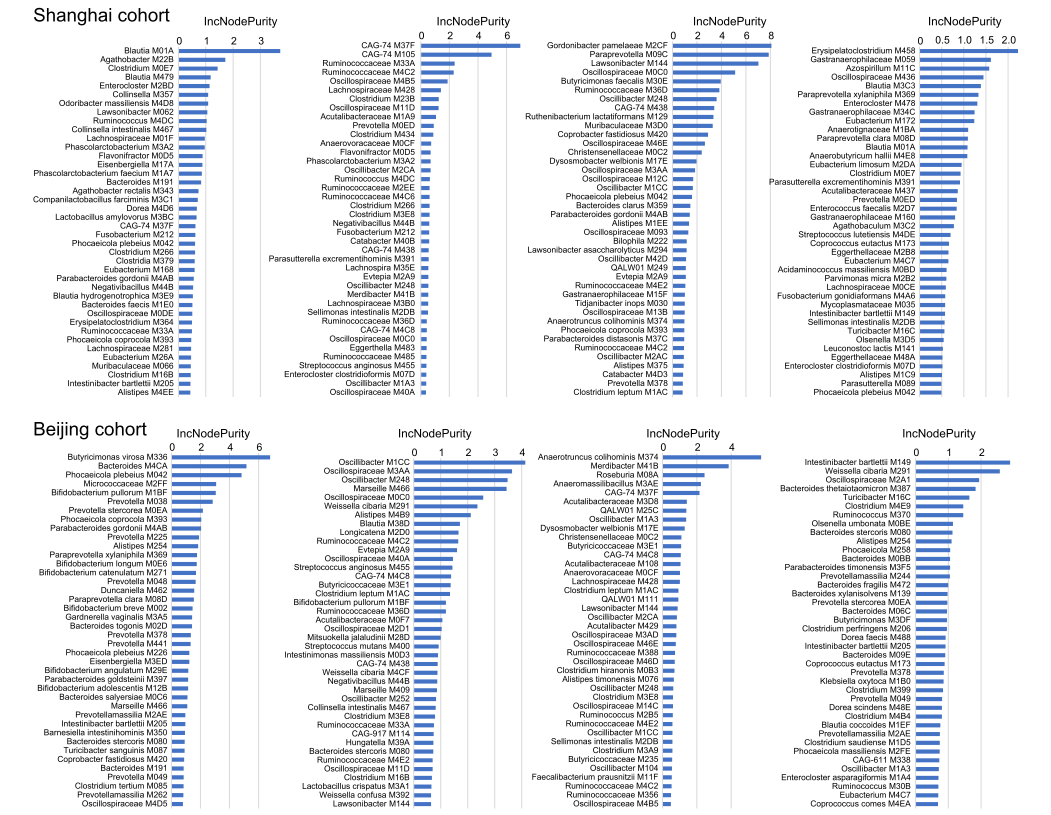


**Figure S17.** Predicting the concentrations of toxins in ESRD patients, based on the gut microbial species. Barplots show the 40 most discriminant species in the models for each toxin. a, models trained based on the Beijing individuals. b, models trained based on the Shanghai individuals. The bar lengths indicate the importance of the variable. IncNodePurity, increase in node purity.


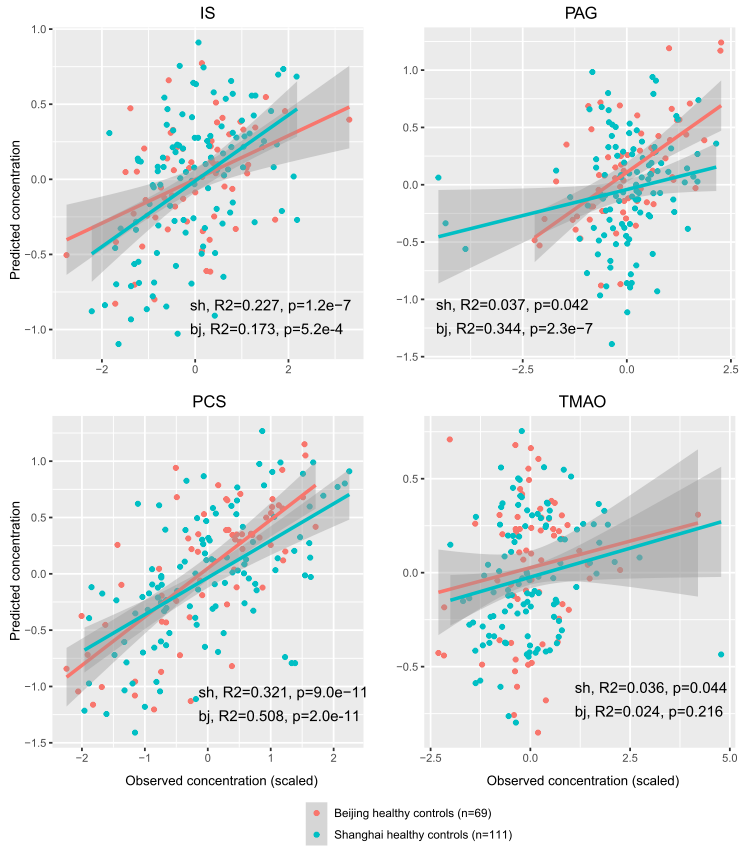


**Figure S18.** Predicting the concentrations of toxins in healthy subjects based on the gut microbial species. For each toxin, the random forest regression model was trained based on the abundance of 67 species. The X-axis and Y-axis show the predicted concentration and scaled measured concentration for each toxin. Smooth curves are formed using the geom_smooth function with the default parameters in the R ggplot2 package.


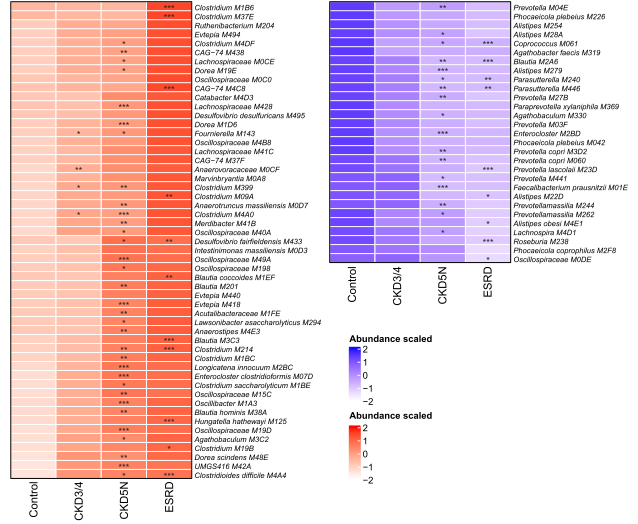


**Figure S19.** Heatmap presented below illustrates the relative abundance variations of ESRD-associated species in healthy controls, as well as patients with CKD stage 3-4, CKD5N, and ESRD. The left panel displays the abundances of 53 ESRD-enriched species, while the right panel showcases the abundances of 29 HC-enriched species, both exhibiting a consistent positive or negative trend in relation to disease severity. Statistical significance, as determined by the Wilcoxon rank-sum test between the patients and controls, is denoted by asterisks: *, *q*<0.05; **, *q*<0.01; ***, *q*<0.001.


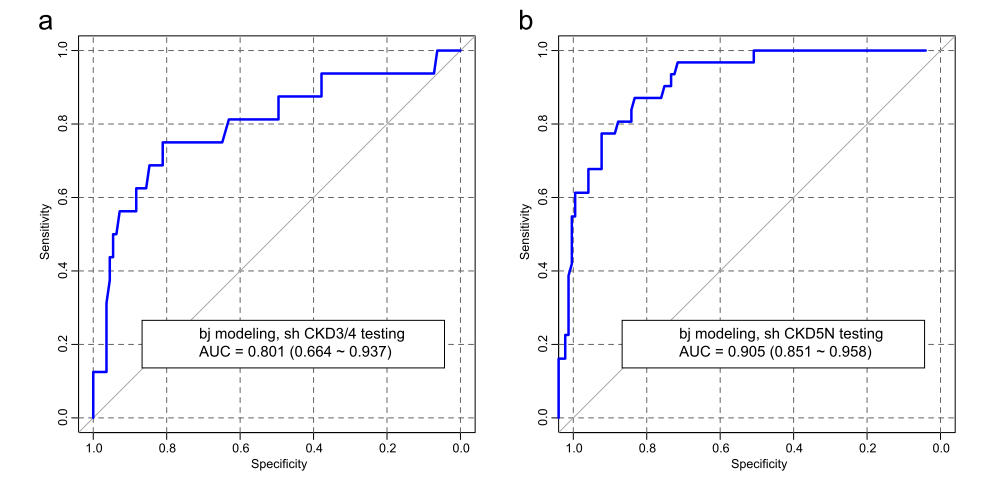


**Figure S20.** Random forest models for discriminating CKD patients from the healthy controls based on gut species profile of ESRD patients. The performance of models trained from the gut microbiota of the Beijing individuals was evaluated on the Shanghai individuals for classifying CKD3/4 (a) and CKD5N (b) from the healthy controls. Classification performance of a random forest model was assessed by area under the receiver-operating characteristic curve (AUC). The AUC values and 95% confidence intervals (CIs) are shown.


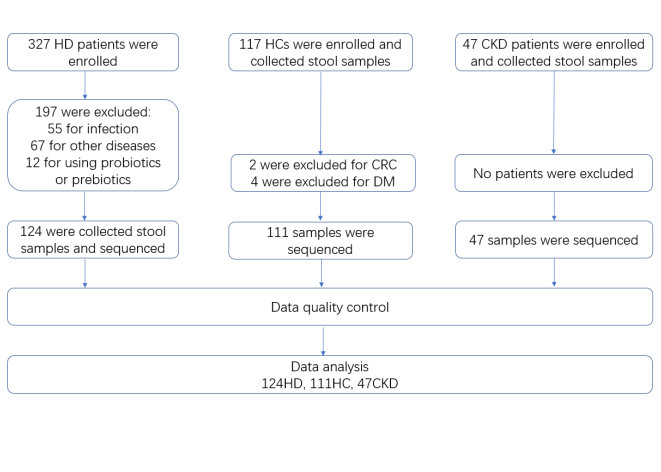


**Figure S21.** Flow diagram of recruitment of individuals in the Shanghai cohort.
