# Supplementary material for: Metagenome-wide analysis uncovers gut microbial signatures and implicates taxon-specific functions in end-stage renal disease
Source: Genome Biol. 2023 Oct 12;24:226. doi: 10.1186/s13059-023-03056-y (PMC10571392; doi:10.1186/s13059-023-03056-y)
Supplement: Supplementary file 3 — Additional file 3. Review history. [file 13059_2023_3056_MOESM3_ESM.docx]

Review History

**First round of review**

**Reviewer 1**

**Are you able to assess all statistics in the manuscript, including the appropriateness of statistical tests used?** No, I do not feel adequately qualified to assess the statistics.

**Comments to author:**

The manuscript "metagenome-wide analysis uncovers gut microbial signatures and implicates taxon-specific functions in end stage renal disease" by Zhang et al. reported a comprehensive microbial analysis on two independent cohorts based on the metagenomic data. The authors demonstrated several findings: 1. Consistency with previous studies on depletion of Prevotella, Faecalibacterium and Coprococcus, as well as enrichment in Streptococcus, Fusobacterium, and Desulfovirionaceae. 2. Species in these genera were reported.3. Function prediction based on metagenomics. 4. Links between predicted functions, uremic toxins and bacterial species.

Major concerns:

1. Based on the analysis, the authors proposed several important hypotheses. However, no mechanistic studies to prove or disprove the proposed hypothesis.

2. More details are required for random forest machine learning. What information was used for training? Any pre-analysis (e.g. LEfSe, correlation) was done on the microbiota data for the training?

3. Regarding this conclusion "the Bacteroidaceae species (containing 4 ESRD-enriched and 2 HC-enriched species, Fig1d) were more likely to utilize animal glycans,……." I don't believe the referred Figure 1d, 3e, S16a and S16b support this conclusion. Authors may clarify this statement.

4. In the section "Gut bacteria affect serum uremic toxin level". More details for "the gut microbiota-based regression models" would be beneficial. How was this generated?

Minor concerns:

1. Why PC2 (7.8%) and PC3 (6.1%) were used to present Figure 1a? Figure S4 showed a better contribution by PC1 (15.1%).

2. The first letters of some bacterial names were not capitalized.

3. Several articles have shown that modification of gut microbiota and metabolite butyrate attenuates renal disease. PMID: 36164984, 31366236. These would help with the discussion.

**Reviewer 2**

**Are you able to assess all statistics in the manuscript, including the appropriateness of statistical tests used?** Yes, and I have assessed the statistics in my report.

**Comments to author:**

In this manuscript, the authors studied the gut microbial (GM) characteristics in patients with end-stage renal disease (ESRD). They applied shotgun metagenomic sequencing, assembled reads and analyzed the gut microbial data on MAGs and gene levels. They reported distinct gut microbial features in ESRD compared to non-ESRD controls, such as the disease-enriched MAGs (Blautia spp., Dorea spp., and Eggerthellaceae) and the and multiple taxon-specific functions which might potentially contribute and thus predict the serum uremic toxin concentrations in patients. They also validated several ESRD-related microbial features in another cohort (also from the same group) including ESRD patients, non-dialyzed chronic kidney disease (CKD) patients and healthy controls.

The main concern is the novelty of this study when compared to their previous one. The group has published their first work on the ESRD-related gut microbial features in Gut (2019) (https://gut.bmj.com/content/69/12/2131.long). They applied shotgun sequencing, used assembly-based strategy, and reported the enrichment of multiple Firmicutes species in ESRD (e.g., Eggerthella lenta), and these contributed a lot to the toxin production. In addition, the capacity of multiple Firmicutes members for phenol- and p-cresol-producing have been demonstrated in numerous papers (ref, 10.1093/femsec/fiy125). However, the authors did not provide detailed information on the functional potentials on toxin-producing genes. They only used association analysis to report relationships between ESRD-enriched MAGs and toxin levels. They used their previous data (named as Beijing cohort) in combined the newly added Shanghai cohort to assembly MAGs and reanalyze their data on this topic. Most of their findings are descriptive and lack of a deep discussion. Also, a lot of information presented in their figures were not mentioned in the text. The second concern is the lack of rigor in the statistical methods and related descriptions (e.g., lack of sample number /statistical methods/ P & Q-values in most of the figure legends). (e.g., lack of a robust criterion for the identification of ESRD-related microbial species/functions. Another issue is too much information (a total 24 additional figures) but few of them were well described or discussed. I suggested that the authors should carefully review their manuscript and eliminate any unnecessary results or redundancy. This will help to streamline the paper and make it more focused. It's important for the authors to be transparent about the limitations and potential sources of variability in their data, and to provide a clear rationale for their findings. They need to address below issues before the paper can be accepted.

Part 1 The gut microbiome stratifies ESRD patients from healthy controls

P7, line 52-52, which data showed 0.68%? Lack of figure or table?

"In ESRD patients, the underlying kidney disease (e.g., glomerulonephritis or diabetic kidney disease) explained only 0.68% (p=0.23) of the gut microbial variations."

P7, Figure 1 and Fig. S5. Both Shanghai model and Beijing model showed extremally high AUCs (>0.9) by using ~100 RF selected MAGs, even in testing set. Could the author provide the importance for selected MAGs in their RF models? Which MAGs have the highest importance values (e.g., GINI)? How many MAGs were consistently selected for two models and how about the consistency of the top features in two models?

Part 2 Microbial species related to ESRD

In the method (Page 19), the authors stated that "The following criteria were used for identifying ESRD-associated taxonomic and functional signatures from two independent cohorts: 1) q<0.2 and fold change >1.2 in each cohort with coherent enrichment in patients or controls, and 2) combined p<0.005." (Fig. S6).

First, it is wired that why the author used the criterion FD>1.2. Because their Volcano plot (Fig.S6) already showed that most of their MAGs including the not significant ones showed a FD>1.2 between two groups. Think they could just remove this criterion.

Second, why they used combined P<0.005 (Q<0.01) to define the ESRD-related features?

Why not a combined Q<0.05 ? Or combined the features with a Q<0.05 in each independent cohort, should this be more common? Their data already showed that in the same 348 MAGs, 277 and 320 MAGs were significantly differed in SH and BJ cohorts, respectively (Q<0.05 in each study). Or a stricter but simpler criterion could be the shared MAGs features pass the Q<0.05 in each cohort. PS, it is not clear how authors calculate the q values in the combined dataset? Based on 1,303 MAGs? Or based on other datasets?

Think the authors should improve their methods to ensure a robust definition of ESRD-related MAGs. Please revised the results accordingly (P8, line9-line18). "Some 348 differentially abundant species between the ESRD patients and healthy controls were identified using the combined significance level in the two cohorts (combined Wilcoxon rank-sum test p<0.005, corresponding to q<0.01; Additional file 2: Fig. S6); 221 were more abundant in patients and 127 in controls. All species remained significant upon adjustment for sex, age, and BMI, and, a majority (73.6%) had a significant (q<0.05) and coherent enrichment in both cohorts (Additional file 1: Table S5)."

P8. Line21-Line22. "12 of 18 proteobacteria were enriched in controls". The authors did not discuss this result. It is weird that most (12/18) of proteobacteria were enriched in the controls. As discussed in a previous review (doi: 10.1093/advances/nmz028), most of the human studies have consistently found an enrichment of Gammaproteobacteria/ Enterobacteriaceae in the ESRD/CKD patients (pre or post dialysis) and these group are well known phenol producers.

P8，Line38-Line 43 "Notably, some closely related species exhibited opposite trends between patients and controls (Additional file 2: Fig. S8), suggesting that the difference in functions they encode may be related to ESRD." Lack of details in results.

Part 3 Microbial functions related to ESRD

P8，Line 59-Line61. Please define the significance cutoff for functional KO and modules. (Figure S10). Also please keep an eye on the above suggestions on ESRD-related species. It is weird as the combined P<0.005 equals to different q values in different datasets (between species, KO and modules).

P9, Line1-Line5. "Modules that were most highly ESRD-biased involved antimicrobial resistance and bacterial toxicity, phosphotransferase systems for monosaccharide (e.g., fructose and mannose) uptake, secondary metabolite biosynthesis, and transport systems (Fig. 2c, d; Additional file 1: Table S6-7)". It is very hard for readers to get the conclusion based on the heatmap based on Kos (Fig. 2c). Also, if we read the heatmap very carefully, we could find the ESRD patients have two distinct patterns in these functional modules. As shown below, only half of the patients have enrichment (in red) while the rest seemed to have comparable abundances as healthy controls? Did the authors have any results on this part? Think it is a very important topic and worth a try to improve the novelty of the work.

P9, Line 7- Line9. "The ESRD-depleted functions included inositol phosphate metabolism and ubiquinone and pyridoxal biosynthesis, among others." What is "among others"?

P9, Line 28-Line32. Line 33-Line38. "The total abundance of ARGs increased 39.4% in ESRD patients as compared with the healthy controls (p<0.001; Additional file 2: Fig. S11a,b)." "The most prominent changes were observed in genes associated with multidrug resistance, beta-lactamase, aminoglycoside, and quinolone resistance (Additional file 2: Fig. S11c). These observations suggest that patients may have been more frequently exposed to antibiotics than the controls in the past, possibly due to treatment of dialysis-associated infections [29]." What did the author mean?

39.4% of the 1,614 ARGs were increased in ESRD or the RPKM was 39.4% higher in ESRD than HC? Also, few information is provided by the AGR analysis. Think the author could delete this part and focused on their main findings.

P10, Line1-Line4. "These KOs were mostly within several categories; for example, enzymes involved in antimicrobial resistance and degradation of aromatic compounds were more widespread in ESRD-enriched species, whereas those associated with cofactor and vitamin metabolism and AAA biosynthesis were mostly present in HC-enriched species (Fig. 3a)." Please clarify which cofactor or vitamin metabolism-related KOs are enriched in HC-enriched species? (VB2? VB7? ??) No useful information could observe in the heatmap.

It is reasonable to compare the functional configuration of the ESRD-enriched Firmicutes and HC-enriched controls (a, b). However, I do not think the comparisons between ESRD- and HC-enriched non-Firmicutes make sense. It is also hard for readers to understand the related Fig S12 (no P values, no details for the effect size). Because these MAGs enriched in different groups are from different phylogenetic levels (even at the phylum level). For instance, in Fig. 3c, we could find that multiple "Specific transporters of ESRD-enriched species" were detected in HC-enriched species. And most HC-enriched were gram-negative species and therefore harbored LPS-related functions. I do not think these comparisons could provide valuable information for clinical researcher in this field.

P10, Line 9-Line 13. The author stated that "Proteins associated with bacterial motility (flagellar biosynthesis) were mainly encoded by the HC-enriched Lachnospira, Roseburia, and Agathobacter (Additional file 2: Fig. S13a, b). However, in FigS13 a, b, we could observe that species enriched in both patients and HC encoded almost the same potential functions involved in bacterial motility.

P10. Line 50-Line 60. For another instance (Fig. 3e), we could hardly conclude that "Genomic annotation of carbohydrate-active enzymes (CAZymes) revealed that, consistent with previous studies [32, 33], the Bacteroidaceae species were more likely to utilize animal glycans, whereas the HC-enriched Prevotellaceae and Muribaculaceae members were likely to use plant glycans such as xylan, starch, and pectin" based on the PCoA analysis (Fig. 3e). Instead, both the figure 3e and the related Fig. S16 suggested similar CAZyme profiles between the NS (not significant) species and the HC-enriched species. These data more likely suggested different CAZyme profiles between different phylogenetic members (e.g., Bacteroidetes vs. Prevotellaceae and Muribaculaceae), but not suggested the different CAZyme profiles between disease-enriched species and HC-enriched ones. The author should be very careful when interpret these results. Otherwise, I would suggest they delete un-related findings/ figures, revise their analysis and focuse on more important topics. (e.g., why they do not present the results from Fig.S17 in the main figure)

P11. Fig. 3f. Please provide methods for correlations between MAGs and functions on legends. Please define what is "more frequent" or "significantly more frequent" in this section.

Part 4 Gut bacteria affect serum uremic toxin levels

P12. Fig4.

It is very interesting that the Oscillospiraceae MAGs (half were disease-enriched) showed very close and positive relationships with the uremic toxin levels. However, they did not go deeper to investigate the potential enzymes in the Oscillospiraceae MAGs. Since they already have the KO profiles, I would suggest they provide more data on this part. The authors repeatly stated that "The 67 gut species with the highest contribution to serum toxin levels. However, they did not provide any functional data to support whether these MAGs were previously toxin-producers or whether they harbored functional genes involved in toxin production? PS, It is also very interesting for readers whether the disease-enriched, toxin-related MAGs overlapped with those having the highest distinguish power between HC and patients in the RF models?

In Fig20, very different MAGs were selected as the top species for the prediction of toxins in two cohorts. Why? Did the authors have any explanation?

P12. "In addition, Mantel test analysis revealed that the composition of these toxin-contributed species significantly correlates with the individuals' clinical parameters such as high-sensitivity C-reactive protein (hsCRP) and estimated glomerular filtration rate (eGFR) (Fig. 4c), suggesting that they potentially play critical roles in human physiological health." In my opinion, these correlations between so called "toxin-contributed species" and clinical parameters could largely contributed by the highly positive relationships between the toxin levels and these clinical parameters (eGFR and hsCRP). I don't think this observation could suggest their "potentially play critical roles in human physiological health." Try and see if you could repeat it after adjustment for the toxin levels.

Part 5 Gut microbiota in non-dialyzed CKD patients

P12. I would not suggest they use the FD>1.2 to report the tendency between case and controls as I stated before most of NS species also showed FD>1.2 between case and controls. Try to use effect size to report the tendency.

The author combined all the ESRD-enriched species and showed a gradually increased abundances from HC to ESRD and stated that "the abundance of the ESRD-enriched and depleted species was significantly correlated with the severity of CKD (Fig. 5b), supporting the hypothesis that ESRD-associated bacteria might be related to CKD progression and the development of ESRD."

It could be more valuable for reads and doctors to provide more information to show which MAGs are more related to disease severity but not the overall abundances of the 200+ MAGs.

Discussion:

Authors should carefully summarize the key findings and their implications in the context of the current state of knowledge in the field (try not to overstate their findings). Also, please put more words on their novelty and/or significance of their findings.

Main concern 1: P13. Line 55-Line 65. P14. Line1-Line 5.

"For instance, we found that the proteins associated with flagellar biosynthesis were mainly encoded by the HC-enriched members of Lachnospira, Roseburia, and Agathobacter and the ESRD-enriched Hungatella spp. These species encode flagellin, which has been previously demonstrated to have immunomodulatory properties associated with multiple inflammatory diseases [40, 41]. In particular, Roseburia-derived flagellin is a negative regulator of gut inflammation [42] and shows protection effects in barrier functions [43]. These findings suggest that the flagellar/flagellin biosynthesis capacity may have important physiological significance in ESRD; we suggest that this research direction is worthy of further exploration."

As I mentioned above, most of the KOs involved in flagellar/flagellin biosynthesis capacity were identified in both HC-enriched species and ESRD-enriched species (Fig. S13a, b). We could hardly conclude that "flagellar/flagellin biosynthesis capacity may have important physiological significance in ESRD".

Main concern 2: P14. Line 44-Line 61.

"The gut microbiota appears to be an important originator of such toxins, and thus contributor to serum toxin levels in patients. Gut bacteria accounted for 12%-44% of uremic toxins serum concentrations variability in the Beijing cohort patients in our previous study [21]. Here we extend this finding to the Shanghai cohort and observe even higher values, of 21.3%-47.8% and 27.9%-56.4% in the ESRD patients of Shanghai and Beijing cohorts, respectively. Predictability of serum concentrations of three AAA-derived toxins by gut microbiota was validated across patient cohorts, and to some extent even in the healthy controls. We suggest that such gut microbiota-based models might help support trials to test microbiome monitoring as a predictor of treatment outcome for dialysis patients and might also be applicable to individuals with a high risk of kidney disease or CKD, serving as noninvasive indicators for renal function evaluation."

Authors repeated the analysis in two separate cohorts and found that the ESRD-enriched species were consistently identified, this provides some level of confidence in the findings. However, the fact that the most discriminant species in the models for each toxin were very different between the two cohorts does raise some concerns about the reliability and generalizability of the results. They should also carefully consider why the occurrence rate of enzymes involved in uremic toxins (tyrosine phenol-lyase, hpdC, fldHBC) showed no differences between ESRD-enriched and HC-enriched species. I would suggest that the authors carefully consider these discrepancies and provide a clear explanation for why they may have occurred. In addition, the authors should consider additional analyses to help validate their findings or explore other possible explanations for the differences in the results. This will help to ensure that the findings are robust and can be generalized to other cohorts.

**Authors Response**

**Point-by-point responses to the reviewers’ comments:**

Below, we provide a detailed response to each reviewer's comments, with pour gratitude for their help in improving our manuscript. The reviewer comments are highlighted, followed by our responses.

Reviewer #1: The manuscript "metagenome-wide analysis uncovers gut microbial signatures and implicates taxon-specific functions in end stage renal disease" by Zhang et al. reported a comprehensive microbial analysis on two independent cohorts based on the metagenomic data. The authors demonstrated several findings: 1. Consistency with previous studies on depletion of Prevotella, Faecalibacterium and Coprococcus, as well as enrichment in Streptococcus, Fusobacterium, and Desulfovirionaceae. 2.Species in these genera were reported.3. Function prediction based on metagenomics. 4. Links between predicted functions, uremic toxins and bacterial species

**Response:** We appreciate the reviewer’s enthusiasm and helpful comments. Please see below our responses to his/her concerns.

Major concerns:

1. Based on the analysis, the authors proposed several important hypotheses. However, no mechanistic studies to prove or disprove the proposed hypothesis

**Response:** Thanks for your insightful comment. Although mechanistic research is our next direction, the primary aim of this study is to provide a comprehensive description of the gut microbiome characteristics of ESRD patients. Based on deep-metagenomic sequencing of patients and controls from two cohorts, we have identified numerous microbial and functional signatures associated with ESRD. Some of these signatures corroborate previously findings, such as the increased presence of opportunistic pathogens and toxin-producing bacteria, as well as the decreased abundance of SCFA-producing members in ESRD patients. However, we have also uncovered novel species and functions that exhibit differential abundance between patients and healthy individuals, shedding light on their potential roles in ESRD. For example, we found that ESRD patients have significantly reduced ability to utilize plant polysaccharides and biosynthesize small bioactive molecules. Notably, we have observed consistent ESRD-associated signatures in CKD patients, suggesting their potential impact on disease progression. Additionally, we have identified a group of species that contribute to toxin concentrations and demonstrated their predictive capabilities across different cohorts. Overall, while our study is observational in nature, we aim to provide valuable insights and directions for future mechanistic and clinical investigations.

In response to the reviewer's comment, we have included a limitation in the Conclusions section of the revised manuscript as follows: “*Although many of the hypotheses raised by this study have not been proven or disproven, we suggest that our results and resources will promote future mechanistic and therapeutic works in kidney diseases.*”

2. More details are required for random forest machine learning. What information was used for training? Any pre-analysis (e.g. LEfSe, correlation) was done on the microbiota data for the training?

**Response:** Thank you for providing this valuable comment. In Figure 1c, we employed the random forest machine learning algorithm to distinguish between ESRD patients and healthy controls based on their relative abundances of gut species. For intra-cohort discrimination (purple and blue lines in Figure 1c), 70% of the samples were randomly selected to train a random forest model, and the remaining 30% were used as the testing set to evaluate the predictive performance of the model. In order to achieve the best predictive performance, we used the relative abundances of the top 100 most important species in each cohort (Beijing and Shanghai) for model training and testing (Figure S5 demonstrates that these 100 species yielded the highest AUCs). For cross-cohort, all samples of one cohort were used for training, and all samples of the other cohort were used for testing. To ensure optimal predictive performance, we utilized the relative abundances of the top 100 most important species for the Beijing model (testing in the Shanghai cohort) and the top 50 most important species for the Shanghai model (testing in the Beijing cohort) (Figure S5). Based on your suggestion, we have included a supplementary table (Table S5) that displays the names and importance rankings of the selected species (i.e., the top 100 important species) for the Beijing and Shanghai models. We have also revised the descriptions of the random forest models in the legend of Figure 1c, Figure S5, and the Methods section accordingly. Furthermore, we would like to clarify that no pre-analysis was performed on the microbiota data prior to training and testing.

3. Regarding this conclusion "the Bacteroidaceae species (containing 4 ESRD-enriched and 2 HC-enriched species, Fig1d) were more likely to utilize animal glycans,……." I don't believe the referred Figure 1d, 3e, S16a and S16b support this conclusion. Authors may clarify this statement

**Response:** Thank you for providing this constructive criticism. Based on your suggestion, we have included a new figure (Figure 3d) that directly illustrates the differences in the utilization of animal carbohydrates and plant cell wall carbohydrates among several Bacteroides clades, including Bacteroidaceae, Muribaculaceae, Prevotellaceae, Rikenellaceae, and others. This analysis highlights that Bacteroidaceae species exhibit a significantly higher ability to utilize animal glycans compared to the Prevotellaceae and Muribaculaceae members (Wilcoxon rank-sum test *q* = 0.014 and 0.004, respectively). The addition of this figure provides visual illustration of our findings.

4. In the section "Gut bacteria affect serum uremic toxin level". More details for "the gut microbiota-based regression models" would be beneficial. How was this generated?

**Response:** Thanks for your valuable comment. In this section, we utilized a gut microbiota-based regression model to estimate the predictability of serum concentrations of uremic toxins based on the relative abundance profiles of gut species. A detailed description of the methodology for this regression model has been provided in our previous study [1]. Briefly, for each uremic toxin (i.e., IS, PAG, PCS, and TMAO), firstly, we trained a random forest model based on the relative abundances of all gut species and used a leave-one-out cross-validation approach to predict the serum concentration of this toxin based on the model. The coefficient of determination (R^2^) between the predicted values and the actual values served as an indicator of the gut species’ explanatory power for that particular toxin. Then, to achieve higher explainability, we ranked all the species based on their importance (as measured by the increase mean square error [IncMSE] within the model) and sequentially selected a specific number of species to train a new model and evaluate its performance. This iterative process enabled us to identify the model with the highest performance (as estimated by R^2^) and a corresponding set of training species. The highest performance achieved reflected the gut microbiota's ability to explain the variation in toxin levels. We have incorporated these details into the Methods section (specifically, the paragraph titled "Gut microbiota-based regression model") in the revised manuscript, following your suggestion.

Reference

[1] Wang X, Yang S, Li S, et al. Aberrant gut microbiota alters host metabolome and impacts renal failure in humans and rodents. *Gut*, 2020, 69(12): 2131-2142.

Minor concerns:

1. Why PC2 (7.8%) and PC3 (6.1%) were used to present Figure 1a? Figure S4 showed a better contribution by PC1 (15.1%).

**Response:** Thanks for pointing this out. Please refer to the follow supporting figure for the PCoA plot, for which the samples are shown at the first and second principal coordinates (PC1 and PC2). There is a clear separation between Beijing and Shanghai cohorts on PC1, but no significant difference on PC2 and PC3. This distinct separation is also evident in the table of Figure S4 (bottom right corner), where both ESRD patients and healthy individuals are clearly distinguished on PC1, PC2, and PC3, with the primary differentiation between Beijing and Shanghai samples occurring on PC1. Therefore, to prevent potential confusion arising from the effect of cohort origin, we chose to display PC2 and PC3 rather than PC1 in Figure 1a.

|  | **Supporting figure.** PCoA analysis of the Bray-Curtis distances of gut microbial species. Samples are shown at the first and second PCs, and the variance ratios contributed by these two PCs are displayed. Ellipsoids represent a 95% confidence interval surrounding each group. |
| --- | --- |

2. The first letters of some bacterial names were not capitalized.

**Response:** Thanks for pointing this out. We have thoroughly reviewed the spelling of all bacterial names and hopefully rectified all errors in the revised manuscript.

3. Several articles have shown that modification of gut microbiota and metabolite butyrate attenuates renal disease. PMID: 36164984, 31366236. These would help with the discussion.

**Response:** Thanks for this valuable comment. We have incorporated the suggested citations (references #45 and #46) into the Discussion section of the revised manuscript.

Reviewer #2: In this manuscript, the authors studied the gut microbial (GM) characteristics in patients with end-stage renal disease (ESRD). They applied shotgun metagenomic sequencing, assembled reads and analyzed the gut microbial data on MAGs and gene levels. They reported distinct gut microbial features in ESRD compared to non-ESRD controls, such as the disease-enriched MAGs (Blautia spp., Dorea spp., and Eggerthellaceae) and the and multiple taxon-specific functions which might potentially contribute and thus predict the serum uremic toxin concentrations in patients. They also validated several ESRD-related microbial features in another cohort (also from the same group) including ESRD patients, non-dialyzed chronic kidney disease (CKD) patients and healthy controls. The main concern is the novelty of this study when compared to their previous one. The group has published their first work on the ESRD-related gut microbial features in Gut (2019) (https://gut.bmj.com/content/69/12/2131.long). They applied shotgun sequencing, used assembly-based strategy, and reported the enrichment of multiple Firmicutes species in ESRD (e.g., Eggerthella lenta), and these contributed a lot to the toxin production. In addition, the capacity of multiple Firmicutes members for phenol- and p-cresol-producing have been demonstrated in numerous papers (ref, 10.1093/femsec/fiy125). However, the authors did not provide detailed information on the functional potentials on toxin-producing genes. They only used association analysis to report relationships between ESRD-enriched MAGs and toxin levels. They used their previous data (named as Beijing cohort) in combined the newly added Shanghai cohort to assembly MAGs and reanalyze their data on this topic. Most of their findings are descriptive and lack of a deep discussion. Also, a lot of information presented in their figures were not mentioned in the text. The second concern is the lack of rigor in the statistical methods and related descriptions (e.g., lack of sample number /statistical methods/ P & Q-values in most of the figure legends). (e.g., lack of a robust criterion for the identification of ESRD-related microbial species/functions. Another issue is too much information (a total 24 additional figures) but few of them were well described or discussed. I suggested that the authors should carefully review their manuscript and eliminate any unnecessary results or redundancy. This will help to streamline the paper and make it more focused. It's important for the authors to be transparent about the limitations and potential sources of variability in their data, and to provide a clear rationale for their findings. They need to address below issues before the paper can be accepted.

**Response:** We sincerely appreciate the reviewer for encouraging and insightful comments. We have carefully considered each of the points raised and addressed them in the revised manuscript. Regarding the novelty of our study, we have placed emphasis on the novel and significant findings. Specifically, we have highlighted the following key contributions:

1) We have provided a comprehensive and systematic characterization of the gut microbial characteristics in ESRD, offering a valuable resource for future research in this field.

2) By demonstrating the consistency of ESRD-associated signatures in CKD patients, we have revealed the potential impact of these signatures on the progression of CKD.

3) Our identification of toxin-contributing species and their predictive ability for toxin concentrations across cohorts is a novel and important finding.

In response to the concern about toxin-producing genes, we have performed a comparison of the abundances of toxin-producing genes between the fecal metagenomes of ESRD patients and healthy controls. We have included the results as a new supplementary figure (Figure S16) in the main text, providing additional evidence to support our findings.

Regarding statistical methods, we have thankfully implemented your suggestion We now employ a new standard (combined q<0.05 instead of combined P<0.005) to identify ESRD-associated species and functions. We have revised all results, figures, and tables accordingly. Additionally, we have provided more detailed descriptions of other statistical methods, such as the random forest regression model, in the Methods section to enhance the transparency of our methodology.

Furthermore, based on your suggestions, we have made necessary revisions by removing redundant results and eliminating unimportant information to streamline the manuscript. Please find below the point-to-point responses addressing your valuable comments.

**Part 1 The gut microbiome stratifies ESRD patients from healthy controls**

P7, line 52-52, which data showed 0.68%? Lack of figure or table? "*In ESRD patients, the underlying kidney disease (e.g., glomerulonephritis or diabetic kidney disease) explained only 0.68% (p=0.23) of the gut microbial variations.*"

**Response:** Thank you for bringing this to our attention. We have incorporated the result of 0.68% into the sentence by referencing Figure 1b, specifically the item "underlying kidney disease." This change has been made in the revised manuscript, following your valuable suggestion.

P7, Figure 1 and Fig. S5. Both Shanghai model and Beijing model showed extremally high AUCs (>0.9) by using ~100 RF selected MAGs, even in testing set. Could the author provide the importance for selected MAGs in their RF models? Which MAGs have the highest importance values (e.g., GINI)? How many MAGs were consistently selected for two models and how about the consistency of the top features in two models?

**Response:** Thanks for providing this valuable comment. As per your suggestion, we have included a supplementary table (Table S5) in the revised manuscript to present the selected microbial species (i.e., top 100 important species) for modeling in each cohort. In the Beijing cohort, the highest importance species included *Fusobacterium ulcerans M398*, *Acutalibacteraceae M215*, *Eggerthella lenta M4B3*, *Massilimicrobiota M4B6*, *Erysipelatoclostridium spiroforme M16D*, and others (ordered by mean decrease accuracy). Similarly, in the Shanghai population, the highest importance species comprised *Intestinimonas massiliensis M0D3*, *Lachnospiraceae M428*, *Anaerovoracaceae M0CF*, *Oscillospiraceae M4B8*, and *Prevotella M231*, among others. It is worth noting that 38 out of the top 100 important species were shared between the two cohorts. Considering the total number of species exceeds 1,300, this finding demonstrates a high consistency in the selection of these species between the two cohorts (Chi-squared test *p*=1.8x10^-29^ compared to random).

**Part 2 Microbial species related to ESRD**

In the method (Page 19), the authors stated that "*The following criteria were used for identifying ESRD-associated taxonomic and functional signatures from two independent cohorts: 1) q<0.2 and fold change >1.2 in each cohort with coherent enrichment in patients or controls, and 2) combined p<0.005.*" (Fig. S6).

First, it is wired that why the author used the criterion FD>1.2. Because their Volcano plot (Fig.S6) already showed that most of their MAGs including the not significant ones showed a FD>1.2 between two groups. Think they could just remove this criterion.

**Response:** Thanks for this insightful comment. We appreciate the reviewer's perspective, and indeed, the presence of the FD>1.2 criterion has limited impact on the results (375 differential species vs. 348 differential species in the absence vs. presence of the criterion, respectively). It is worth noting that the use of FD as a criterion to identify differentially abundant species is widely employed in other studies. Furthermore, considering the unequal sample sizes between the Beijing and Shanghai cohorts, solely relying on the *q*-value may result in the inclusion of more species with smaller FD in the cohort with the larger sample size. Therefore, we have chosen to retain the FD>1.2 criterion as one of the criteria for identifying ESRD-associated species.

Second, why they used combined P<0.005 (Q<0.01) to define the ESRD-related features? Why not a combined Q<0.05 ? Or combined the features with a Q<0.05 in each independent cohort, should this be more common? Their data already showed that in the same 348 MAGs, 277 and 320 MAGs were significantly differed in SH and BJ cohorts, respectively (Q<0.05 in each study). Or a stricter but simpler criterion could be the shared MAGs features pass the Q<0.05 in each cohort. PS, it is not clear how authors calculate the q values in the combined dataset? Based on 1,303 MAGs? Or based on other datasets?

**Response:** Thank you very much for your professional and constructive comments. Regarding the comparison between combined P<0.005 (Q<0.01) vs. Q<0.05, we acknowledge that our previous q-value calculation based on shared species (q<0.2 and FD>1.2, with coherent enrichment in patients or controls; Figure 6a) between the Beijing and Shanghai cohorts. This algorithm resulted in an overestimation of the true q-values. In response to your suggestion, we recalculated the q-values using the combined p-value of all 1,303 species, where a combined p<0.005 corresponds to a q-value of 0.027. Consequently, in the revised manuscript, we adopted Q<0.05 as the threshold for identifying differential species and identified a total of 353 ESRD-associated species, including all 348 species previously identified. We have updated all the results associated with these species accordingly.

As for the criterion of Q<0.05 in each cohort (a stricter but simpler criterion), we aimed to integrate data from both cohorts to identify additional signatures. We were particularly interested in identifying bacteria that were highly significant in one cohort (q<0.05) but exhibited close-to-significant results in the other cohort (q=0.05-0.20). Out of the 348 differential species identified, 265 species showed significance in both cohorts (Q<0.05), 12 species were significantly enriched in Shanghai but not in Beijing, 55 species were significantly enriched in Beijing but not in Shanghai, and 16 species were not significant in either cohort individually but had a significant combined q-value. These species exemplify the advantage of integrating data from both cohorts for identifying differential species.

Think the authors should improve their methods to ensure a robust definition of ESRD-related MAGs. Please revised the results accordingly (P8, line9-line18). "*Some 348 differentially abundant species between the ESRD patients and healthy controls were identified using the combined significance level in the two cohorts (combined Wilcoxon rank-sum test p<0.005, corresponding to q<0.01; Additional file 2: Fig. S6); 221 were more abundant in patients and 127 in controls. All species remained significant upon adjustment for sex, age, and BMI, and, a majority (73.6%) had a significant (q<0.05) and coherent enrichment in both cohorts (Additional file 1: Table S5)*."

**Response:** Thank you for your helpful comments. Based on your suggestions, in the revised manuscript, we used the combined *q*<0.05 as the criterion to identify ESRD-associated species, as well as KEGG functions and modules, and made corresponding modifications to the results, figures, and tables. Similarly, the adjusted definition of ESRD-associated signatures has been revised in the Methods section as well.

P8. Line21-Line22. "12 of 18 proteobacteria were enriched in controls". The authors did not discuss this result. It is weird that most (12/18) of proteobacteria were enriched in the controls. As discussed in a previous review (doi: 10.1093/advances/nmz028), most of the human studies have consistently found an enrichment of Gammaproteobacteria/ Enterobacteriaceae in the ESRD/CKD patients (pre or post dialysis) and these group are well known phenol producers.

**Response:** Thanks for this insightful comment. Indeed, previous studies had found that the Enterobacteriaceae species are more abundance in ESRD/CKD patients. However, in this study, we found that although several Enterobacteriaceae species, such as *Escherichia coli M1FD*, *Escherichia fergusonii M454*, and *Citrobacter freundii M2BF*, appeared to be more prevalent in the ESRD population, none of these species was significant (see supporting figure below). Similarly, there were no significant differences in other Enterobacteriaceae species between ESRD patients and healthy individuals. This result may be related to the specificity of our current population.

**Supporting figure.** Heatmap showing the relative abundances of 17 Enterobacteriaceae species in ESRD patients and healthy controls. Each row represents a KEGG ortholog with the scaled relative abundance among all individuals and each column represents an individual, ordered by hierarchical clustering.

Regarding the ESRD-associated proteobacteria, we have identified 12 proteobacteria species that are enriched in HC, including 5 *Haemophilus* species, 4 *Parasutterella* species, and 3 others (e.g., *Duodenibacillus massiliensis*, *Turicimonas*) (see supporting table below). *Haemophilus* is an opportunistic pathogen, but it is also commonly present in the gut microbiota of healthy individuals. Despite some studies found the enrichment of *Haemophilus* in patients with colon cancer [1] or multiple sclerosis [2], conversely, the decrease in *Haemophilus* species has also been found in patients with type 2 diabetes [3], rheumatoid arthritis [4], or mental diseases [5]. Therefore, whether the *Haemophilus* in gut microbiota can have a positive or negative effect on human health remains unknown. *Parasutterella* has been defined as a core component of the human gut microbiota and may play a beneficial role in mucosal homeostasis. On the other hand, we have identified 6 proteobacteria species enriched in ESRD patients, including 3 *Sutterella* species, 2 *Desulfovibrio* species, and 1 *Azospirillum* species. Similar to *Parasutterella*, *Sutterella* may also be a beneficial bacterium [6], and its role in patients with ESRD still needs to be evaluated. *Desulfovibrio* in the gut is thought to be associated with excessive meat consumption and may increase the risk of various diseases, so its enrichment in ESRD patients needs attention. *Desulfovibrio* is a common gut bacterium that has the ability to reduce sulfate and can help digest food and promote nutrient uptake. However, excessive levels of *Desulfovibrio* may be associated with the development of certain diseases such as colorectal cancer [7], and Parkinson’s disease [8]. Thus, the enrichment of *Desulfovibrio* in ESRD patients may be of interest.

|  | **Supporting table.** Detailed information of 18 ESRD-associated Proteobacteria species. |
| --- | --- |

References

[1] Kasai C, Sugimoto K, Moritani I, et al. Comparison of human gut microbiota in control subjects and patients with colorectal carcinoma in adenoma: Terminal restriction fragment length polymorphism and next-generation sequencing analyses. *Oncology reports*, 2016, 35(1): 325-333.

[2] Chen J, Chia N, Kalari K R, et al. Multiple sclerosis patients have a distinct gut microbiota compared to healthy controls. *Scientific reports*, 2016, 6(1): 1-10.

[3] Zhang X, Shen D, Fang Z, et al. Human gut microbiota changes reveal the progression of glucose intolerance. *PloS one*, 2013, 8(8): e71108.

[4] Zhang X, Zhang D, Jia H, et al. The oral and gut microbiomes are perturbed in rheumatoid arthritis and partly normalized after treatment. *Nature medicine*, 2015, 21(8): 895-905.

[5] Zhu C, Zheng M, Ali U, et al. Association between abundance of Haemophilus in the gut microbiota and negative symptoms of schizophrenia. *Frontiers in Psychiatry*, 2021, 12: 685910.

[6] Kaakoush N O. Sutterella species, IgA-degrading bacteria in ulcerative colitis. *Trends in Microbiology*, 2020, 28(7): 519-522.

[7] Yachida S, Mizutani S, Shiroma H, et al. Metagenomic and metabolomic analyses reveal distinct stage-specific phenotypes of the gut microbiota in colorectal cancer. *Nature medicine*, 2019, 25(6): 968-976.

[8] Murros K E, Huynh V A, Takala T M, et al. Desulfovibrio bacteria are associated with Parkinson’s disease. *Frontiers in Cellular and Infection Microbiology*, 2021, 11: 652617.

P8，Line38-Line 43 "*Notably, some closely related species exhibited opposite trends between patients and controls (Additional file 2: Fig. S8), suggesting that the difference in functions they encode may be related to ESRD.*" Lack of details in results.

**Response:** Thanks for pointing this out. It is indeed common in microbiome studies to observe different responses to disease among bacteria belonging to the same taxonomic group (e.g., genus) due to variations in their encoded functions. In Figure 8a, for instance, *Bacteroides fragilis* (enriched in ESRD patients) is known as a significant pathobiont in the human gut microbiome and has been associated with multiple diseases [1]. On the other hand, *Phocaeicola plebeius* (formerly named *Bacteroides plebeius*, enriched in healthy subjects) plays a crucial role as a decomposer in the human gut, participating in the metabolism of various complex polysaccharides [2-4]. Furthermore, recent research has indicated that Bacteroides plebeius improves muscle wasting in chronic kidney disease by modulating the gut-renal muscle axis [5]. In Figure S8b, *Bifidobacterium dentium* (enriched in ESRD patients) is commonly found in human oral and fecal samples and has been associated with dental caries [6]. While some studies have suggested potential probiotic effects of *Bifidobacterium dentium* [7,8], its effects in the human intestine have not been fully confirmed. In contrast, *Bifidobacterium adolescentis* (enriched in healthy controls) is a well-known probiotic widely used in functional foods. Regarding Figure S8c, limited studies have focused on the *Dorea* genus. Previous research has suggested that *Dorea longicatena* (enriched in healthy subjects in our study) may promote obesity [9]. However, there is currently insufficient research on ESRD-enriched *Dorea faecis* and *Dorea scindens.* We compared the functional profiles of all Dorea bacteria and found distinct functional differences between ESRD-enriched and HC-enriched strains (as shown in the supporting figure). Considering that our subsequent analyses involve comparing the functions of bacteria within the same taxonomic group, such as the comparison between ESRD-enriched Firmicutes and HC-enriched Firmicutes, we have decided to remove this sentence in the revised manuscript to strengthen our main results.

|  | **Supporting figure.** PCoA analyses of the functional profiles of *Dorea* species. |
| --- | --- |

References

[1] Tajkarimi M, Wexler H M. CRISPR-Cas systems in Bacteroides fragilis, an important pathobiont in the human gut microbiome. *Frontiers in microbiology*, 2017, 8: 2234.

[2] Hehemann J H, Kelly A G, Pudlo N A, et al. Bacteria of the human gut microbiome catabolize red seaweed glycans with carbohydrate-active enzyme updates from extrinsic microbes. *Proceedings of the National Academy of Sciences*, 2012, 109(48): 19786-19791.

[3] Munoz-Munoz J, Ndeh D, Fernandez-Julia P, et al. Sulfation of arabinogalactan proteins confers privileged nutrient status to Bacteroides plebeius. *Mbio*, 2021, 12(4): e01368-21.

[4] Robb C S, Hobbs J K, Pluvinage B, et al. Metabolism of a hybrid algal galactan by members of the human gut microbiome. *Nature chemical biology*, 2022, 18(5): 501-510.

[5] Pei T, Zhu D, Yang S, et al. Bacteroides plebeius improves muscle wasting in chronic kidney disease by modulating the gut‐renal muscle axis. *Journal of Cellular and Molecular Medicine*, 2022.

[6] Mantzourani M, Fenlon M, Beighton D. Association between Bifidobacteriaceae and the clinical severity of root caries lesions. *Oral microbiology and immunology*, 2009, 24(1): 32-37.

[7] Engevik M A, Danhof H A, Hall A, et al. The metabolic profile of Bifidobacterium dentium reflects its status as a human gut commensal. *BMC microbiology*, 2021, 21(1): 154.

[8] Pokusaeva K, Johnson C, Luk B, et al. GABA‐producing Bifidobacterium dentium modulates visceral sensitivity in the intestine. *Neurogastroenterology & Motility*, 2017, 29(1): e12904.

[9] Liu R, Hong J, Xu X, et al. Gut microbiome and serum metabolome alterations in obesity and after weight-loss intervention. *Nature medicine*, 2017, 23(7): 859-868.

**Part 3 Microbial functions related to ESRD**

P8，Line 59-Line61. Please define the significance cutoff for functional KO and modules. (Figure S10). Also please keep an eye on the above suggestions on ESRD-related species. It is weird as the combined P<0.005 equals to different q values in different datasets (between species, KO and modules).

**Response:** Thanks for your valuable comments. Following your suggestion, we have conducted a recalculation of the q-values using all KOs and modules, and have revised the significance threshold to Q<0.05. With this updated criterion, we have identified a total of 1,279 ESRD-associated KOs and 103 modules, which represents an increase of 13 KOs compared to the previous analysis, while the number of modules remains unchanged. We have made necessary modifications to the figures, tables, and relevant text to accurately reflect these revised results in the updated manuscript.

P9, Line1-Line5. "*Modules that were most highly ESRD-biased involved antimicrobial resistance and bacterial toxicity, phosphotransferase systems for monosaccharide (e.g., fructose and mannose) uptake, secondary metabolite biosynthesis, and transport systems (Fig. 2c, d; Additional file 1: Table S6-7)*". It is very hard for readers to get the conclusion based on the heatmap based on Kos (Fig. 2c). Also, if we read the heatmap very carefully, we could find the ESRD patients have two distinct patterns in these functional modules. As shown below, only half of the patients have enrichment (in red) while the rest seemed to have comparable abundances as healthy controls? Did the authors have any results on this part? Think it is a very important topic and worth a try to improve the novelty of the work.

**Response:** Thank you for bringing up this important question that warrants further investigation. We thoroughly examined the function-level PCoA analysis encompassing all individuals (Figure S7 in the revised manuscript) and observed no discernible stratification in the functional composition of ESRD patients at the KEGG or module levels. To address the concern raised by the reviewer, we conducted clustering analysis specifically on the ESRD patients using the K-means algorithm based on their KEGG composition. The performance of clustering was assessed using the average silhouette width (ASW). Our analysis indicated that the highest ASW was achieved when the number of clusters was set to 5 (refer to the supporting figure below). However, it is important to note that even with the highest ASW, the value obtained was only 0.15, which falls considerably below the threshold typically considered reliable for stratification in classification (ASW = 0.30). Consequently, we concluded that there is insufficient evidence to support the presence of distinct clusters in the functional profiles of ESRD patients.

Furthermore, in Figure 2c, we displayed the composition of 146 KEGG orthologs (KOs). Considering the total of 1,266 ESRD-associated KOs, we consolidated them and generated a comprehensive heatmap (refer to the supporting figure below), which also revealed a lack of clear stratification in the functional composition of ESRD patients. We appreciate your valuable input, as it has prompted us to conduct a thorough analysis, leading to a better understanding of the functional profiles in ESRD patients.

**Supporting figure. (A)** Average silhouette width (ASW) showing the optimal number of clusters for clustering analysis of the functional composition of all ESRD patients. The result shows poor support for the existence of discrete clusters (ASW <0.3 in all cases). **(B)** PCoA analysis of the functional composition of all ESRD patients. Samples are colored based on 5 optimal clusters. The first two principal coordinates and their explained variances are shown. **(C)** Heatmap showing the abundances of 1,279 ESRD-associated KOs in all subjects. Each row represents a KEGG ortholog with the scaled relative abundance among all individuals and each column represents an individual, ordered by hierarchical clustering.

P9, Line 7- Line9. "*The ESRD-depleted functions included inositol phosphate metabolism and ubiquinone and pyridoxal biosynthesis, among others.*" What is "among others"?

**Response:** Thanks for pointing this out. We have changed this expression into “*… the ESRD-depleted functions included inositol phosphate metabolism, ubiquinone and pyridoxal biosynthesis,* ***and others***” to avoid any potential confusion.

P9, Line 28-Line32. Line 33-Line38. "*The total abundance of ARGs increased 39.4% in ESRD patients as compared with the healthy controls (p<0.001; Additional file 2: Fig. S11a,b).*" "*The most prominent changes were observed in genes associated with* *multidrug resistance, beta-lactamase, aminoglycoside, and quinolone resistance (Additional file 2: Fig. S11c). These observations suggest that patients may have been more frequently exposed to antibiotics than the controls in the past, possibly due to treatment of dialysis-associated infections [29].*" What did the author mean? 39.4% of the 1,614 ARGs were increased in ESRD or the RPKM was 39.4% higher in ESRD than HC? Also, few information is provided by the AGR analysis. Think the author could delete this part and focused on their main findings.

**Response:** Apologies for the previous lack of clarity. In this analysis, we aggregated the abundance (RPKM) of all antibiotic resistance genes (ARGs) for each individual and observed that the average total abundance was 39.4% higher in ESRD patients compared to healthy controls (average total RPKM: 3,793 vs. 5,288, p = 3.0*10^-8^; refer to Figure S9b in the revised manuscript). Additionally, we examined the total abundance of different types of ARGs, categorized based on their antibiotic mechanisms. Our findings revealed that several types, including multidrug resistance, beta-lactamase, aminoglycoside, and quinolone resistance, were significantly elevated in ESRD patients when compared to healthy controls (refer to Figure S9c in the revised manuscript). Considering the compromised immune system of ESRD patients and their frequent use of antibiotic drugs, we believe that the ARG profiles and signatures derived from the gut microbiota of ESRD patients could serve as valuable insights for future clinical research. Therefore, we have retained this section in the revised manuscript and made clarifications to enhance the understanding of our results. The modified content is provided below:

“*The ESRD patients exhibited an average 39.4% increase in the overall relative abundance of ARGs compared with the healthy controls (p<0.001; Additional file 2: Fig. S9a-b). This increase was particularly notable for several antibiotic resistance mechanisms, including multi-drug resistance, beta-lactamase, aminoglycoside, and quinolone resistance (Additional file 2: Fig. S9c). These findings suggest that patients may have experienced more frequent exposure to antibiotics in the past than the controls, possibly due to the treatment of dialysis-associated infections [29].*”

P10, Line1-Line4. "*These KOs were mostly within several categories; for example, enzymes involved in antimicrobial resistance and degradation of aromatic compounds were more widespread in ESRD-enriched species, whereas those associated with cofactor and vitamin metabolism and AAA biosynthesis were mostly present in HC-enriched species (Fig. 3a).*" Please clarify which cofactor or vitamin metabolism-related KOs are enriched in HC-enriched species? (VB2? VB7? ??) No useful information could observe in the heatmap.

**Response:** Thanks for pointing this out. We have included the detailed information of all the differential KOs in Table S9. Among these, there are 38 KOs related to cofactor or vitamin metabolism. These KOs are distributed across various categories, including porphyrin and chlorophyll metabolism (n = 9), thiamine metabolism (n = 7), folate biosynthesis (n = 7), riboflavin metabolism (n = 5), vitamin B6 metabolism (n = 3), nicotinate and nicotinamide metabolism (n = 3), and others (n = 4). In response to your suggestion, we have provided a clearer description of these results as follows:

"*These KOs mainly fell into several categories, with enzymes involved in antimicrobial resistance and degradation of aromatic compounds being more widespread in ESRD-enriched species, while those associated with cofactor and vitamin metabolism* ***(e.g., porphyrin, thiamine, and folate)*** *and AAA biosynthesis were mostly present in HC-enriched species (Fig. 3a).*"

It is reasonable to compare the functional configuration of the ESRD-enriched Firmicutes and HC-enriched controls (a, b). However, I do not think the comparisons between ESRD- and HC-enriched non-Firmicutes make sense. It is also hard for readers to understand the related Fig S12 (no P values, no details for the effect size). Because these MAGs enriched in different groups are from different phylogenetic levels (even at the phylum level). For instance, in Fig. 3c, we could find that multiple "Specific transporters of ESRD-enriched species" were detected in HC-enriched species. And most HC-enriched were gram-negative species and therefore harbored LPS-related functions. I do not think these comparisons could provide valuable information for clinical researcher in this field.

**Response:** Thank you for providing this valuable comment. We acknowledge and agree with the reviewer's viewpoint that direct comparisons between ESRD-enriched and HC-enriched species in non-Firmicutes may not yield meaningful results due to their phylogenetic differences. Therefore, we have made appropriate modifications to this section in the revised manuscript to minimize such comparisons and descriptions of non-Firmicutes. As a result, Figure 3d, Figure S15, and Table S11-12, which had reduced significance, have been removed. The revised content of this section is as follows:

“*Beyond Firmicutes, we observed significant functional differences between ESRD-enriched and HC-enriched species belonging to other phyla. The non-Firmicutes ESRD-enriched species exhibited a higher prevalence of antibiotic resistance genes, bacterial toxicity-related enzymes, and enzymes involved in arginine and histidine metabolism, while the HC-enriched species encoded a higher presence of enzymes involved in the metabolism of folate, ubiquinone, and LPS (Fig. 3c; Additional file 2: Fig. S12); these functional differences may potentially be associated with ESRD.*”

Furthermore, as per your suggestion, we have included P-values and effect sizes in Figure S12 (Figure S10 in the revised manuscript).

P10, Line 9-Line 13. The author stated that "*Proteins associated with bacterial motility (flagellar biosynthesis) were mainly encoded by the HC-enriched Lachnospira, Roseburia, and Agathobacter (Additional file 2: Fig. S13a, b)*. However, in FigS13 a, b, we could observe that species enriched in both patients and HC encoded almost the same potential functions involved in bacterial motility.

**Response:** Thank you very much for pointing this out. We apologize for the error, and the correct figures should be Figure S13c-d instead of Figure S13a-b. In Figure 3a and Figure S13c-d, we observed that enzymes related to bacterial motility and chemotaxis are present in both HC-enriched species and ESRD-enriched species. Specifically, 21 HC-enriched species, mainly belonging to *Lachnospira*, *Roseburia*, and *Agathobacter*, encoded a total of 43 of these enzymes, while 15 ESRD-enriched species, including *Hungatella* and other Firmicutes, encoded 35 of these enzymes. However, considering that the number of HC-enriched species is much smaller than that of ESRD-enriched species (57 vs. 149), statistically, the occurrence frequency of these enzymes is significantly higher in HC-enriched species compared to ESRD-enriched species (average occurrence rate ~37% vs. ~10%, p << 0.001 by Fisher's exact test). Therefore, we conclude that HC-enriched species possess a significantly higher capacity to synthesize these functions compared to ESRD-enriched species. We have clarified these results in the revised manuscript to avoid misleading.

P10. Line 50-Line 60. For another instance (Fig. 3e), we could hardly conclude that "*Genomic annotation of carbohydrate-active enzymes (CAZymes) revealed that, consistent with previous studies [32, 33], the Bacteroidaceae species were more likely to utilize animal glycans, whereas the HC-enriched Prevotellaceae and Muribaculaceae members were likely to use plant glycans such as xylan, starch, and pectin*" based on the PCoA analysis (Fig. 3e). Instead, both the figure 3e and the related Fig. S16 suggested similar CAZyme profiles between the NS (not significant) species and the HC-enriched species. These data more likely suggested different CAZyme profiles between different phylogenetic members (e.g., Bacteroidetes vs. Prevotellaceae and Muribaculaceae), but not suggested the different CAZyme profiles between disease-enriched species and HC-enriched ones. The author should be very careful when interpret these results. Otherwise, I would suggest they delete un-related findings/ figures, revise their analysis and focus on more important topics. (e.g., why they do not present the results from Fig.S17 in the main figure)

**Response:** In this study, we observed that the majority of Bacteroidetes were enriched in healthy controls, specifically species belonging to the Prevotellaceae and Muribaculaceae families. Unlike Bacteroidaceae, which is the most dominant taxon in the human gut and primarily specializes in animal polysaccharide degradation, Prevotellaceae and Muribaculaceae are more inclined towards plant polysaccharide degradation (see supporting figure below). Based on this finding, we propose the hypothesis that ESRD patients may experience a reduction in their capacity to degrade plant polysaccharides due to the decrease in Prevotellaceae and Muribaculaceae. To further support this hypothesis, we conducted a fecal metagenome analysis of carbohydrate-active enzymes (CAZy) (see supporting figure below), which confirmed an overall lower plant polysaccharide degradation capacity in the gut microbiota of ESRD patients. Following the reviewer's suggestion, we have clarified these results and descriptions, as well as rearranged the figures in the revised manuscript. The revised content is as follows:

“*We then focused on the polysaccharide utilization capacity of the Bacteroidetes species since they are primary polysaccharide degraders in the human gut [31]. Genomic annotation of carbohydrate-active enzymes (CAZymes) revealed that, consistent with previous studies [32, 33], Bacteroidaceae species were more likely to utilize animal glycans, whereas Prevotellaceae and Muribaculaceae members (most of which were HC-enriched) were more likely to use plant glycans such as xylan, starch, and pectin (Fig. 3d; see Additional file 2: Fig. S13 for polysaccharide utilization locus analysis). Expanding the analysis to include all CAZyme genes found in fecal metagenomes, in addition to those carried by the assembled genomes, revealed that the microbiome of healthy controls had a higher abundance of plant polysaccharide-degrading genes (Fig. 3e). Interestingly, there was no strong correlation between polysaccharide utilization capacity and the relative abundances of Prevotellaceae/Muribaculaceae with meat or vegetable consumption (Additional file 2: Fig. S14), suggesting that the correlation between ESRD status and polysaccharide utilization is not highly dependent on diet.*”

**Supporting figure (Figure 3d-e in the revised manuscript). (d)** Boxplot showing the polysaccharide utilization capacity, including animal carbohydrates, plant cell wall carbohydrates, and mucin utilization, of Bacteroidetes species. Nodes represent the species and its colors represent the enrichment of the species: red, enriched in ESRD patients; blue, enriched in healthy controls; gray, not significant. **(e)** Boxplot showing the comparison of enzymes involving to polysaccharide utilization in the gut microbiome of ESRD patients and healthy controls.

P11. Fig. 3f. Please provide methods for correlations between MAGs and functions on legends. Please define what is "more frequent" or "significantly more frequent" in this section.

**Response:** Thanks for pointing this out. In response, we have included the methods detailing the connections between taxa and functions in the figure legends. Additionally, we have made changes to the manuscript to replace the phrases "more frequent" and "significantly more frequent" with "more prevalent/common" and "significantly more prevalent," respectively.

**Part 4 Gut bacteria affect serum uremic toxin levels**

P12. Fig4.

It is very interesting that the Oscillospiraceae MAGs (half were disease-enriched) showed very close and positive relationships with the uremic toxin levels. However, they did not go deeper to investigate the potential enzymes in the Oscillospiraceae MAGs. Since they already have the KO profiles, I would suggest they provide more data on this part. The authors repeatly stated that "The 67 gut species with the highest contribution to serum toxin levels. However, they did not provide any functional data to support whether these MAGs were previously toxin-producers or whether they harbored functional genes involved in toxin production? PS, It is also very interesting for readers whether the disease-enriched, toxin-related MAGs overlapped with those having the highest distinguish power between HC and patients in the RF models?

**Response:** Thank you very much for your constructive comment. Based on your suggestion, we have included the enzymes related to uremic toxin production by these 67 species in Table S12 of the revised manuscript. As anticipated, the majority of these species (50/67) were found to possess one or more toxin-producing genes. Although the prevalence of toxin-producing genes was relatively higher in Oscillospiraceae bacteria compared to other bacteria, the difference was not statistically significant (prevalence rate 92.6% vs. 72.5%, p = 0.096). To evaluate the discriminatory capacity of these bacteria in distinguishing between patients and healthy individuals, we compared the 67 species with the top 100 species with the highest discriminatory power in Table S5 (in the revised manuscript) and identified 9 and 10 species that were shared in the Beijing and Shanghai models, respectively. Considering the total number of species being over 1,300, this result indicates a relatively high but statistically non-significant proportion of shared species (Chi-squared test p = 0.142 and 0.058, respectively, compared to random distribution). Furthermore, we directly constructed random forest (RF) models using these 67 "toxin-contributing species" to distinguish ESRD patients from healthy controls and found that the models exhibited excellent discriminatory performance (AUC = 0.969 in the Beijing cohort, 0.948 in the Shanghai cohort; see supporting figure below). These findings collectively suggest that the 67 species may serve as differentiating factors between disease and health.

|  | **Supporting figure.** ROC analysis was performed to assess the classification performance of ESRD vs. control based on the abundances of the 67 "toxin-contributing species". In each cohort, 70% of the samples were randomly assigned to the training set for the random forest model, while the remaining 30% of the samples were used as the testing set. The AUC values and their corresponding 95% confidence intervals are presented below. |
| --- | --- |

In Fig20, very different MAGs were selected as the top species for the prediction of toxins in two cohorts. Why? Did the authors have any explanation?

**Response:** Thanks for this inquiry, which led us to extend our analysis. To better evaluate the consistency of the toxin-contributing species, we increased the number of compared species from 20 to 40 between the two cohorts (Figure S17 in the revised manuscript). This allowed us to assess their consistency more comprehensively. The results showed that for IS, PAG, PCS, and TMAO, there were 5, 13, 10, and 4 species, respectively, that were shared between the two cohorts. Considering the total number of species exceeding 1,300, this finding indicates a relatively high level of consistency between the two cohorts. The statistical analysis using the Chi-squared test yielded p-values of 0.0023, 1.1x10^-25^, 1.5x10^-14^, and 0.035, respectively, indicating significant consistency compared to random chance. Furthermore, we observed that although the number of shared species at the species level was limited, several species belonging to Oscillospiraceae and Ruminococcaceae had a significant impact on both PAG and PCS. For PAG, more than 40% (18/40 in Beijing and 16/40 in Shanghai) of the major contributing species belonged to Oscillospiraceae and Ruminococcus. Similarly, for PCS, nearly half (18/40 in Beijing and 19/40 in Shanghai) of the major contributing species belonged to Oscillospiraceae and Ruminococcaceae. We also observed a similar phenomenon for IS, where Bacteroidetes members played a significant role (23/40 in Beijing and 8/40 in Shanghai). This finding is consistent with previous studies that highlight the major contribution of Bacteroidetes to the serum concentration of IS in humans [1]. We have updated Fig. 20 (Figure S17 in the revised manuscript) based on your comment.

Reference

1. Devlin A S, Marcobal A, Dodd D, et al. Modulation of a circulating uremic solute via rational genetic manipulation of the gut microbiota. *Cell host & microbe*, 2016, 20(6): 709-715.

P12. "*In addition, Mantel test analysis revealed that the composition of these toxin-contributed species significantly correlates with the individuals' clinical parameters such as high-sensitivity C-reactive protein (hsCRP) and estimated glomerular filtration rate (eGFR) (Fig. 4c), suggesting that they potentially play critical roles in human physiological health.*" In my opinion, these correlations between so called "toxin-contributed species" and clinical parameters could largely contributed by the highly positive relationships between the toxin levels and these clinical parameters (eGFR and hsCRP). I don't think this observation could suggest their "*potentially play critical roles in human physiological health.*" Try and see if you could repeat it after adjustment for the toxin levels.

**Response:** Thanks for this valuable comment. As suggested, upon adjusting for toxin levels, we observed a significant decrease in the impact of these "toxin-contributing species" on eGFR (Mantel’s r = 0.067, *p* = 0.089 in patients and r = 0.031, *p* = 0.191 in controls) and hsCRP (Mantel’s r = 0.049, *p* = 0.401 in patients and r = 0.039, *p* = 0.143 in controls). This finding suggests that, as pointed out by the reviewers, these species may influence human physiological health through their effect on toxin levels. We have revised the sentence to read "*In addition, the Mantel test analysis revealed significant correlations between the composition of these toxin-contributing species and clinical parameters such as high-sensitivity C-reactive protein (hsCRP) and estimated glomerular filtration rate (eGFR) (Fig. 4c),* ***suggesting their potential roles in human physiological health***" in order to avoid overstating the findings.

**Part 5 Gut microbiota in non-dialyzed CKD patients**

P12. I would not suggest they use the FD>1.2 to report the tendency between case and controls as I stated before most of NS species also showed FD>1.2 between case and controls. Try to use effect size to report the tendency.

**Response:** Thanks for pointing this out. We conducted an analysis of effect sizes among four groups (HC, CKD3/4, CKD5N, and ESRD) and observed that the gut microbiome composition of CKD3/4 patients falls between that of ESRD patients and healthy controls (CKD3/4 vs. ESRD, effect size = 3.2%, *adonis p* = 0.013; CKD3/4 vs. HC, effect size = 5.7%, *adonis p* = 0.002). In contrast, there was no statistically significant difference observed between the microbiome of CKD5N and ESRD patients (effect size = 3.1%, *adonis p* = 0.19). We have made the necessary clarifications in the revised manuscript, as per your suggestion.

The author combined all the ESRD-enriched species and showed a gradually increased abundances from HC to ESRD and stated that "*the abundance of the ESRD-enriched and depleted species was significantly correlated with the severity of CKD (Fig. 5b), supporting the hypothesis that ESRD-associated bacteria might be related to CKD progression and the development of ESRD.*" It could be more valuable for reads and doctors to provide more information to show which MAGs are more related to disease severity but not the overall abundances of the 200+ MAGs.

**Response:** Thanks for this valuable comment. Based on your suggestion, we have identified 82 out of 353 ESRD-associated species that demonstrate a consistent positive or negative trend in relation to disease severity. These species consist of 53 ESRD-enriched species, including members of Lachnospiraceae (n = 17), Oscillospiraceae (n = 13), Clostridiaceae (n = 6), among others, and 29 HC-enriched species, including members of Prevotellaceae (n = 10), Lachnospiraceae (n = 6), Rikenellaceae (n = 5), and others. In the revised manuscript, we have incorporated these findings into the "Gut microbiota in non-dialyzed CKD patients" section, and we have also included Figure S19 (refer to the supporting figure below) to present the names and dynamics of these species.

**Supporting figure (Figure S19 in the revised manuscript).** Heatmap presented below illustrates the relative abundance variations of ESRD-associated species in healthy controls, as well as patients with CKD stage 3-4, CKD5N, and ESRD. The left panel displays the abundances of 53 ESRD-enriched species, while the right panel showcases the abundances of 29 HC-enriched species, both exhibiting a consistent positive or negative trend in relation to disease severity. Statistical significance, as determined by the Wilcoxon rank-sum test between the patients and controls, is denoted by asterisks: *, *q*<0.05; **, *q*<0.01; ***, *q*<0.001.

**Discussion:**

Authors should carefully summarize the key findings and their implications in the context of the current state of knowledge in the field (try not to overstate their findings). Also, please put more words on their novelty and/or significance of their findings.

**Response:** Thanks for this very helpful suggestion. In response, we have made significant revisions to the Discussion section, pointing out similarities between our findings and the current state of knowledge in the fieldand highlighting novelties of our key findings, which include: 1) Systematic identification of the gut microbial characteristics specific to ESRD, providing a comprehensive set of ESRD-associated signatures that can serve as valuable resources for future research. 2) Discovery of the consistent presence of these ESRD-associated signatures in patients with CKD, suggesting their potential role in CKD progression. 3) Identification of a group of species likely contributing to toxin production and their utility in predicting toxin concentrations across different cohorts. These findings underscore the originality and significance of our study, offering valuable insights into the understanding of ESRD-related gut microbiota and its potential implications for CKD progression.

Main concern 1: P13. Line 55-Line 65. P14. Line1-Line 5. "*For instance, we found that the proteins associated with flagellar biosynthesis were mainly encoded by the HC-enriched members of Lachnospira, Roseburia, and Agathobacter and the ESRD-enriched Hungatella spp. These species encode flagellin, which has been previously demonstrated to have immunomodulatory properties associated with multiple inflammatory diseases [40, 41]. In particular, Roseburia-derived flagellin is a negative regulator of gut inflammation [42] and shows protection effects in barrier functions [43]. These findings suggest that the flagellar/flagellin biosynthesis capacity may have important physiological significance in ESRD; we suggest that this research direction is worthy of further exploration.*"

As I mentioned above, most of the KOs involved in flagellar/flagellin biosynthesis capacity were identified in both HC-enriched species and ESRD-enriched species (Fig. S13a, b). We could hardly conclude that

"*flagellar/flagellin biosynthesis capacity may have important physiological significance in ESRD*".

**Response:** Thanks for pointing this out. As mentioned earlier, while both ESRD-enriched and HC-enriched species possessed enzymes involved in flagellar/flagellin biosynthesis, we observed a higher likelihood of these enzymes being present in the HC-enriched species. However, to ensure accuracy and avoid exaggeration, we have revised the discussion in the manuscript accordingly. The modified content is as follows:

“*For instance, we found that the proteins responsible for flagellar biosynthesis were more likely to be encoded by HC-enriched members of Lachnospira, Roseburia, and Agathobacter, as well as ESRD-enriched Hungatella spp. These species produce flagellin, which has previously been demonstrated to possess immunomodulatory properties associated with multiple inflammatory diseases [40, 41]. In particular, Roseburia-derived flagellin has been shown to act as a negative regulator of gut inflammation [42] and exhibits protection effects on barrier functions [43]. These findings suggest that the flagellar/flagellin biosynthesis capacity may hold potential significance in the context of ESRD, warranting further investigation in this research direction.*”

Main concern 2: P14. Line 44-Line 61. "*The gut microbiota appears to be an important originator of such toxins, and thus contributor to serum toxin levels in patients. Gut bacteria accounted for 12%-44% of uremic toxins serum concentrations variability in the Beijing cohort patients in our previous study [21]. Here we extend this finding to the Shanghai cohort and observe even higher values, of 21.3%-47.8% and 27.9%-56.4% in the ESRD patients of Shanghai and Beijing cohorts, respectively. Predictability of serum concentrations of three AAA-derived toxins by gut microbiota was validated across patient cohorts, and to some extent even in the healthy controls. We suggest that such gut microbiota-based models might help support trials to test microbiome monitoring as a predictor of treatment outcome for dialysis patients and might also be applicable to individuals with a high risk of kidney disease or CKD, serving as noninvasive indicators for renal function evaluation.*"

Authors repeated the analysis in two separate cohorts and found that the ESRD-enriched species were consistently identified, this provides some level of confidence in the findings. However, the fact that the most discriminant species in the models for each toxin were very different between the two cohorts does raise some concerns about the reliability and generalizability of the results. They should also carefully consider why the occurrence rate of enzymes involved in uremic toxins (tyrosine phenol-lyase, hpdC, fldHBC) showed no differences between ESRD-enriched and HC-enriched species. I would suggest that the authors carefully consider these discrepancies and provide a clear explanation for why they may have occurred. In addition, the authors should consider additional analyses to help validate their findings or explore other possible explanations for the differences in the results. This will help to ensure that the findings are robust and can be generalized to other cohorts.

**Response:** We express our sincere gratitude for your valuable comments. Regarding the differences in discriminant species between the two cohorts, there is actually a notable overlap in the most discriminant species for the toxins PAG, PCS, and IS between the Beijing and Shanghai cohorts, as detailed above. At a higher taxonomic level, Oscillospiraceae and Ruminococcaceae contribute significantly to PAG and PCS, while Bacteroidetes is a major contributor to IS. Variations in population characteristics, sample processing, and microbiota and toxin detection methods between the two cohorts, expectedly lead to different respective models. Nonetheless, importantly, we observed that models trained on one cohort's microbiota exhibited strong predictive performance for toxin levels in patients from the other cohort, particularly for IS, PAG, and PCS toxins, with an average R^2^ of 22.7% (ranging from 5.4% to 43%, equivalent to Pearson correlation coefficient r = 0.232-0.656; Figure 4a). Furthermore, to address concerns regarding the disparities in the most discriminant species between the two cohorts, we directly utilized the relative abundances of the identified 67 toxin-contributing species to train regression models in one cohort and tested these models in the other cohort. This analysis also demonstrated high cross-cohort predictability for IS, PAG, and PCS, with an average R^2^ of 29.2% (ranging from 10.1% to 51.9%, equivalent to Pearson correlation coefficient r = 0.318-0.720; see supporting figure below) in ESRD patients and an R^2^ of 26.8% (ranging from 3.7% to 50.8%, equivalent to Pearson correlation coefficient r = 0.192 – 0.713; Figure S18 in the revised manuscript). These findings indicate the feasibility of predicting serum toxin levels using a specific set of gut bacteria and suggest that these "toxin-contributing species" could serve as indicators or targets for interventions related to gut health in ESRD patients.

**Supporting figure.** Predicting the concentrations of toxins in ESRD patients based on 67 toxin-contributing species. The X-axis and Y-axis show the predicted concentration and scaled measured concentration for each toxin. Smooth curves are formed using the geom_smooth function with the default parameters in the R ggplot2 package.

Regarding the differences in enzymes involved in uremic toxins between ESRD-enriched and HC-enriched species, we have thoroughly examined and updated the results of Figure 3f in the revised manuscript based on the new list of enzymes and ESRD-associated species (refer to supporting figure 1). In this section of the results, we found that the occurrence rates of tryptophanase (producing indole), phenylacetaldehyde dehydrogenase (producing phenylacetate, the precursor of PAG), and choline trimethylamine-lyase (producing TMA) were significantly higher in ESRD-enriched species compared to HC-enriched species. However, 4-hydroxyphenylacetate decarboxylase (hpdC, producing p-cresol, the precursor of PCS) was only present in 6 ESRD-enriched species, but the difference was not significant (Fisher's exact test, *p*=0.089). On the other hand, the enzymes responsible for the production of two other toxins, tyrosine phenol-lyase (producing phenol) and phenyllactate dehydratase (*fldBC*, reducing aromatic amino acids to produce various toxin precursors), did not exhibit differences in occurrence rates between ESRD-enriched and HC-enriched species. At the species level, tyrosine phenol-lyase was primarily synthesized by *Alistipes spp.*, while ESRD-enriched species such as *Enterocloster spp.* and *Akkermansia muciniphila* also played a significant role (refer to supporting figure 2b). Phenyllactate dehydratase was mainly encoded by *Clostridium* and Oscillospiraceae species, with several members of Oscillospiraceae being enriched in ESRD patients. Furthermore, although the occurrence rates of these two enzymes did not differ between ESRD-enriched and HC-enriched species, when comparing their total abundances between patients and controls, we observed a trend towards enrichment in the fecal metagenome of ESRD patients. For example, tyrosine phenol-lyase was significantly higher in Beijing patients compared to healthy individuals, and phenyllactate dehydratase was significantly higher in Shanghai patients (refer to supporting figure 2a). The same trend was observed for the other four key enzymes involved in toxin production. This finding is consistent with the functional analysis, indicating that ESRD patients have higher levels of enzymes and modules associated with aromatic amino acid degradation compared to healthy controls (Figure 2b-c). Based on these collective results, we consider the conclusion that toxin-producing genes are more widely encoded in ESRD patients to be reliable. Therefore, in response to the reviewer's comments, we have made modifications to the relevant content in the "Functional configuration of ESRD-enriched and HC-enriched species" section in the main text and have added the results and description of toxin-producing genes. The Discussion section has also been modified accordingly.

|  | **Supporting figure 1 (part of Figure 3f in the manuscript).** Occurrence rate of enzymes involved in the biosynthesis of uremic toxins in the ESRD- (red) and HC-enriched (blue) species. |
| --- | --- |

**Supporting figure 2 (Figure S16 in the manuscript).** Distribution of the key synthetases involved in the biosynthesis of uremic toxins in fecal metagenomes. a, Relative abundance of key synthetase-encoding genes in ESRD patients and healthy controls. Statistical significance, as determined by the Wilcoxon rank-sum test between the patients and controls, is denoted by asterisks: *, *q*<0.05; **, *q*<0.01; ***, *q*<0.001. b, The mean weighted abundance of toxin-encoding genes in different gut species across fecal metagenomes. Each bar plot presents the top 20 species with the highest weighted abundance. The species are colored according to their enrichment in ESRD patients and healthy controls.

**Second round of review**

**Reviewer 1**

The authors have addressed my comments properly.

**Reviewer 2**

I appreciate the authors' thorough revisions in response to my previous feedback. It's clear that they have taken my suggestions and comments into consideration, which has significantly improved the quality of the manuscript. However, there are still a few areas that require further attention and modification to ensure the manuscript meets the desired standards.

Introduction: The introduction has been improved, however, there is room for further refinement to make it more concise and precise.

e.g.,

Line 29-Line 38. Try to not use “the stability” of the gut microbial community here.

Line 1-Line 11. “We performed metagenomic shotgun sequencing to analyze a total of 715 fecal samples, including 378 hemodialytic patients, 47 non-dialyzed CKD patients, and 290 healthy volunteers. These samples were obtained from two distinct Chinese cohorts: the previously described Beijing cohort [21], which was extended to include new ESRD patients and healthy controls, and a newly established Shanghai cohort.”

Try to revise this section to clearly describe the number of newly sequenced samples from the SH cohort and the number of previous sequenced samples from the BJ cohort.

Results:

“Microbial functions related to ESRD”

Figure 2D: The authors should consider revising Figure 2D, as it appears that a number of significant modules might be not derived from gut bacteria, e.g., M00687 MAPK signaling.

Table S9 and Fig. S9. The authors stated that “The ESRD patients exhibited an average 39.4% increase in the overall relative abundance of ARGs compared with the healthy controls (p<0.001; Additional file 2: Fig. S9a-b). This increase was particularly notable for several antibiotic resistance mechanisms, including multi-drug resistance, beta-lactamase, aminoglycoside, and quinolone resistance (Additional file 2: Fig. S9c). These findings suggest that patients may have experienced more frequent exposure to antibiotics in the past than the controls, possibly due to the treatment of dialysis-associated infections [29].”.

As the authors already have a newly collected subcohort including 47 non-dialyzed CKD patients, why not comparing the RAs of ARGs between dialyzed-ESRD patients, non-dialyzed CKD patients and healthy controls to assess the effects of frequent exposure to antibiotics on the enriched ARGs observed in patients?

“Functional configuration of ESRD-enriched and HC-enriched species”

Figure 3D. It appears that the comparisons between Bacteroidaceae, Prevotellaceae, Muribaculaceae and the “others” category may not be meaningful. To enhance the clarity of the figure, I recommend removing the “others” group.

“Gut bacteria affect serum uremic toxin levels” and the Rebuttal letter.

“The exception was TMAO, for which only 1.1%-2.2% of the concentration variances were explained by models trained on the opposite cohort (Fig. 4a), probably due to its high dialytic clearance rate [35] or dietary contributions to its levels [36].”

The authors have provided a comprehensive explanation for the limited overlap in the selected microbial features in explaining the variations of uremic toxin levels between two cohorts. However, an additional aspect worth considering is the potential presence of batch effects in toxin levels between the two cohorts, which might contribute to the observed differences in the predictive power of microbial features. Addressing this potential source of variation would provide a more comprehensive understanding of the factors affecting toxin levels and their associations with microbial features in the two cohorts.

Methods:

The author stated that “For fecal samples of the Shanghai cohort and the newly-sequenced Beijing samples, total bacterial DNA was extracted from approximately 200 mg of feces using standard methods [26].” Please note that this specific DNA extraction method used in [26] is not a standard method, which was developed over 15 years and has not been widely adopted in recent large-scale metagenomic studies. I strongly recommend that the authors thoroughly assess whether any modifications / adaptations have been made to the method in their work.

Rebuttal letter:

(1) The author stated that “In BJ cohort, the highest importance species included Fusobacterium ulcerans M398, Acutalibacteraceae M215, Eggerthella lenta M4B3, Massilimicrobiota M4B6, Erysipelatoclostridium spiroforme M16D, and others (ordered by mean decrease accuracy). Similarly, in the Shanghai population, the highest importance species comprised Intestinimona massiliensis M0D3, Lachnospiraceae M428, Anaerovoracaceae M0CF, Oscillospiraceae M4B8 and Prevotella M231, among others. It is worth noting that 38 out of the top 100 important species were shared between the two cohorts.”

To gain further insights into the performance of this shared subset, it would be valuable to assess the performance of the 38 taxa in distinguishing between patients and HCs in both cohorts. This analysis could provide a more in-depth understanding of the significance and consistency of these species across the cohorts. Please also provide data on the total abundances of the selected MAGs to the total assembled ones /total amount of sequencing data? This information would offer context regarding the prevalence and representation of the selected MAGs within the overall microbial community.

(2) The author stated that “Our analysis indicated that the highest ASW was achieved when the number of clusters was set to 5 (refer to the supporting figure below). However, it is important to note that even with the highest ASW, the value obtained was only 0.15, which falls considerably below the threshold typically considered reliable for stratification in classification (ASW = 0.30). Consequently, we concluded that there is insufficient evidence to support the presence of distinct clusters in the functional profiles of ESRD patients.”

I appreciate their additional analysis on this point. However, the five distinct clusters might be influenced by the combination of patients from both the BJ and SH cohorts. Please provide the number of patients from two cohorts within each cluster. Otherwise, it would indeed be informative to re-conduct the clustering analysis separately for each cohort. This step would allow for a more detailed examination of clustering patterns within each cohort.

**Authors Response**

**Point-to-point response to the reviewers:**

Reviewer #1: The authors have addressed my comments properly.

**Response:** We appreciate the reviewer’s enthusiasm and helpful comments.

Reviewer #2: I appreciate the authors' thorough revisions in response to my previous feedback. It's clear that they have taken my suggestions and comments into consideration, which has significantly improved the quality of the manuscript. However, there are still a few areas that require further attention and modification to ensure the manuscript meets the desired standards.

**Response:** We sincerely appreciate your keen interest and the valuable insights you have provided. Please find below our responses addressing your concerns.

Introduction: The introduction has been improved, however, there is room for further refinement to make it more concise and precise.

e.g.,

Line 29-Line 38. Try to not use “the stability” of the gut microbial community here.

**Response:** Thanks for pointing this out. As per your suggestion, we have revised the expression in the revised manuscript. The modified content is as follows:

“*Moreover, the gut microbial community helps maintain individual metabolic and immune homeostasis, which may be beneficial for reducing the risk of complications…*”

Line 1-Line 11. “*We performed metagenomic shotgun sequencing to analyze a total of 715 fecal samples, including 378 hemodialytic patients, 47 non-dialyzed CKD patients, and 290 healthy volunteers. These samples were obtained from two distinct Chinese cohorts: the previously described Beijing cohort [21], which was extended to include new ESRD patients and healthy controls, and a newly established Shanghai cohort.*”

Try to revise this section to clearly describe the number of newly sequenced samples from the SH cohort and the number of previous sequenced samples from the BJ cohort.

**Response:** Thank you for providing this valuable comment. Following your suggestion, we have modified these contents in the revised manuscript as follows:

“*We performed metagenomic shotgun sequencing to analyze a total of 715 fecal samples, including 378 hemodialytic patients, 47 non-dialyzed CKD patients, and 290 healthy volunteers. These samples were obtained from two distinct Chinese cohorts: the previously described Beijing cohort [21], which was extended to include new ESRD patients and healthy controls (totaling 254 ESRD patients and 179 controls), and a newly established Shanghai cohort (124 ESRD patients, 47 non-dialyzed CKD patients, and 111 controls).*”

Results:

“*Microbial functions related to ESRD*”

Figure 2D: The authors should consider revising Figure 2D, as it appears that a number of significant modules might be not derived from gut bacteria, e.g., M00687 MAPK signaling.

**Response:** We greatly appreciate your insightful comments. Following your suggestion, we have implemented revisions to the criteria governing the classification of KEGG orthologs into modules. Notably, we have excluded modules originating from sources other than the gut microbiota, such as those derived from animals or plants. These modifications have been integrated into Figure 2D and Table S8 in the revised manuscript.

Table S9 and Fig. S9. The authors stated that “*The ESRD patients exhibited an average 39.4% increase in the overall relative abundance of ARGs compared with the healthy controls (p<0.001; Additional file 2: Fig. S9a-b). This increase was particularly notable for several antibiotic resistance mechanisms, including multi-drug resistance, beta-lactamase, aminoglycoside, and quinolone resistance (Additional file 2: Fig. S9c). These findings suggest that patients may have experienced more frequent exposure to antibiotics in the past than the controls, possibly due to the treatment of dialysis-associated infections [29].*”.

As the authors already have a newly collected subcohort including 47 non-dialyzed CKD patients, why not comparing the RAs of ARGs between dialyzed-ESRD patients, non-dialyzed CKD patients and healthy controls to assess the effects of frequent exposure to antibiotics on the enriched ARGs observed in patients?

**Response:** We appreciate the valuable comment from the reviewer. Based on your suggestion, we have introduced a comparison involving 47 non-dialyzed CKD patients. The results are depicted in the supporting figure below (upper panel). We observed a substantial 59.8% increase in the overall relative abundance of ARGs in these CKD patients when compared with healthy controls (*p* < 0.001). Specifically, the total ARG abundance in non-dialyzed CKD patients also surpassed that in ESRD patients, showing a 15.4% increase (*p* = 0.013). In terms of antibiotic resistance mechanisms, non-dialyzed CKD patients exhibited notably higher levels in several mechanisms compared to healthy individuals (supporting figure: lower panel). These findings provide further evidence that, akin to ESRD patients, non-dialyzed CKD patients may have been subjected to more frequent antibiotic exposure in the past than the control group. Consequently, we have integrated these results into Figure S9b-c and modified the "*Microbial functions related to ESRD*" section of the revised manuscript accordingly.

**Supporting figure (Figure S9b-c in the revised manuscript).** Boxplot showing the comparison of total abundance (upper panel) and abundance of each ARG category (lower panel) among ESRD patients, non-dialyzed CKD patients, and healthy controls. Student’s t test: *, q<0.05; **, q<0.01; ***, q<0.001. RPKM, reads per kilobase per million mapped reads.

“*Functional configuration of ESRD-enriched and HC-enriched species*”

Figure 3D. It appears that the comparisons between Bacteroidaceae, Prevotellaceae, Muribaculaceae and the “others” category may not be meaningful. To enhance the clarity of the figure, I recommend removing the “others” group.

**Response:** Thanks for your helpful comment. We have removed the “others” group in Figure 3D in the revised manuscript following your suggestion.

“Gut bacteria affect serum uremic toxin levels” and the Rebuttal letter.

“*The exception was TMAO, for which only 1.1%-2.2% of the concentration variances were explained by models trained on the opposite cohort (Fig. 4a), probably due to its high dialytic clearance rate [35] or dietary contributions to its levels [36].*”

The authors have provided a comprehensive explanation for the limited overlap in the selected microbial features in explaining the variations of uremic toxin levels between two cohorts. However, an additional aspect worth considering is the potential presence of batch effects in toxin levels between the two cohorts, which might contribute to the observed differences in the predictive power of microbial features. Addressing this potential source of variation would provide a more comprehensive understanding of the factors affecting toxin levels and their associations with microbial features in the two cohorts.

**Response:** Thank you for this insightful comment. As pointed out by the reviewer, batch effects indeed appear to play a significant contributing factor in the observed differences between the two cohorts. As demonstrated in Figure 4a (also shown in the supporting table below), the mutual predictability of serum concentrations for all four toxins (i.e., IS, PAG, PCS, and TMAO) between the two cohorts (influenced by both population and batch effects) is remarkedly lower than the interpretability of these toxins within the same cohort (unaffected by batch-specific influences). However, it is evident that TMAO is more profoundly impacted by cross-cohort effects than the other three toxins (supporting table below). Therefore, apart from population and batch effects, the unpredictability of TMAO levels may also be linked to its rapid clearance rate during dialysis, dietary sources, or other factors. Nevertheless, as per the reviewer's suggestion, in the revised manuscript, we have emphasized the influence of batch effects in this sentence as follows:

“*The exception was TMAO, for which only 1.1%-2.2% of the concentration variances were explained by models trained on the opposite cohort (Fig. 4a), probably due, in part, to* ***batch effects*** *or the its high dialytic clearance rate [35] or dietary contributions to its levels [36] of this toxin.*”

|  | **Supporting table.** Predictability of concentration variances of four toxins by the models trained by Beijing and Shanghai cohorts. See Figure 4a for details. |
| --- | --- |

Methods:

The author stated that “*For fecal samples of the Shanghai cohort and the newly-sequenced Beijing samples, total bacterial DNA was extracted from approximately 200 mg of feces using standard methods [26].*” Please note that this specific DNA extraction method used in [26] is not a standard method, which was developed over 15 years and has not been widely adopted in recent large-scale metagenomic studies. I strongly recommend that the authors thoroughly assess whether any modifications / adaptations have been made to the method in their work.

**Response:** Thanks for this valuable comment. In fact, our DNA extraction method was based on previous techniques but underwent certain modifications. As per your suggestion, in the revised manuscript, we have extensively described the DNA extraction methods. The revised content is provided below:

“*Total microbial DNA were extracted from approximately 200 mg of fecal samples using the HiPure Stool DNA Kit (Magen, Guangzhou, China) and operations followed the specification. Briefly, STL buffer (1ml) were added to 50mg of sample in a 2ml screw cap tubes prior to incubation at 65 °C for 10 min. After samples were put under vortex movement (15s) and centrifugation (13,000× g, 10 min), 600μl of supernatants were transferred to fresh 2.0 ml tubes. Samples were then added 150µl of PS buffer and 150µl of absorber solution. Following a second centrifugation (13,000× g, 5 min), the supernatants were placed in fresh 2.0 ml tubes where 700µl of GDP buffer were added. Lastly, HiPure DNA Mini Column (Magen, Guangzhou, China) was used to absorb products and washed with sterile water.*”

Rebuttal letter:

(1) The author stated that “*In BJ cohort, the highest importance species included Fusobacterium ulcerans M398, Acutalibacteraceae M215, Eggerthella lenta M4B3, Massilimicrobiota M4B6, Erysipelatoclostridium spiroforme M16D, and others (ordered by mean decrease accuracy). Similarly, in the Shanghai population, the highest importance species comprised Intestinimona massiliensis M0D3, Lachnospiraceae M428, Anaerovoracaceae M0CF, Oscillospiraceae M4B8 and Prevotella M231, among others. It is worth noting that 38 out of the top 100 important species were shared between the two cohorts.*”

To gain further insights into the performance of this shared subset, it would be valuable to assess the performance of the 38 taxa in distinguishing between patients and HCs in both cohorts. This analysis could provide a more in-depth understanding of the significance and consistency of these species across the cohorts. Please also provide data on the total abundances of the selected MAGs to the total assembled ones /total amount of sequencing data? This information would offer context regarding the prevalence and representation of the selected MAGs within the overall microbial community.

**Response:** We greatly appreciate the constructive comment from the reviewer. Based on your suggestions, we have developed random forest classification models using the top 38 most important species that are shared between both cohorts, and subsequently performed predictive analyses. The results indicate favorable predictive performance of these 38 species in both cohorts (AUC = 0.917-0.962; see supporting figure below). Furthermore, as per your suggestion, we have added the average relative abundances of each species in Table S5 of the revised manuscript. This analysis revealed that the top 100 most important species collectively accounted a total abundance of 10.9% and 12.7% in the Shanghai and Beijing cohorts, respectively.

|  | **Supporting figure.** ROC analysis of the classification of ESRD status using the random forest model based on 38 shared species. For each cohort, 70% of the samples were randomly selected as the training set, and the remaining 30% of the samples were used as the testing set. The performances of models trained on one cohort and tested on the other are also shown. The classification performance of the model was assessed using the area under the ROC curve (AUC). The AUC values and 95% confidence intervals (CIs) are shown. |
| --- | --- |

(2) The author stated that “*Our analysis indicated that the highest ASW was achieved when the number of clusters was set to 5 (refer to the supporting figure below). However, it is important to note that even with the highest ASW, the value obtained was only 0.15, which falls considerably below the threshold typically considered reliable for stratification in classification (ASW = 0.30). Consequently, we concluded that there is insufficient evidence to support the presence of distinct clusters in the functional profiles of ESRD patients*.”

I appreciate their additional analysis on this point. However, the five distinct clusters might be influenced by the combination of patients from both the BJ and SH cohorts. Please provide the number of patients from two cohorts within each cluster. Otherwise, it would indeed be informative to re-conduct the clustering analysis separately for each cohort. This step would allow for a more detailed examination of clustering patterns within each cohort.

**Response:** We greatly appreciate the reviewer's insightful comments. Based on your suggestion, we have investigated the distribution of individuals across 5 clusters within both Beijing and Shanghai populations (see supporting table below) and found no significant differences in the cluster distribution between the two cohorts. Furthermore, we re-conducted clustering analysis based on functional profiles within each cohort, and the new results are shown in the supporting figure below. Evidently, the Beijing and Shanghai cohorts exhibit subdivision into 4 and 5 clusters, respectively. However, consistent with previous findings, the highest average silhouette score (ASW) for clustering in both cohorts remains below 0.30 (0.16 and 0.11, respectively). Consequently, this analysis reaffirms our earlier conclusion, emphasizing the lack of compelling evidence supporting the presence of distinct clusters in the functional profiles of ESRD patients.

|  | **Supporting table.** Number of samples from two cohorts within each cluster. |
| --- | --- |

**Supporting figure.** PCoA analysis of the functional composition of ESRD patients in the Beijing (right panel) and Shanghai (left panel) cohorts. PCoA plot showing the clustering results, and samples are colored based on 4 (Beijing) or 5 (Shanghai) optimal clusters. The first two principal coordinates and their explained variances are shown.
